# The floral illusion: A parasitic beetle mimics the scent of flowers to attract bees

**DOI:** 10.1073/pnas.2602521123

**Published:** 2026-07-28

**Authors:** Ryan M. Alam, Danny Kessler, Heiko Vogel, Katrin Luck, Anja David, Maritta Kunert, Gunnar Brehm, Martin Kaltenpoth, Sarah E. O’Connor, Tobias G. Köllner

**Affiliations:** ^a^ Department of Natural Product Biosynthesis, Max Planck Institute for Chemical Ecology, Jena D-07745, Germany; ^b^ Department of Insect Symbiosis, Max Planck Institute for Chemical Ecology, Jena D-07745, Germany; ^c^ Institute for Zoology and Evolutionary Biology, Phyletic Museum, Friedrich Schiller University Jena, Jena D-07743, Germany

**Keywords:** blister beetle, mimicry, floral volatile, P450

## Abstract

Animals are not known to biosynthesize floral chemical signals to manipulate pollinators, although such mimicry could profoundly shape plant–pollinator interactions. Larvae of the poisonous European blister beetle *Meloe proscarabaeus* parasitize multiple solitary bee species, yet the mechanism enabling host attraction has remained unresolved. Here we show that these larvae lure bees by emitting a bouquet of volatile compounds that closely resembles floral scent. Chemical analyses reveal a complex blend of monoterpenoids derived from (*S*)-linalool, a ubiquitous floral volatile. Behavioral assays demonstrate that these compounds function as floral-scent mimics, eliciting attraction in bees and acting as allomones (i.e., interspecific chemical signals that benefit the emitter while disadvantaging the receiver). Transcriptomic and functional analyses identify cytochrome P450 enzymes that oxidize (*S*)-linalool, suggesting that larvae biosynthesize these plant-like volatiles de novo. Together, these findings broaden the scope of interkingdom chemical mimicry and uncover a striking form of sensory deception in which an insect chemically assumes the signal identity of a flower, revealing that animals can evolve biosynthetic pathways to exploit plant–pollinator communication.

## Significance

Flowers use scent to attract pollinators, yet animals are not known to produce floral chemical signals to manipulate these interactions. Here we show that larvae of the parasitic blister beetle *Meloe proscarabaeus* chemically mimic flowers by biosynthesizing a complex bouquet of floral volatiles. These larvae emit (*S*)-linalool-derived monoterpenoids, compounds widespread in angiosperm floral scent, known to attract a broad range of bee species. Behavioral experiments demonstrate that these volatiles function as floral scent mimics, while transcriptomic and functional analyses reveal cytochrome P450 enzymes responsible for their de novo biosynthesis. Our findings uncover a striking form of interkingdom chemical mimicry, showing that animals can evolve plant-like biosynthetic pathways to exploit pollinator sensory systems and reshape plant–pollinator communication.

Mimicry, broadly defined as deceptive resemblance, is a widespread evolutionary strategy utilized across the tree of life (1). Since Bates’ seminal observations of visual mimicry among Amazonian butterflies in 1862 (2), the concept has broadened to include deception across all major sensory modalities, including visual (3–5), tactile (6), acoustic (7), and chemical (8–12). Although many studies focus on mimicry in predator–prey or host–parasite interactions (13–15), deceptive resemblance also plays key roles in other interspecific relationships. One striking example is phoresy, where one organism (the phoront) uses another (the host) for transport to essential resources (16). Phoronts frequently evolve morphological, behavioral, and/or chemical traits that imitate benign or mutualistic species, deceiving hosts into providing transport (8, 17–19). Moreover, phoresy has been proposed as an evolutionary precursor to parasitism in certain systems (16), and complete dependence on a host for dispersal can further drive the emergence of obligate parasitic relationships (8).

**Fig. 1.**
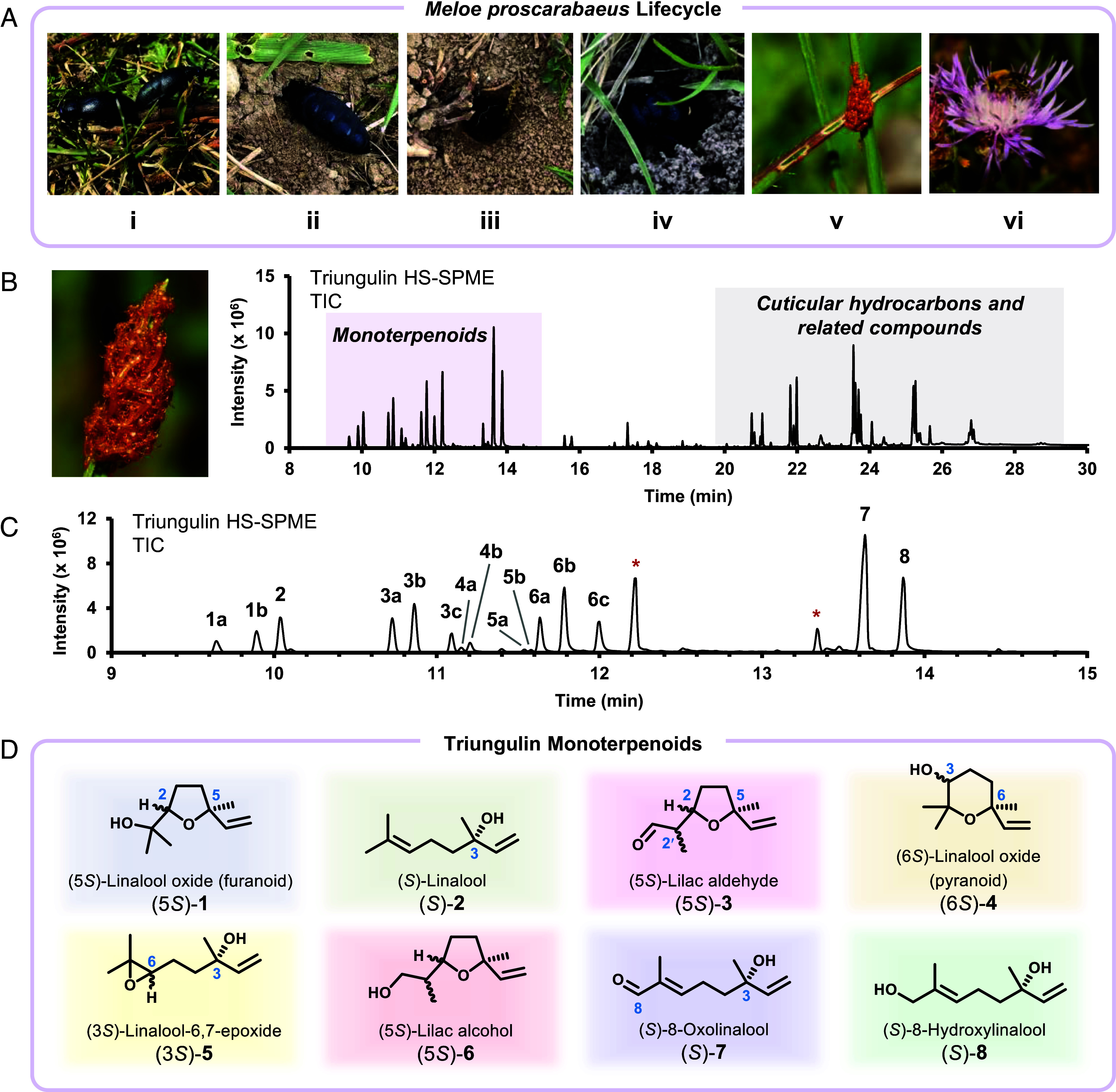
Lifecycle and volatile emissions of *Meloe proscarabaeus* triungula. (*A*) Lifecycle of the toxic European Black oil beetle *M*. *proscarabaeus*. After emerging and mating in the spring (*i*), gravid females dig underground chambers (*ii* and *iii*) and oviposit thousands of yellow eggs (*iv*). Triungula hatch after several weeks and form conspicuous orange aggregations on vegetation (*v*), where they await contact with a solitary bee host that inadvertently carries them to their nest to complete their development to adulthood (*vi*). (*B*) Total ion chromatogram (TIC) of triungulin volatile profile, obtained via HS–SPME collection and GC–MS analysis. (*C*) Alongside long-chain hydrocarbons (t*_r_* 20.95 to 29.22 min), *M*. *proscarabaeus* triungula emit a complex bouquet of floral-scent monoterpenoids (t*_r_* 9.83 to 14.05 min, 1–8). Peak identification: (*1a* and *1b*) (5*S*)-linalool oxide (furanoid)^‡^, (*2*) (*S*)-linalool, (*3* *a*–*c*) (5*S*)-lilac aldehyde^‡^, (*4a* and *4b*) (6*S*)-linalool oxide (pyranoid)^‡^, (*5a* and *5b*) (3*S*)-linalool-6,7-epoxide^‡^, (*6a*–*c*) (5*S*)-lilac alcohol^‡^, (*7*) (*S*)-8-oxolinalool, (*8*) (*S*)-8-hydroxylinalool. *Background; ^‡^Mixture of stereoisomers. (*D*) Structural and stereochemical assignments of identified monoterpene volatiles were confirmed by comparison with synthetic standards using GC–MS on both achiral and chiral phases.

Blister beetles (Coleoptera: Meloidae) exemplify the intersection of mimicry, phoresy, and parasitism. Most species parasitize solitary bees (20). In the subfamily Meloinae, gravid females deposit large egg clutches underground; the eclosed larvae (triungula) are highly mobile and either climb nearby vegetation to await hosts (Fig. 1*A*) (8, 21, 22) or, in nonphoretic species, actively search for bee nests on the soil surface (23). These larvae commonly target solitary bees, rapidly attaching to them, and are then transferred to the bee nest. Once in the nest, triungula dismount, consume the bee egg and provisions, and later complete several distinct developmental stages before emerging as adults the following year (24).

Host-attraction strategies in kleptoparasitic phoretic *Meloe* species vary and often exploit host-specific sensory cues. For instance, *Meloe franciscanus* triungula in North America aggregate on vegetation and emit a blend of female bee sex pheromones to specifically attract male *Habropoda* spp. (8, 22). Alternatively, *M. strigulosus* larvae station themselves on flowers, where they intercept foraging pollinators (21). In contrast, triungula of the European Black oil beetle *M*. *proscarabaeus* typically form conspicuous orange aggregations on grasses and exhibit little host specificity, suggesting a generalist phoretic strategy (25). This behavioral divergence led us to hypothesize that *M*. *proscarabaeus* triungula use an alternative volatile cue to attract a broad range of pollinator hosts. Here, we demonstrate that *M*. *proscarabaeus* larvae emit a complex bouquet of floral-scent monoterpenoids to deceive foraging bees. This finding reveals a previously undescribed form of interkingdom chemical mimicry and expands the conceptual framework of sensory deception and phoretic host attraction.

## Results and Discussion

### Larvae Emit Floral Monoterpenoids.

To characterize volatile emissions from *M*. *proscarabaeus* triungula, adult beetles were collected in early spring (February–April 2024–25) from Jena, Thuringia, Germany, and maintained under controlled conditions for mating and oviposition (*SI Appendix*, Fig. S1). After approximately 3 wk, egg clutches hatched synchronously, and aggregating triungula were harvested for volatile analysis (*SI Appendix*, Fig. S2). Headspace solid-phase microextraction (HS–SPME) coupled with gas chromatography-electron ionization-mass spectrometry (GC–MS) revealed a complex volatile profile, comprising more than thirty C_19_–C_27_ saturated and unsaturated long-chain hydrocarbons (t*_r_* 20.95 to 29.22 min; Fig. 1*B* and *SI Appendix*, Fig. S3), similar to compounds previously reported in *M*. *franciscanus* larvae (22). However, in addition to these hydrocarbons, we detected several structurally distinct monoterpenoids (t*_r_* 9.83 to 14.05 min; Fig. 1 *C* and *D*), including *cis*- and *trans*- diastereoisomers of linalool oxide (furanoid) (**1**), linalool (**2**), multiple stereoisomers of lilac aldehyde (**3**), linalool oxide (pyranoid) (**4**), linalool-6,7-epoxide (**5**), and lilac alcohol (**6**), and 8-oxolinalool (**7**) and 8-hydroxylinalool (**8**). Compound identities (**1**–**8**) were confirmed by comparison with synthesized reference standards (*SI Appendix*, Fig. S4). Notably, unlike *M. franciscanus* triungula, whose volatile profiles vary among geographically distant populations (22), larvae from an allopatric population of *M*. *proscarabaeus* collected in Kiel, Schleswig-Holstein, Germany (Spring 2026) showed no observable differences in monoterpenoid composition relative to those from Jena (*SI Appendix*, Fig. S5).

GC–MS analysis on chiral phases revealed that *M*. *proscarabaeus* triungula exclusively emit the (*S*)- enantiomer of linalool [(*S*)-**2**] (*SI Appendix*, Figs. S6 and S7) along with a range of (*S*)-**2**-derived metabolites bearing conserved stereochemistry at the C-3 position (Fig. 1*D* and *SI Appendix*, Figs. S8–S21). Absolute configurations were verified by comparison with synthetic reference standards derived from *rac*-, (*R*)-, and (*S*)-**2**. In total, triungula emitted 17 structurally distinct monoterpenoids (**1**–**8**), all of which are known floral volatiles widespread among angiosperms (26, 27) such as *Berberis vulgaris*, *Prunus* spp., and *Salix* spp. (28–31), which serve as the main food source for pollinating bees in the spring, when *M. proscarabaeus* triungula also emerge. However, except for (*S*)-**2** (32, 33) and linalool oxide **1** (33, 34), none of these monoterpenoids have been previously reported to be produced in an insect.

### The Scent of Live Triungula Attracts Wild Bees Via Floral-Scent Mimicry.

Several monoterpenoids emitted by *M*. *proscarabaeus* triungula—including (*S*)-linalool [(*S*)-**2**] and its derivatives (**1** and **3**–**8**)—are common floral volatiles known to attract pollinators, including solitary wild bees (28–31, 35). We hypothesized that the production and emission of these volatiles enable triungula to mimic floral scent and thereby attract a broad range of phoretic hosts. This reasoning is supported by reports that *M*. *proscarabaeus* larvae parasitize ground-nesting solitary bees, including *Andrena* spp. and *Colletes* spp. (20), while triungula have also been recovered from nests of social bee species, which are not considered suitable hosts because they remove transmitted larvae from the nest before the larvae can complete development to adulthood (20, 36, 37). Notably, the occurrence of *M. proscarabaeus* triungula on both suitable and unsuitable bee hosts suggests that these larvae may employ a relatively broad host-attraction strategy.

**Fig. 2.**
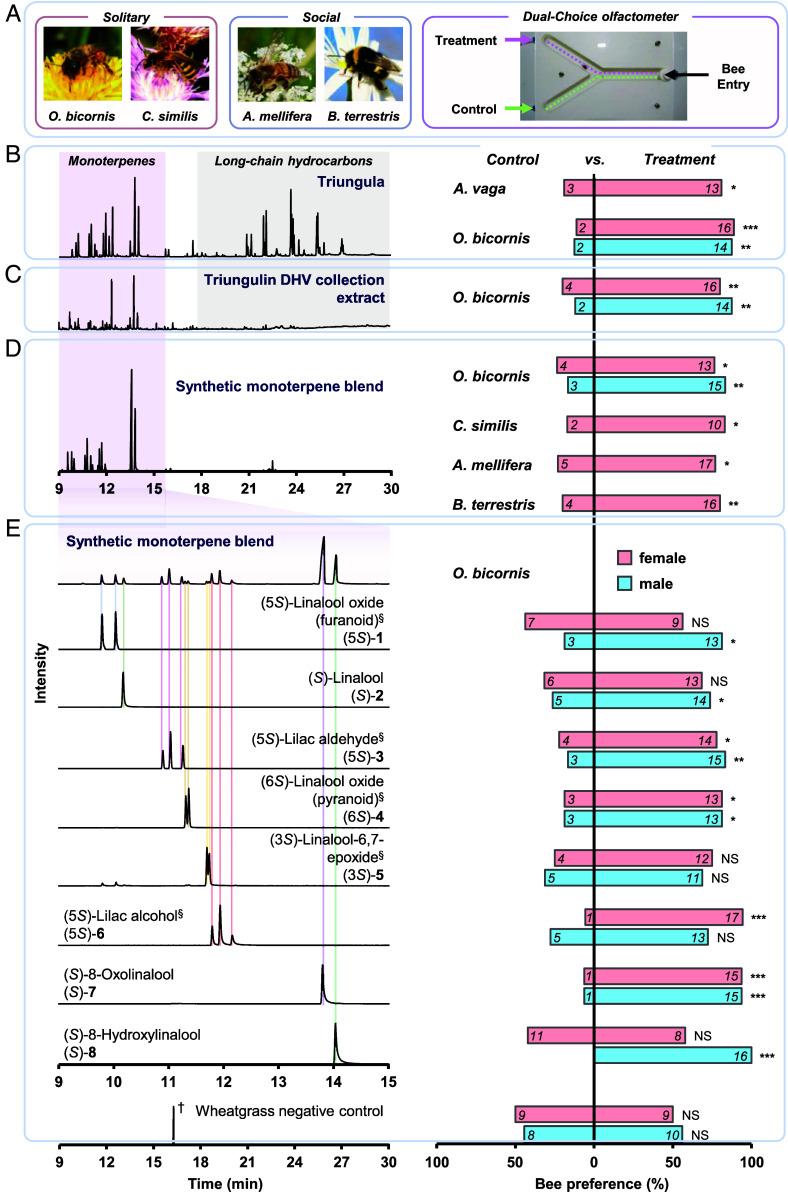
Triungulin-derived monoterpenoids attract solitary and social bees. (*A*) Dual-choice Y-tube behavioral assays were performed with solitary (*A. vaga*, *O. bicornis*, and *Colletes similis*) and social (*Apis mellifera* and *Bombus terrestris*) bee species. (*B*) Wild-caught female *A. vaga* and male and female *O*. *bicornis* were attracted to live triungula. (*C*) Volatile extracts from aggregating triungula, obtained via dynamic headspace volatile (DHV) collection, also attracted male and female *O*. *bicornis*. (*D*) A synthetic blend of (*S*)-linalool [(*S*)-**2**]-derived monoterpenoids (**1**–**8**) elicited significant attraction from female *C. similis*, *A*. *mellifera*, *B*. *terrestris*, and both sexes of *O*. *bicornis*. (*E*) Behavioral assays with individual monoterpenoids showed sex-specific responses in *O*. *bicornis*. Control assays with wheat in both Y-tube arms confirmed no side bias. All bees, except *C*. *similis*, were flower-naïve. For further information see *SI Appendix*, Tables S1–S29. Numbers in bars indicate absolute choices. χ^2^ test: ***P < 0.001, **P < 0.01, *P < 0.05, ^NS^*P* > 0.05. NS = not significant. ^§^Mixture of stereoisomers; ^†^Geranylacetone; tentatively identified using the NIST MS-Library v. 3.0 (2023).

To test whether *M*. *proscarabaeus* triungulin monoterpenoids attract solitary bees, we first conducted dual-choice olfactometer assays with the proposed oligolectic host *Andrena vaga* and a polylectic wild bee species *Osmia bicornis* (Fig. 2*A* and *SI Appendix*, Fig. S22). Live triungula, emitting a complex blend of monoterpenoids and long-chain hydrocarbons, significantly attracted wild-caught female *A. vaga* as well as male and female *O*. *bicornis* (Fig. 2*B*). Although *O. bicornis* is not known to represent a suitable host for *M. proscarabaeus*, this species was used in subsequent behavioral assays as it was commercially available in large numbers and provided a tractable model for controlled behavioral assays. Importantly, attraction of *O*. *bicornis* was consistent with our hypothesis that triungulin monoterpenoids function as generalized floral cues. For example, volatile extracts obtained via dynamic headspace volatile (DHV) collection—which contained markedly reduced amounts of long-chain hydrocarbons—also attracted both sexes of *O*. *bicornis* (Fig. 2*C*). Notably, DHV collection captured the full triungulin monoterpenoid profile, whereas parallel solvent-based extractions failed to recover these volatiles (*SI Appendix*, Fig. S23).

### Synthetic Blends of Larval Monoterpenoids Attract Solitary and Social Bees.

As previously noted, *M*. *franciscanus* larvae attract male solitary bees by emitting a blend of long-chain hydrocarbons that mimic female bee sex pheromones (22). Having established that DHV-derived monoterpenoid blends containing reduced amounts of long-chain hydrocarbons mediate attraction, we next tested whether synthetic monoterpenoids alone were sufficient to elicit attraction. To this end, we exposed male and female *O*. *bicornis* to a synthetic mixture of (*S*)-linalool derivatives (compounds **1**–**8**; Fig. 2*C* and *SI Appendix*, Fig. S24*A*). Both sexes were significantly attracted to this blend (χ^2^ test, *P* < 0.05). In contrast, only males, but not females, responded to an analogous blend of synthetic (*R*)-linalool-derived monoterpenoids (*SI Appendix*, Fig. S24*B*), highlighting the importance of the natural stereoisomeric composition of the triungulin volatile bouquet.

Considering that *O*. *bicornis* is likely an unsuitable host for *M*. *proscarabaeus* triungula—as it typically nests in preexisting cavities in wood, hollow stems, loess, clay, or masonry (38)—we next tested the synthetic (*S*)-**2**-derived blend in dual-choice assays with wild-caught female *C. similis*, a ground-nesting solitary bee, and with workers of the eusocial generalists *B. terrestris* and *A. mellifera* (Fig. 2*D*). These assays confirmed the attraction of *C. similis* and additionally revealed that triungula can also attract potentially unsuitable social bee hosts. Although phoresy on such vectors may not result in successful development, broad responsiveness to floral-associated cues may nevertheless be adaptive in environments where encounters with suitable hosts are infrequent or unpredictable. Under such conditions, selection may favor a generalized attraction strategy that maximizes overall host contact rates, even at the cost of occasional attachment to unsuitable vectors. Similar tradeoffs have been proposed in other deceptive signaling systems in which sensory exploitation increases encounter frequency but reduces receiver specificity (39, 40). Consistent with this interpretation, *M*. *strigulosus* triungula have been observed to detach from unsuitable hosts during interfloral movements (21), suggesting that transient attachment to nonhost pollinators may facilitate dispersal and increase opportunities for encounters with suitable hosts.

### Individual Synthetic Monoterpenoids Directly Attract Female Solitary Bees.

We next investigated each larval monoterpenoid compound (**1**–**8**) individually in dual-choice assays using naïve male and female *O*. *bicornis* (Fig. 2*D*). All monoterpenoids except (3*S*)-linalool-6,7-epoxide [(3*S*)-**5**] and (5*S*)-lilac alcohol [(5*S*)-**6**] elicited significant attraction in males (χ^2^ test, 0.001 > *P* < 0.05). Females were not attracted to (3*S*)-**5** and, unlike males, showed no preference for (*S*)-**2**, (5*S*)-linalool oxide (furanoid) [(5*S*)-**1**], or (*S*)-8-hydroxylinalool [(*S*)-**8**] (*P* > 0.05). However, (5*S*)-lilac aldehyde [(5*S*)-**3**], (6*S*)-linalool oxide (pyranoid) (6*S*-**4**), (5*S*-**6**), and (*S*)-8-oxolinalool [(*S*)-**7**] were significantly attractive to females (0.01 > *P* < 0.05). The observed attraction of female bees to the monoterpenoid blend as well as several of the individual components support the hypothesis that mimicry of floral scent in *M*. *proscarabaeus* larvae allows them to directly lure female hosts, unlike triungula of *M*. *franciscanus* who only attract intermediary male *Habropoda* bees and then have to move to females during copulation in order to reach the nest. The direct attraction of female bee species by *M*. *proscarabaeus* larvae likely enhances their chances of nest entry and successful development.

### Wild Bees are Attracted by Larval Monoterpenoids in the Field.

**Fig. 3.**
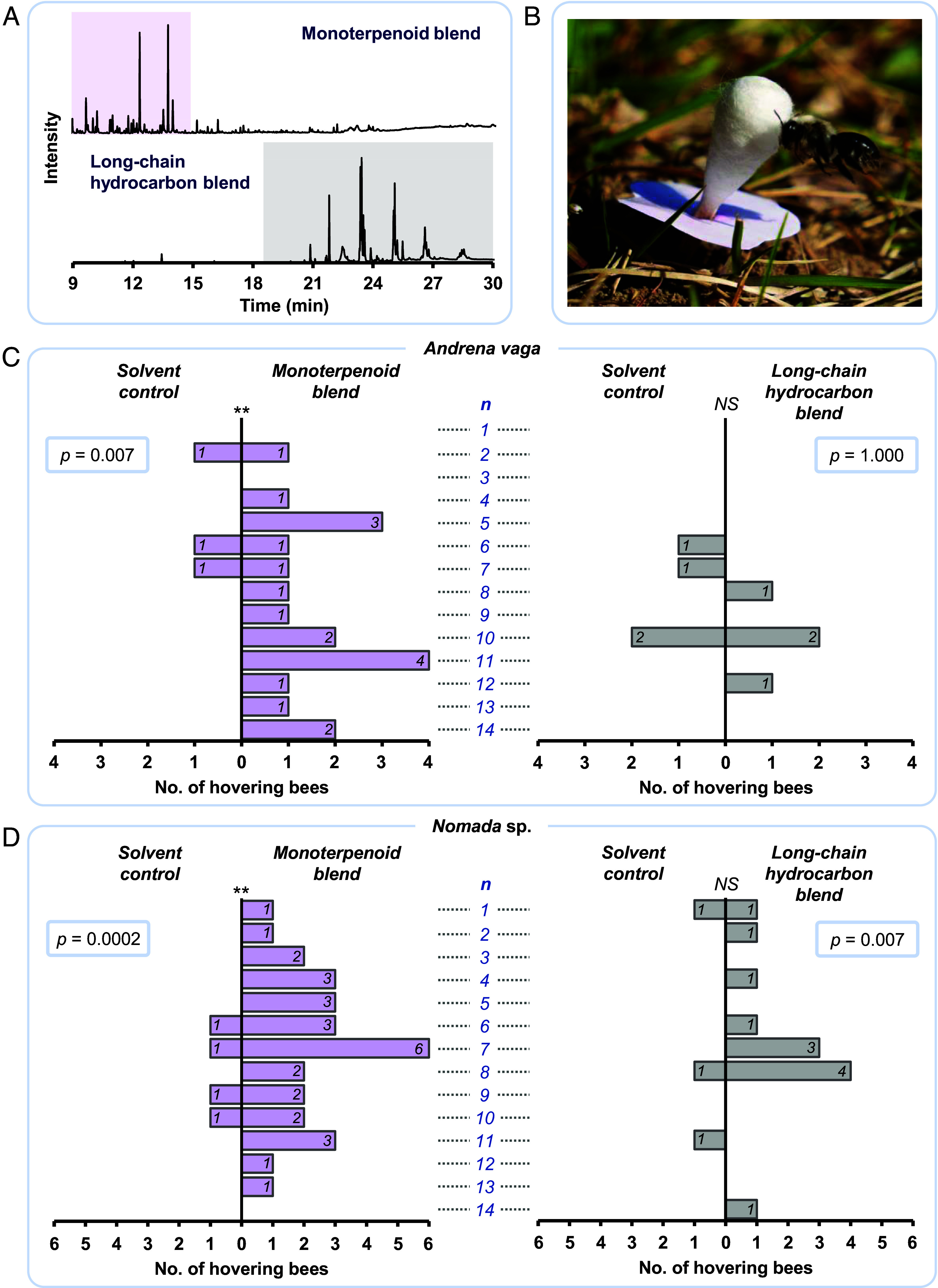
Floral-scent monoterpenoids, but not long-chain hydrocarbons, mediate attraction of phoretic bee hosts to *M. proscarabaeus* in the field. (*A*) GC–MS chromatographic data for DHS volatile collection extracts from aggregating triungula containing the (*S*)-linalool-derived monoterpenoid blend (upper chromatogram) and a whole-body pentane extract containing larval long-chain hydrocarbons (lower chromatogram). (*B*) Observational field attraction assays were carried out using cotton swaps as artificial flowers that were dosed with treatment or solvent control (*SI Appendix*, Fig. S26). (*C*) Field assays with the reported host *A*. *vaga* revealed significant attraction to the DHV-derived monoterpenoid blend, whereas long-chain hydrocarbon extracts elicited no significant attraction. (*D*) The monoterpenoid blend also attracted a cuckoo bee *Nomada sp.*, while long-chain hydrocarbons again were not significantly attractive. Numbers in bars indicate the absolute number of hovering bees observed in front of the artificial flower dosed with treatment of solvent control. two-sided Wilcoxon signed-rank test: *P < 0.05, ^NS^*P* > 0.05. NS = not significant.

While the sex pheromones of *A*. *mellifera* and *B*. *terrestris* have been described previously as long-chain hydrocarbon derivatives (41, 42) and do not include monoterpenoids, certain colletid bees (43), such as *Colletes* *cunicularius* (32), use (*S*)-**2** as a mate attractant. However, we could not detect monoterpenoids in either sex of *A. vaga*, *C. similis,* or *O. bicornis* (*SI Appendix*, Fig. S25). Together with the observation that triungulin monoterpenoids attract several distantly related bee species, this supports the conclusion that bee attraction to triungulin monoterpenoids is based on floral scent mimicry rather than the imitation of bee sex pheromones. To further investigate which components of the larval volatile emission mediate attraction, we subsequently conducted observational field attraction assays using the DHV-derived monoterpenoid blend and long-chain hydrocarbon extracts separately (Fig. 3 *A* and *B* and *SI Appendix*, Fig. S26*A*). The monoterpenoid blend significantly attracted the reported solitary bee host *A. vaga* and, interestingly, also a kleptoparasitic cuckoo bee species (*Nomada* sp.; *SI Appendix*, Fig. S26*B*) known to target *Andrena* spp. (44). In contrast, long-chain hydrocarbon extracts failed to elicit significant attraction (Wilcoxon signed-rank test, *P* < 0.01 and *P* > 0.5, respectively) (Fig. 3*C*). These findings support the hypothesis that volatile monoterpenoids are sufficient to mediate attraction of phoretic hosts. It remains speculative whether the observed attraction of cuckoo bees, which could potentially transport the beetle larvae directly into the nests of their shared host bees, also serves as a mechanism for the phoretic dispersal of *M. proscarabaeus* larvae, and this question should be addressed in future studies.

Altogether, the interpretation of our results aligns with theoretical frameworks of deceptive signaling that emphasize exploitation of preexisting sensory biases rather than strict “one-to-one” mimicry of specific models (39). These findings further indicate that deceptive signaling systems need not evolve through strict mimicry of a single model organism. Instead, selection may favor convergence upon generalized sensory features that exploit preexisting perceptual biases in receivers. In *M. proscarabaeus*, aggregating triungula may therefore represent a form of generalized floral mimicry mediated through sensory exploitation of broadly tuned pollinator recognition mechanisms.

### Larvae May Also Employ Visual Mimicry to Attract Hosts.

The bright orange coloration and shape of the larval aggregates may also visually mimic floral signals, a possibility further supported by the contrasting appearances of *M*. *franciscanus* and closely related *M*. *violaceus* (45) triungula, which are markedly darker (8, 20). As many flowers use ultraviolet (UV) reflectance to attract bees, we hypothesized that *M. proscarabaeus* larvae might employ a similar strategy. However, UV photography of *M*. *proscarabaeus* triungula failed to reveal any significant UV reflectance (*SI Appendix*, Fig. S27). Nevertheless, the observed absence of UV reflectance in larvae does not preclude the visually mediated attraction of bees, suggesting that the conspicuous orange coloration of aggregations may still contribute to host deception. Indeed, increasing evidence indicates that mimics exploit multiple sensory channels simultaneously, enhancing the effectiveness of the deception (46).

### Monoterpenoids Play a Dual Role in Host Attraction and Larval Aggregation.

**Fig. 4.**
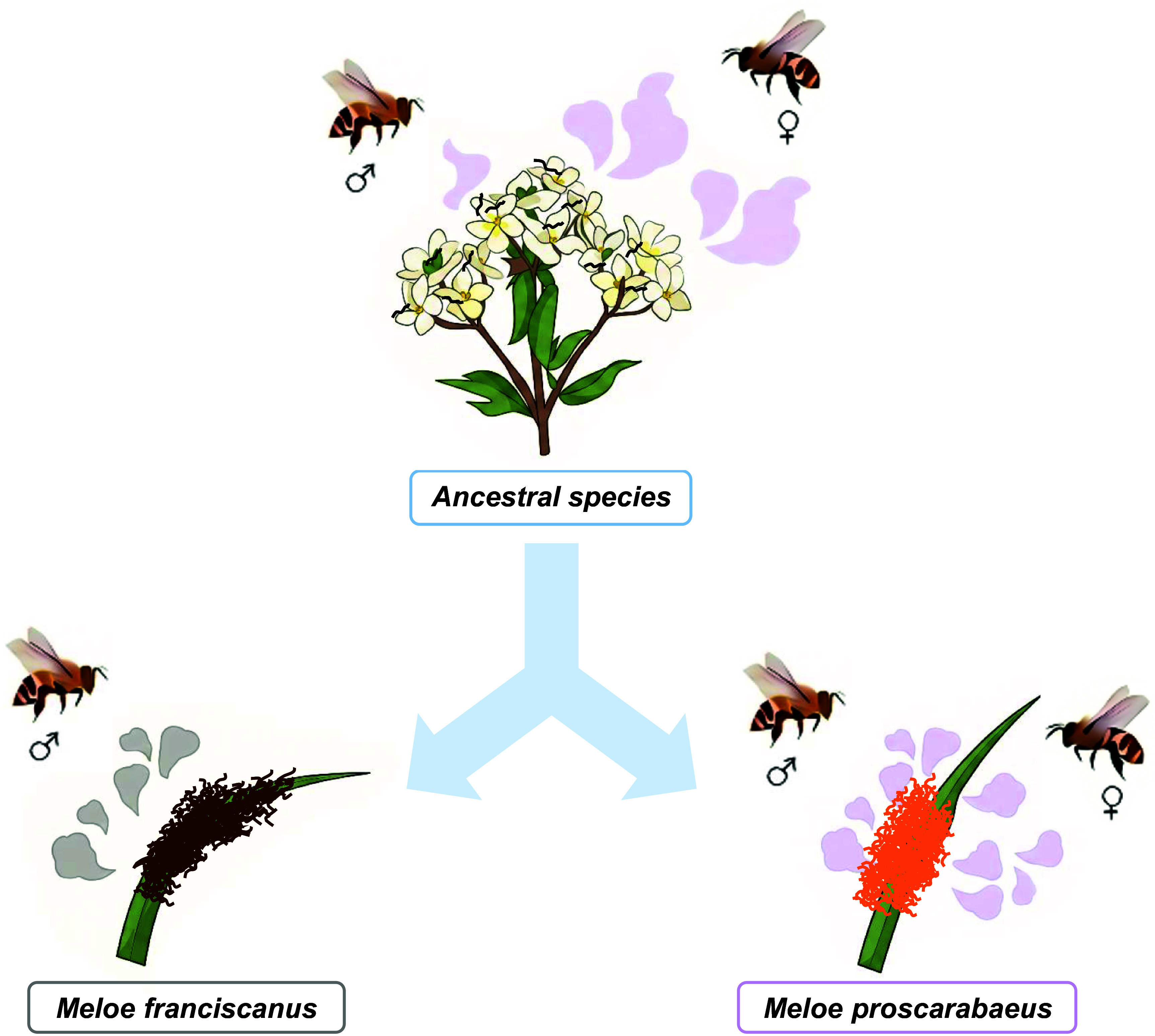
Proposed evolution of floral scent mimicry in *M. proscarabaeus* and sex pheromone mimicry in *M. franciscanus*. Triungula from ancestral species likely relied on exogenous floral volatiles to locate flowers and wait for phoretic bee hosts, or, alternatively, actively searched for bee nests on the soil surface (not illustrated in the figure). In *M. proscarabaeus*, triungula evolved to emit (*S*)-linalool-derived monoterpenoids that initially amplified floral scent and enhanced host attraction. Aggregated larvae subsequently generated stronger floral odor bouquets, reducing their dependence on flowers while concomitantly broadening attraction to a range of phoretic hosts; these compounds additionally function as aggregation pheromones. This strategy may have facilitated exploitation of habitats with sparse floral resources. In *M*. *franciscanus*, it is plausible that larvae emitting long-chain hydrocarbons resembling host bee sex pheromones were more likely to reach suitable bee nests. Selection subsequently favored finely tuned long-chain hydrocarbon blends that mimic local female solitary bee sex pheromones and specifically attract male bee hosts. Created with BioRender.com.

Considering that *M. proscarabaeus* triungula usually aggregate on vegetation but sometimes climb to reach flowers, we hypothesized that larval monoterpenoids may have a dual role: facilitating both host attraction and larval aggregation. Indeed, olfactometric assays showed that triungula were significantly attracted to a synthetic blend of (*S*)-linalool-derived monoterpenoids (**1**–**8**), supporting a dual role for these volatiles as larval aggregation pheromones as well as phoretic vector attractants (*SI Appendix*, Fig. S28 and Table S30). This result suggests an intriguing evolutionary scenario in which triungula likely originally relied on floral volatiles as cues to locate flowers, where they could await pollinating insect hosts (Fig. 4). The emission of such scents by the larvae may have initially merely amplified the natural floral signal. However, ultimately, this also led to the aggregation of triungula, allowing clustered larvae to generate a substantially stronger bouquet than solitary individuals and thereby reduce their dependence on actual flowers. In fact, *M*. *proscarabaeus* larvae typically emerge in the spring near bee nests in areas that have few ground-flowering plants and where early-flowering shrubs and trees, which act as main food source for pollinating bees, are often several hundred meters away. Thus, flower mimicry may have helped *M. proscarabaeus* to occupy new ecological niches. Remarkably, this host-attraction strategy also targets a fundamental behavioral mechanism essential for the survival of pollinating insects, limiting their potential for rapid coevolutionary escape.

### Triungula Biosynthesize Monoterpenoids.

**Fig. 5.**
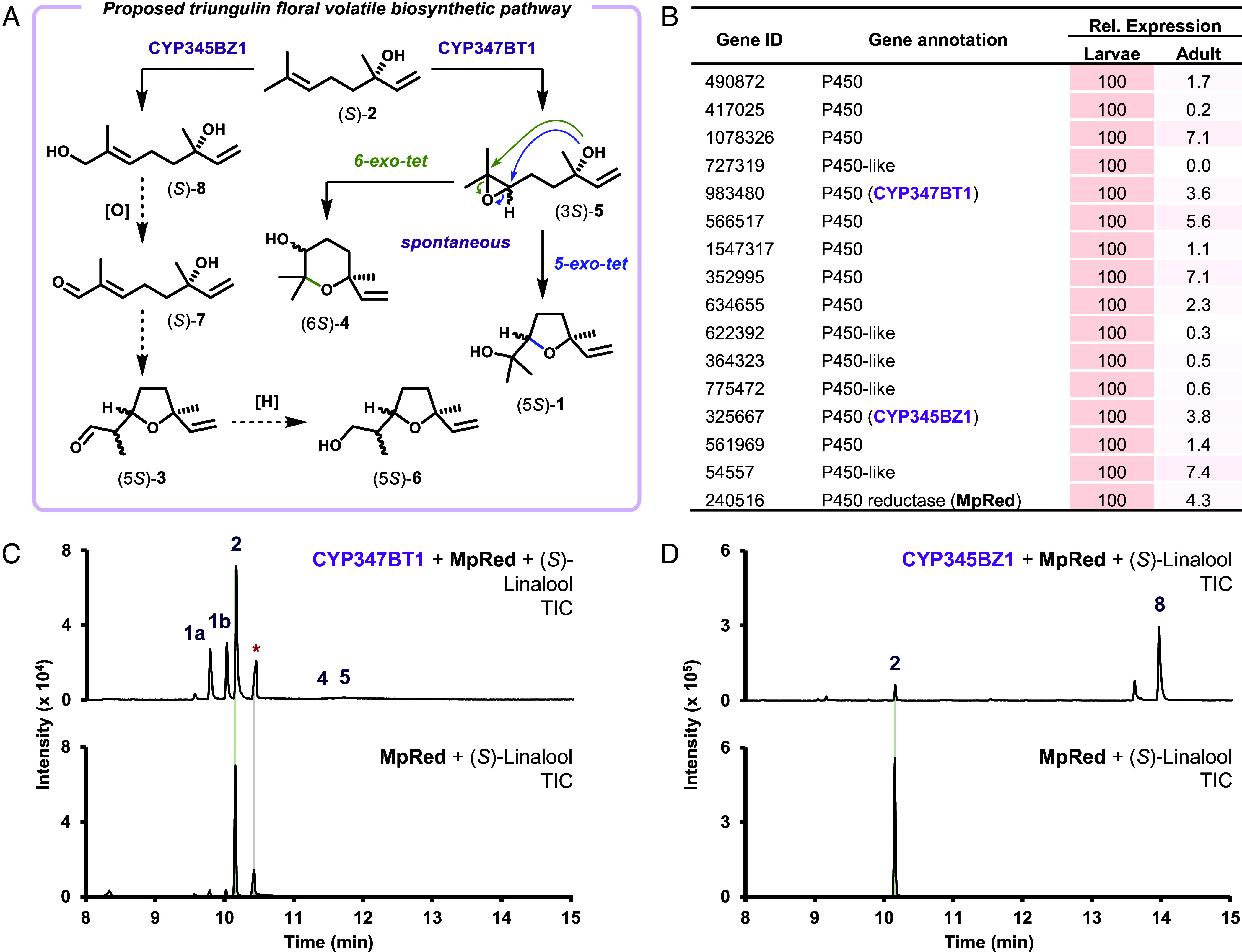
Triungula biosynthesize a range of (*S*)-linalool-derived floral monoterpenoids. (*A*) Proposed biosynthetic pathway of floral-scent volatiles in triungula showing early P450-mediated oxidation of central precursor (*S*)-linalool [(*S*)-**2**] to epoxide (3*S*)-**5** and diol (*S*)-**8**. Spontaneous ring-closure of labile epoxide (3*S*)-**5** subsequently affords linalool oxides (5*S*)-**1** and (6*S*)-**4**, while further oxidation of (*S*)-**8** furnishes intermediate (*S*)-**7** that cyclizes to lilac aldehyde (5*S*)-**3**. Enzymatic reduction of aldehyde (5*S*)-**3** generates alcohol (5*S*)-**6**. (*B*) Differential gene expression analysis identified a cytochrome P450 reductase (MpRed) and P450-encoding transcripts upregulated in larvae relative to adult females. Relative (Rel.) expression values are based on reads per kilobase of transcript per million reads mapped (RPKM) values obtained by RNA-seq (for absolute RPKM values, *SI Appendix*, Table S33). (*C*) Functional characterization of CYP347BT1 in *Saccharomyces* *cerevisiae* expressing either CYP347BT1 and MpRed or MpRed alone in the presence of (*S*)-**2**. Reaction products were analyzed using GC–MS. Peak identification: (*1a* and *1b*) (5*S*)-linalool oxide (furanoid), (*2*) (*S*)-linalool, (*4*) (6*S*)-linalool oxide (pyranoid), (*5*) (3*S*)-linalool-6,7-epoxide. Note: Only traces of (6*S*)-**4** and (3*S*)-**5** were detected. *Background. (*D*) Functional characterization of CYP345BZ1 in *S*. *cerevisiae* expressing either CYP345BZ1 and MpRed or MpRed alone in the presence of (*S*)-**2**. Reaction products were analyzed using GC–MS. Peak identification: (*8*) (*S*)-8-hydroxylinalool.

Following eclosion, larvae of *M*. *proscarabaeus* consistently emit floral-scent monoterpenoids **1**–**8**. However, it remained unclear whether these volatiles were sequestered, maternally transferred, or biosynthesized de novo. However, as larvae do not feed on plants following eclosion (20), the sequestration of floral monoterpenoids seems rather unlikely. To determine if larval monoterpenoids **1**–**8** were maternally derived, we analyzed the volatile profiles of adult females and their eggs, using HS–SPME GC–MS (*SI Appendix*, Figs. S29 and S30). However, apart from the emission of (*S*)-linalool [(*S*)-**2**] by eggs (*SI Appendix*, Fig. S31), we could detect no other floral monoterpenoids in eggs or adults. This result, together with the conserved (*S*) stereochemistry at the linalool C-3 position among compounds **1** and **3**–**8**, led us to hypothesize that triungula biosynthesize these monoterpenoids from (*S*)-**2** (Fig. 5*A*).

As compounds **2** and **5**, and **3**, **7**, and **8** are established intermediates in the biosynthesis of linalool oxides (**1** and **4**) and lilac alcohol (**6**), respectively, in plants (47, 48), we hypothesized that two cytochrome P450 (P450) enzymes generate structural diversity within the triungulin monoterpenoid bouquet by oxidizing (*S*)-linalool [(*S*)-**2**] to (*S*)-**8** and (3*S*)-**5**. To identify candidate biosynthetic genes, we extracted RNA from aggregating triungula that produce these floral volatiles and from adult females that do not, and generated de novo transcriptome assemblies for each. We anticipated that P450 genes responsible for (*S*)-**2** oxidation (i.e., hydroxylation and epoxidation) would be more highly expressed in larvae, and that at least two of the most highly expressed P450 or P450-like transcripts—specifically those encoding proteins with a conserved heme-binding motif—would catalyze these transformations. Consistent with this expectation, differential gene expression analysis identified 15 transcripts annotated as *P450* or *P450-like*, with elevated expression in triungula but not in adult females, along with a putative P450 reductase (*MpRed*) (Fig. 5*B*), which we reasoned would serve as an essential redox partner for the candidate P450 enzymes.

To screen activity, each candidate P450 was individually coexpressed with *MpRed* in *S. cerevisiae* and screened for biosynthetic activity in vivo in the presence of (*S*)-**2**. One candidate, designated CYP347BT1 according to P450 nomenclature, epoxidized (*S*)-**2** to primarily yield linalool oxide (furanoid) stereoisomers, (2*S*, 5*S*)- and (2*R*, 5*S*)-**1**, along with trace amounts of (3*S*)-linalool-6,7-epoxide [(3*S*)-**5**] and linalool oxide (pyranoid) diastereomers (6*S*)-**4** (Fig. 5*C*). Another candidate designated CYP345BZ1, catalyzed the terminal allylic C-H hydroxylation of (*S*)-**2** to produce (*S*)-8-hydroxylinalool [(*S*)-**8**] (Fig. 5*D*). All other P450 candidates (Fig. 5*B*), when coexpressed with *MpRed* in *S. cerevisiae* in the presence of (*S*)-**2**, showed no detectable catalytic activity (*SI Appendix*, Fig. S32). Together, these results strongly suggest that *M*. *proscarabaeus* triungula independently biosynthesize floral-scent monoterpenoids.

Consistent with in vivo assays, HS–SPME and DHV collection extracts of triungula contained relatively low amounts of (3*S*)-**5** and (6*S*)-**4**, compared to linalool oxide (5*S*)-**1** (Fig. 1 *B* and *C*). Furthermore, GC–MS analysis indicated that (3*S*)-**5** undergoes both 5- and 6-exo-tet intramolecular cyclization to form (5*S*)-**1** and (6*S*)-**4**, respectively, during analysis (*SI Appendix*, Fig. S33), congruent with the spontaneous degradation of synthetic (5*S*)-**5** to (5*S*)-**1** and (6*S*)-**4** over time (*SI Appendix*, Fig. S34) (47, 49). The formation of both (2*R*, 5*S*)- and (2*S*, 5*S*)-**1** stereoisomers from (*S*)-**2** in the presence of CYP347BT1 and MpRed further suggests that CYP347BT1-mediated epoxidation proceeds nonstereoselectively, initially generating labile intermediates (3*S*, 6*S*)- and (3*S*, 6*R*)-**5** from (*S*)-**2**.

## Conclusion

Our results reveal a hitherto undescribed form of interkingdom aggressive chemical mimicry in which *M*. *proscarabaeus* triungula collectively emit a complex bouquet of floral-scent monoterpenoids to lure solitary and social bees. Behavioral assays in the laboratory and the field show that these volatiles act as floral-scent mimics—functioning as allomones (50)—rather than sex pheromone analogs, enabling attraction of both male and female solitary bees, promoting successful phoretic dispersal, and coordinating larval aggregation. We identify two P450 enzymes, CYP347BT1 and CYP345BZ1, that generate committed biosynthetic precursors [(3*S*)-**5** and (*S*)-**8**)] responsible for the suite of (*S*)-linalool-derived monoterpenoids (**1**, **3**, **4**, **6**, and **7**). Together, these findings broaden the evolutionary framework of mimicry by demonstrating how an insect can independently produce plant-like signals to manipulate inter- and intraspecific interactions.

## Materials and Methods

### *M.* *Proscarabaeus* Triungulin Cultivation and Collection.

Adult male and female specimens of *M. proscarabaeus* were collected in the field (*SI Appendix*, Fig. S1*A*) during the Spring of 2024 and 2025 in Jena, Thuringia, Germany, with permission from Untere Naturschutzbehörde Jena (permit UNB-AS-BE-AG-2023-01), and during the Spring 2026 in Knoop/Kiel, with permission from the Landesamt für Umwelt des Landes Schleswig-Holstein. Adult beetles were maintained on a diet of fresh *Vicia faba*, *Trifolium pratense*, and *Triticum aestivum*, and housed together in tanks that were kept in a ventilated greenhouse out of direct sunlight (temperature: daytime, *ca*. 10 to 15 °C; night, ≮ 5 °C). Following oviposition (*SI Appendix*, Fig. S1*B*) and subsequent eclosion (*SI Appendix*, Fig. S1*C*), live triungula were collected for analysis when required.

### Triungulin Volatile Collection and Analysis.

Preliminary sampling of *M*. *proscarabaeus* triungulin volatiles was performed using a solid-phase microextraction (SPME) fiber [100 μm poly(dimethylsiloxane) (PDMS), Supelco^®^, Darmstadt, Germany] that had been conditioned at 250 °C for 10 min prior to volatile collection. Briefly, *ca*. 50 live triungula were collected in a 1.5 mL septum-sealed amber glass vial that had previously been heated to 200 °C for 30 min and subsequently cooled (prior to use) and then a preconditioned SPME fiber (heated to 250 °C for 10 min, prior to use) was inserted via the septum of the vial into the headspace (*SI Appendix*, Fig. S2*A*) and left in situ for 3 h, before being directly submitted for GC–MS analysis (vide infra). For dynamic headspace volatile (DHV) collection, live triungula (*ca*. 250) were collected in a 25 mL glass bottle (previously heated to 200 °C for 30 min and subsequently cooled, prior to use). The bottle was connected to an in-house closed loop sampling apparatus (*SI Appendix*, Fig. S2*B*) which comprised a lid fitted with an airtight inlet and outlet containing a removable Porapak™ P cartridge (*SI Appendix*, Fig. S2*C*) (connected via an electronic air circulation pump). Following 3 h of volatile collection the Porapak™ cartridge was removed and flushed with CH_2_Cl_2_ (100 μL × 3). For liquid solvent extractions, approximately 50 triungula were immersed in pentane, hexane, MeOH, or CH_2_Cl_2_. After standing for 1 h at room temperature, extracts were taken off with a glass pipette. All volatile extracts were carefully stored at −20 °C, until required.

### Bee Acquisition and Maintenance.

*O. bicornis* were commercially obtained as individual pupae (Sven Behr Hummelvertrieb; Welle, Lower Saxony, Germany) and *B. terrestris* were acquired as a colony (Katz Biotech AG; Baruth/Mark, Brandenburg, Germany). Specimens of *C. similis* and *A. vaga* were collected in the field (Jena, Thuringia, Germany) with permission from Untere Naturschutzbehörde Jena, and *A. mellifera* were from a local beehive at the Max Planck Institute for Chemical Ecology (MPI–CE; Jena, Thuringia, Germany). Wild-caught bees were stored at 4 °C overnight prior to use in experiments. Animals were individually starved for 4 h in plastic vials containing a bed of moist tissue paper, directly before use in behavioral assays.

### Y-Tube Olfactometer Dual-Choice Assays with Bees.

A Y-tube olfactometer (*SI Appendix*, Fig. S22) was used in dual-choice assays to assess the behavioral responses of all tested bee species (*A. vaga*, *O*. *bicornis*, *C. similis*, *B. terrestris*, and *A. mellifera*) to different odor stimuli. The apparatus consisted of a 3D-printed Y-tube (main arm: length, 12 cm; side-arms: length, 12 cm; internal diameter: 2.2 cm) connected *via* silicon tubing to two 250 mL glass bottles (bottles A1 and A2 and bottles B1 and B2) at the end of each side-arm. When testing bee preference for extracted triungula volatiles obtained by DHV collection, synthetic compounds, or blends thereof, CH_2_Cl_2_ was used as solvent. Treatment samples were filled into 2 mL glass vials and then placed in bottle A1 (*SI Appendix*, Fig. S22*B*). To test extracts and synthetic compounds against more than just neat CH_2_Cl_2_ or nothing, i.e., not just “scent vs. no scent,” a block of eight freshly sprouted wheat plants (*T. aestivum*) were placed in bottle A1 of each side-arm to create a standardized volatile background. Vials containing solutions of synthetic compounds, DHV collection extracts, or neat CH_2_Cl_2_ were placed in a holder that positioned it in the center of the glass bottle above the wheat. For choice assays with living triungula, animals were placed directly on the wheat in bottle A1. To maintain humidity in the testing arena and thus preserve volatile perception, in-house purified compressed air was first passed through bottle A2 or B2 which were filled with distilled water, prior to entering bottles A1 or B1 (containing wheat and a solution of synthetic compound in CH_2_Cl_2_ at a concentration of 1 mg/mL or neat CH_2_Cl_2_), respectively. The mixture of synthetic (*S*)-linalool-derived monoterpenoids was freshly prepared by blending individual compounds in a ratio corresponding to the HS–SPME GC–MS larval profile obtained after 3 h of collection. The charcoal-filtered and humidified air was pumped through each side-arm of the Y-tube at a constant flow rate of 150 mL/min and was regulated by flow meters to ensure equal airflow. At the base of the main arm, air was extracted from the system at a rate of 420 mL/min, to prevent saturation of volatiles in the Y-tube and create a continuous air flow through both side-arms. Before each trial, the Y-tube was cleaned with 70% (v/v) EtOH and oven-dried at 60 °C to remove residual odors. The experiment was conducted under controlled environmental conditions (23 ± 1 °C, 65 ± 5% RH). A Planon Plus 30 × 60 cm light panel (22 W; 4,000 K; 1,600 lm) was used as a light source over the enclosed Y-tube to create a stable light intensity of 100 μmol/(m^2^s) on the surface of the arena. An individual insect was gently released at the base of the main arm and allowed up to 5 min to make a choice by walking to the end of one of the two side-arms. Individuals that did not make a choice within the allotted time were recorded as “no response” and excluded from statistical analyses. To avoid positional bias, the odor sources were alternated between the left and right arms after every insect, and the Y-tube apparatus was replaced or cleaned regularly. Between two test subjects and the exchange of the odor sources, air was allowed to flow through the setup for 5 min. During experiments, the arena was monitored from above using a webcam that transmitted the image to a laptop so as not to influence the animals’ decision. Each treatment was tested with 18 to 22 replicates, using a new bee for each repetition. The proportion of bees that chose each side-arm was analyzed using a chi-square (χ^2^) test of independence (Microsoft^®^ Excel^®^ LTSC MSO 16.0) with a significance level of *P* < 0.05.

### Observational Field Behavioral Assays.

For all field experiments, two artificial flowers consisting of colorless cotton swabs and were placed 40 to 50 cm apart in direct sunlight in a meadow near a known *A. vaga* nesting site in Wesenberg, Mecklenburg-Vorpommern, Germany. For each experimental replicate, one artificial flower was treated with 100 μL of monoterpenoid-containing DHV collection extract in CH_2_Cl_2_, whereas the second flower was dosed with 100 μL of neat CH_2_Cl_2_ as a solvent control. The number of visits by *A. vaga* to each artificial flower was recorded over a 15 min observation period. The same methodology was used to evaluate the long-chain hydrocarbon extract, except that the treatment consisted of a pentane extract taken from a single aggregation of triungula and the solvent control was neat pentane. All experimental replicates (*n* = 14) were performed with the positions of treatment and control flowers reversed, i.e., for each replicate control and treatment flowers were switched between an easterly and westerly orientation. Field experiments were conducted between 10:15 and 18:00 h, at temperatures ranging from 13 to 17 °C. The number of bees visiting treatment and control flowers were compared using a Wilcoxon signed-rank test (Microsoft^®^ Excel^®^ LTSC MSO 16.0), with significance assessed at *P* < 0.05.

### Visible (VIS) and Ultraviolet (UV) Photography.

A single aggregation of *M. proscarabaeus* larvae were gathered on a wooden stick and images were subsequently captured outdoors in full sunlight, as described previously (51).

### Triungulin Olfactometric Choice Assays.

An in-house setup, consisting of two equal-sized wooden toothpicks fixed to a sheet of circular filter paper on an inverted glass Petri dish positioned within a larger Petri dish containing water (*SI Appendix*, Fig. S28) was used to assess triungulin preference for a synthetic blend of (*S*)-linalool-derived monoterpenoids. To remove light bias, all experiments were performed in a dark room under an LED light panel. Immediately before starting an experiment, one toothpick was dosed with 50 μL of the synthetic (*S*)-linalool-derived monoterpenoid blend (prepared as described previously) dissolved in CH_2_Cl_2_ and 50 μL of neat CH_2_Cl_2_ was applied to the other. Approximately 20 triungula were directly released mid-way between these two toothpicks (t_0_). The absolute number of triungula present on each toothpick was then recorded at 5 min intervals for 30 min, following release. Absolute numbers of triungula on toothpicks at each time point were recorded and analyzed using a paired *t* test: paired two sample for means (Microsoft^®^ Excel^®^ LTSC MSO 16.0) to determine if there was a statistically significant preference for the monoterpenoid blend (*SI Appendix*, Table S30).

### Transcriptome Sequencing and Gene Identification.

Female *M. proscarabaeus* beetles (biological replicates, *n* = 3) were dissected in PBS into fat body, gut, reproductive organs, and head, and immediately transferred to ice-cold Trizol (Direct-zol, Zymo Research). Samples were homogenized in ceramic bead tubes using a TissueLyser LT (Qiagen) and total RNA was extracted from the different tissues using the Direct-zol RNA Miniprep kit (Zymo Research) according to the manufacturer’s instructions. The concentration and integrity of the RNA were determined using an N60 nanophotometer (Implen) and a TapeStation System (Agilent), respectively. After RNA-seq library preparation (poly-A enrichment), short-read sequencing was performed on an Illumina HiSeq 2500 sequencer (Max Planck Genome Centre, Cologne, Germany) with ~30 Mio reads per library, 150 bp per read, paired end. In addition, RNA from the different tissues were pooled and subjected to PacBio long-read RNA sequencing on the Sequel II system (Max Planck Genome Centre), resulting in ~93 Gb sequence data. Total RNA from *M. proscarabaeus* larvae (three samples, each containing approximately 50 larvae) was extracted using the RNeasy^®^ Plant Mini Kit (Qiagen) according to the manufacturer’s instructions, and was sequenced by using Illumina technology (poly-A enrichment, NovaSeq X Plus (Novogene, Munich, Germany), 150 bp per read, paired end, 60 Mio raw reads per sample). Aliquots of the individual larval RNA extractions were pooled and PacBio long-read RNA sequencing was performed on the Revio system (Max Planck Genome Centre), resulting in ~950 Gb sequence data. For generating a reference transcriptome as mapping template, the PacBio sequence reads from female beetles and larvae were combined and processed using the IsoSeq pipeline including the removal of cDNA primer, demultiplexing, polyA-tail trimming and clustering. Trimming of the Illumina reads and mapping to the PacBio reference transcriptome were performed with the software CLC Genomics Workbench (Qiagen Bioinformatics, mapping parameters: mismatch cost, 2; insertion cost, 3; deletion cost, 3; length fraction, 0.8; similarity fraction, 0.95; maximum number of hits, 5). Note, the reads from the four different tissues of *M. proscarabaeus* female beetles were combined prior mapping. To identify cytochrome P450 genes potentially involved in volatile terpenoid biosynthesis, we first selected genes annotated as *P450* or *P450-like*. We further refined this list by selecting genes expressed at least 10-fold more in the larvae than in female beetles, with a minimum expression threshold of 10 RPKM. This approach identified 15 potential P450 candidate genes.

### Gene Cloning.

Complementary DNA (cDNA) was prepared from total RNA of *M. proscarabaeus* triungula, using the SuperScript™ IV VILO™ Master Mix kit (Thermo Fisher Scientific) following the manufacturer’s instructions. Coding sequences were amplified with the Platinum™ SuperFi II DNA polymerase kit (Thermo Fisher Scientific) using *M. proscarabaeus* triungulin cDNA as template and gene-specific primers listed in *SI Appendix*, Table S31. Amplified sequences are described in *SI Appendix*, Table S32. Constructs for heterologous expression in *S. cerevisiae* were prepared by cloning *CYP345BZ1* or *CYP347BT1* in combination with *MpRed* as sticky-end fragments (*MpRed*) or using In-Fusion^®^ (*CYP345BZ1* and *CYP347BT1*; TaKaRa) into the same pESC-Leu-2d vector employing two different cloning sites with (52). Inserted sequences were confirmed by Sanger sequencing.

### Heterologous Expression of P450 Gene Candidates in *S. Cerevisiae* and In Vivo Enzyme Assays.

Following a previously described procedure for heterologous expression in yeast (53), constructs were transformed into the INVSc1 (Thermo Fisher Scientific) *S. cerevisiae* strain using the *S.c.* EasyComp™ Transformation Kit (Invitrogen) according to the manufacturer’s instructions. Subsequently, 30 mL Yeast Synthetic Drop-out Medium Supplement without leucine (Sigma Aldrich Y1376-20 g; containing 70 mg/L adenine and 20 g/L D-glucose) was inoculated with single yeast colonies and grown overnight at 30 °C and 200 rpm. For main cultures, 100 mL yeast peptone glucose agar (YPGA) (Glc) full medium (10 g/L yeast extract, 20 g/L bactopeptone, 74 mg/L adenine hemisulfate, 20 g/L D-glucose) was inoculated with one-unit OD600 of the overnight cultures and incubated under the same conditions for 30 to 35 h. After centrifugation (5,000 × g, 16 °C, 5 min), expression was induced by resuspension of the cells in 100 mL YPGA (Gal) medium (see above, but including 20 g/L D-galactose instead of D-glucose) and grown for another 2 h at 30 °C and 200 rpm. For in vivo enzyme assays, a 600 μL aliquot of culture was transferred to a septum-sealed 4 mL glass vial and then treated with 6 μL of a 1 mg/mL solution of (*S*)-linalool in MeOH. After approximately 24 h at 30 °C and 200 rpm, the culture was overlaid with EtOAc (200 μL), and vortexed for 1 min. Following phase separation, the organic layer was carefully removed and submitted for GC–MS analysis using separation method A.

## Supplementary Material

Appendix 01 (PDF)

## Data Availability

RNAseq raw data; Genes data have been deposited in EDMOND; NCBI [https://doi.org/10.17617/3.7TXB4J (54); MpRed (PX569140) (55), CYP347BT1 (PX569141) (56, and CYP345BZ1 (PX569142) (57)]. All other data are included in the manuscript and/or *SI Appendix*.
