# Supplementary material for: The floral illusion: A parasitic beetle mimics the scent of flowers to attract bees
Source: Proc Natl Acad Sci U S A. 2026 Jul 28;123(31):e2602521123. doi: 10.1073/pnas.2602521123 (PMC13438595; doi:10.1073/pnas.2602521123)
Supplement: Supplementary file 1 [file pnas.2602521123.sapp.pdf]

Supplementary Information

The floral illusion: A parasitic beetle mimics the scent of flowers to attract bees

Ryan M. Alam, Danny Kessler, Heiko Vogel, Katrin Luck, Anja David, Maritta Kunert, Gunnar Brehm, Martin Kaltenpoth, Sarah E. O'Connor, Tobias G. Köllner

Correspondence to: Sarah E. O'Connor, oconnor@ice.mpg.de; Tobias G. Köllner, koellner@ice.mpg.de

This PDF file includes:

Supplementary Materials and Methods
Supplementary Figs. 1–34
Supplementary Tables 1–33
Supplementary References (58–69)

Supplementary Materials and Methods

Gas chromatography-mass spectrometry (GC-MS)

Gas chromatography-mass spectrometry (GC-MS) analyses were performed using an Agilent 8890 Series gas chromatograph coupled with an Agilent 5977B single quadrupole mass selective detector (MSD) (Agilent Technologies, CA 95051, United States). Unless stated otherwise, samples were prepared using CH_2Cl_2 as sample solvent to give a final concentration of *ca*. 10 µg/mL. Chromatographic analyses were performed using helium as the carrier gas applied at a constant flow rate with an injection volume of 1 µL in splitless mode and MS data acquisition was carried out in EI mode using an electron ionization energy of 70 eV. HS-SPME analyses were carried out by inserting the SPME fiber directly into the injection port of the GC.

Chromatographic separations were performed using the following method:

Separation method: A

Chromatographic separations were carried out using a Zebron ZB-5™ column (5% phenyl, 95% dimethylpolysiloxane; 30 m length, 0.25 mm i.d., 0.25 µm d_f, 10 m pre-column, Phenomenex, Aschaffenburg, Germany). Carrier gas was applied at a constant flow rate of 1 mL/min. The inlet and transfer line temperatures were 250 °C and 280 °C, respectively. For chromatographic separation, an initial column oven temperature of 40 °C was maintained for 2 min and then increased by 10 °C/min to 280 °C and then held for 2 min. Solvent delay was set to 5 min. The ion source temperature was 230 °C. MS data acquisition was carried out in scan mode (mass range, 50–450 m/z).

GC-MS analyses employing chiral stationary phases were carried out using the following separation methods:

Separation method: B

Employing a β-DEX™ 225 column (25% 2,3-di-*O*-acetyl-6-*O*-TBDMS-β-cyclodextrin in SPB-20 poly(20% phenyl/80% dimethylsiloxane); 30 m length, 0.25 mm i.d., 0.25 d_f; Supelco), both the inlet and transfer line temperatures were set to 220 °C. Carrier gas was applied at a constant flow rate of 1.2 mL/min. For chromatographic separation, an initial column temperature of 40 °C was held for 2 min and then increased by 2 °C/min to 200 °C. After holding at 200 °C for 5 min, the temperature was then decreased by 7 °C/min to 40 °C. The solvent delay was set to 12 min and MS data acquisition was carried out in scan mode (mass range, 50–500 m/z).

Separation method: C

Using a Hydrodex β-6TBDM column (Heptakis(2,3-di-*O*-methyl-6-*O*-tert-butyldimethylsilyl)-β-cyclodextrin; 25 m length, 0.25 mm i.d.; Macherey-Nagel, Düren, Germany), both the inlet and transfer line temperatures were set to 220 °C. The carrier gas was applied at a constant flow rate of 1.5 mL/min and the solvent delay was set to 15 min. For chromatographic separation, the column temperature was initially held at 60 °C for 2 min and then increased by 2 °C/min to 120 °C. After holding the temperature at 120 °C for 10 min, the column temperature was increased by 5 °C/min to 180 °C, held for 5 min, and then decreased by 10 °C/min to 60 °C. MS data acquisition was carried out in scan mode (mass range, 50–350 m/z).

Separation method: D

Employing a β-DEX™ 110 column (10% permethylated β-cyclodextrin in SPB-35 poly(35% diphenyl/65% dimethylsiloxane; 30 m length, 0.25 mm i.d., 0.25 d_f, Supelco), both the inlet and transfer line temperatures were set to 220 °C with a constant carrier gas flow rate of 1.2 mL/min. Chromatographic separation was performed using an initial column temperature of 50 °C that was held for 2 min and then increased by 2 °C/min to 150 °C. After holding at 150 °C for 10 min, the temperature was further increased by 4 °C/min to 180 °C, held for 5 min, and then decreased by 7 °C/min to 50 °C. The solvent delay was set to 15 min and MS data acquisition was carried out in scan mode (mass range, 50–350 m/z).

Separation method: E

Using a β-DEX™ 225 column (25% 2,3-di-*O*-acetyl-6-*O*-TBDMS-β-cyclodextrin in SPB-20 poly(20% phenyl/80% dimethylsiloxane); 30 m length, 0.25 mm i.d., 0.25 d_f; Supelco), both the inlet and transfer line temperatures were set to 220 °C. Carrier gas was applied at a constant flow rate of 1.2 mL/min. For chromatographic separation, an initial column temperature of 40 °C was held for 2 min and then increased by 8 °C/min to 120 °C. After holding at 120 °C for 60 min, the temperature was increased by 2 °C/min to 160 °C and then further increased by 8 °C/min to 200 °C, held for 5 min, and subsequently decreased by 8 °C/min to 40 °C. The solvent delay was set to 12 min and MS data acquisition was carried out in scan mode (mass range, 50–500 m/z).

Separation method: F

Utilizing a β-DEX™ 225 column (25% 2,3-di-*O*-acetyl-6-*O*-TBDMS-β-cyclodextrin in SPB-20 poly(20% phenyl/80% dimethylsiloxane); 30 m length, 0.25 mm i.d., 0.25 d_f; Supelco), both the inlet and transfer line temperatures were set to 220 °C. Carrier gas was applied at a constant flow

rate of 1.2 mL/min. For chromatographic separation, an initial column temperature of 40 °C was held for 2 min and then increased by 8 °C/min to 130 °C. After holding at 130 °C for 90 min, the temperature was increased by 2 °C/min to 180 °C and then further increased by 8 °C/min to 200 °C, held for 5 min, and subsequently decreased by 8 °C/min to 40 °C. The solvent delay was set to 12 min and MS data acquisition was carried out in scan mode (mass range, 50–500 m/z).

Chemical synthesis of monoterpenoid volatiles

General note

All reagents were obtained from commercial sources and used without any further purification, unless otherwise specified. Powdered 4 Å molecular sieves were dried (200 °C for 48 h), prior to use. Room temperature ranged between 18–22 °C. Thin layer chromatography (TLC) was carried out on aluminum-backed silica gel 60 F_{254} plates (Merck) that were visualized with Hanessian's stain and/or using $UV_{254\ nm}$ light detection. Flash column chromatography was performed using Merck silica gel 60 (particle size 0.040–0.063 mm, density 0.8 g/cm^3).

Nuclear magnetic resonance (NMR) spectroscopy

Nuclear magnetic resonance (NMR) spectra were recorded on a Bruker Avance® III HD 400 MHz spectrometer (Bruker Biospin GmbH, Rheinstetten, Germany) at room temperature (*ca*. 293 K), using $CDCl_3$ as sample solvent. Chemical shift values (δ_H) are reported in parts per million (ppm) relative to residual solvent (δ_H 7.26 [C*H*Cl_3]) and coupling constants (*J*) are expressed in Hertz (Hz), in the following format; chemical shift value (multiplicity, coupling constant, integration). ^{1}H NMR spectral data are described, using the following abbreviations; brs (broad singlet), s (singlet), t (triplet), q (quartet), dd (doublet of doublets), ddd (doublet of doublets of doublets), appd (apparent doublet), and m (multiplet).

Synthesis of (*S*)-linalool ((*S*)-2)

Following a reported route [58, 59], (*S*)-**2** was prepared over three steps from geraniol (**S1**).

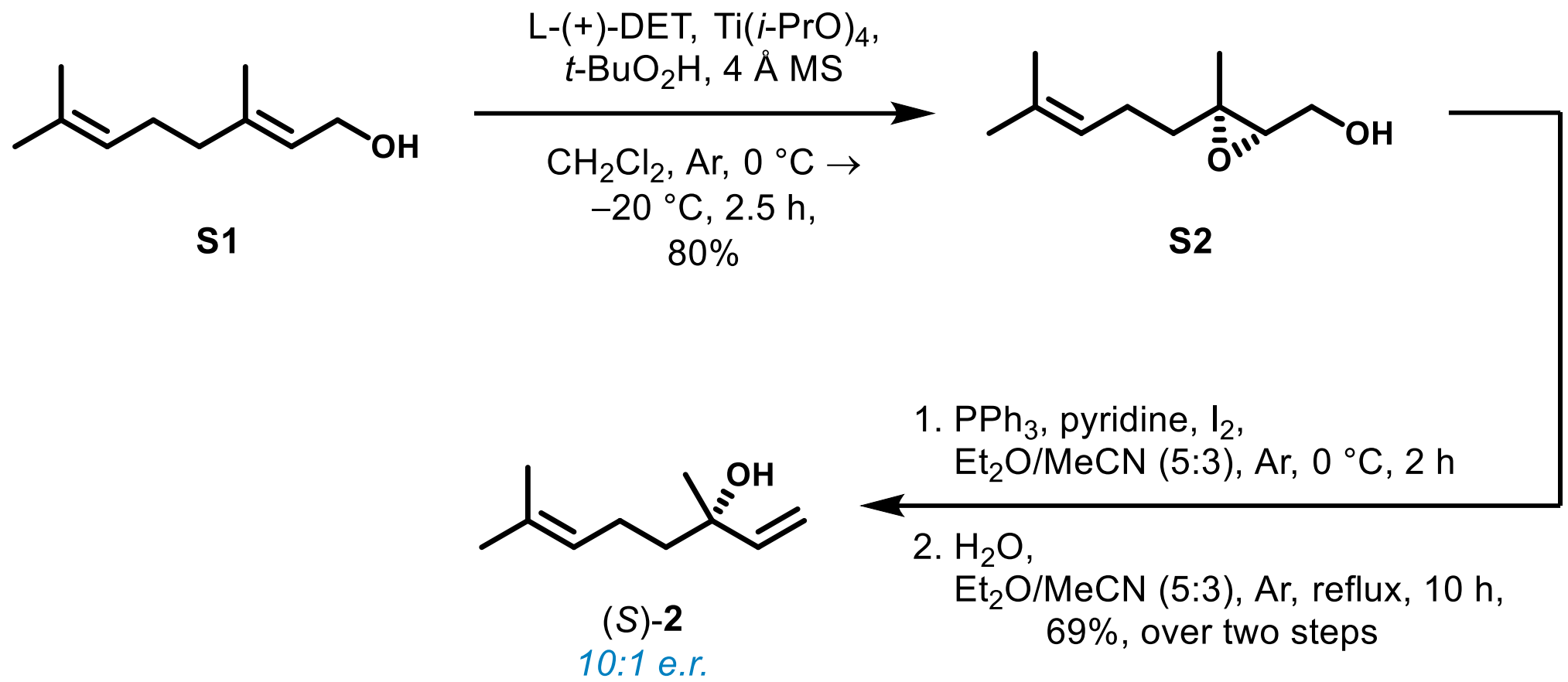

[(2*S*,3*S*)-3-Methyl-3-(4-methylpent-3-en-1-yl)oxiran-2-yl]methanol (**S2**)

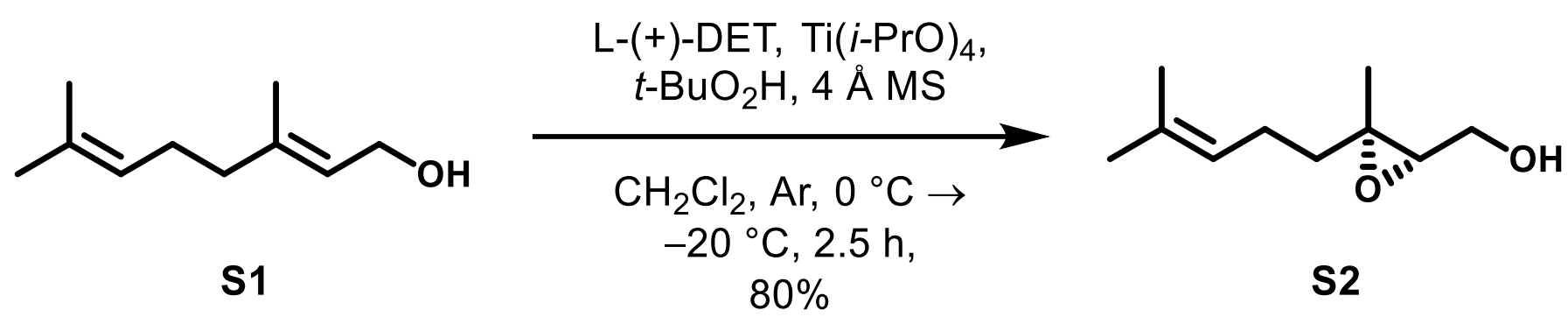

To a dry 250 mL two-neck round bottom flask fitted with a dropping funnel under an Ar atmosphere was added powdered 4 Å molecular sieves (1.8 g) and anhydrous CH_2Cl_2 (50 mL). Upon cooling to −20 °C, the suspension was sequentially charged with L-(+)-diethyl tartrate (1.66 mL, 9.72 mmol), Ti(*i*-PrO)$_4$ (1.92 mL, 6.48 mmol), and *t*-butyl hydroperoxide (5.5 M in *n*-decane, 17.68 mL, 97.25 mmol). After stirring at −20 °C for 30 min, to the mixture was then slowly added geraniol (**S1**, 3.16 mL, 18.21 mmol) *via* dropping funnel. Reaction monitoring using TLC (1:3, EtOAc/ petroleum ether [b.p. 40–60 °C]) confirmed complete consumption of allylic alcohol **S1**, after stirring at −20 °C for a further 2.5 h. The reaction mass was gradually warmed to 0 °C and then charged with 30% (w/v) NaOH in sat. aq. $NaHCO_3$ (20 mL). After stirring at 0 °C for 30 min, the mixture was warmed to room temperature and further diluted with H_2O (20 mL). The resulting biphasic suspension was passed through a short pad of Celite®. Following phase separation, the aqueous layer was repeatedly extracted with CH_2Cl_2 (20 mL x 3). The combined organic layers were dried over anhydrous $MgSO_4$ and carefully concentrated under reduced pressure gave a residue that was subsequently subjected to short path vacuum distillation to afford an oil, which was then purified by gradient flash column chromatography (0–25% EtOAc in petroleum ether [b.p. 40–60 °C]) to yield title compound **S2** as a colorless oil (2.480 g, 80%): R_f 0.40 (1:3, EtOAc/petroleum ether [b.p. 40–60 °C]); ^{1}H NMR ($CDCl_3$, 400 MHz) δ 5.09–5.06 (m, 1H), 3.83–

3.79 (m, 1H), 3.70–3.65 (m, 1H), 2.97 (dd, J = 6.7, 4.3 Hz, 1H), 2.08 (q, J = 7.7 Hz, 2H), 1.87 (brs, 1H), 1.71–1.63 (overlapping m, 1H), 1.68 (s, 3H), 1.60 (s, 3H), 1.50–1.43 (m, 1H), 1.29 (s, 3H). Physical and spectral data agreed with those reported previously [59].

(*S*)-Linalool ((*S*)-**2**)

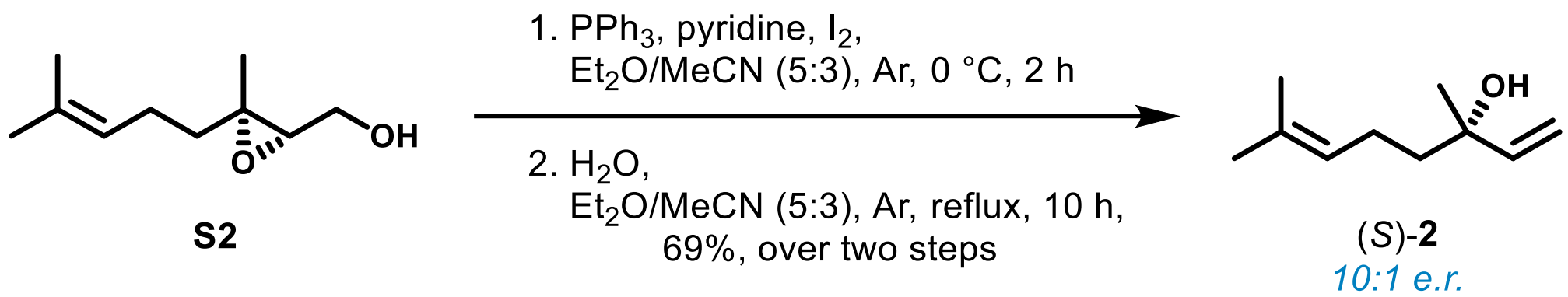

To a dry 250 mL round bottom flask, containing an ice-cold solution of **S2** (2.465 g, 14.48 mmol) in a mixture of anhydrous Et_2O (50 mL) and MeCN (30 mL) under an Ar atmosphere, was sequentially added triphenylphosphine (11.394 g, 43.44 mmol), anhydrous pyridine (4.67 mL, 57.92 mmol), and I_2 (5.513 g, 21.72 mmol). After stirring at 0 °C for 2 h, the reaction mass was charged with H_2O (0.26 mL) and heated to reflux (45 °C, oil bath) for 10 h. Upon cooling to room temperature, the resulting mixture was treated with 20% (w/v) aq. $Na_2S_2O_3$ (8 mL) and sat. aq. $NaHCO_3$ (8 mL). Following separation, the aqueous layer was extracted with Et_2O (10 mL x 3) and the combined organic phases were washed with 1 M aq. HCl (10 mL), sat. aq. $NaHCO_3$ (10 mL), H_2O (10 mL), and brine (10 mL). Careful concentration of the organic phase gave an oily residue that was further purified by gradient flash column chromatography (0–10% Et_2O in pentane) to afford title compound (*S*)-**2** (10:1 e.r.) as a colorless oil (1.541 g, 69% [over two steps from **S2**]): R_f 0.50 (1:4, Et_2O/pentane); ^{1}H NMR ($CDCl_3$, 400 MHz) δ 5.91 (dd, J = 17.3, 10.8 Hz, 1H), 5.21 (dd, J = 17.3, 1.2 Hz, 1H), 5.14–5.10 (m, 1H), 5.06 (dd, J = 10.8, 1.2 Hz, 1H), 2.10 (m, 2H), 1.68 (s, 3H), 1.60 (overlapping s, 3H), 1.60–1.56 (overlapping m, 2H), 1.28 (s, 3H); GC-MS (EI) *m*/*z* (%) 71 (100), 93 (85), 55 (50), 69 (42), 80 (35), 121 (27), 83 (19), 67 (19), 92 (17), 79 (13). Physical and spectral data agreed with those reported previously [60].

GC-MS extracted ion chromatogram (EIC) data for (**A**) synthetic (*S*)-**2** (10:1 *e.r.*;), (**B**) (*R*)-**2**, and (**C**) *rac*-**2**. Separation method: B.

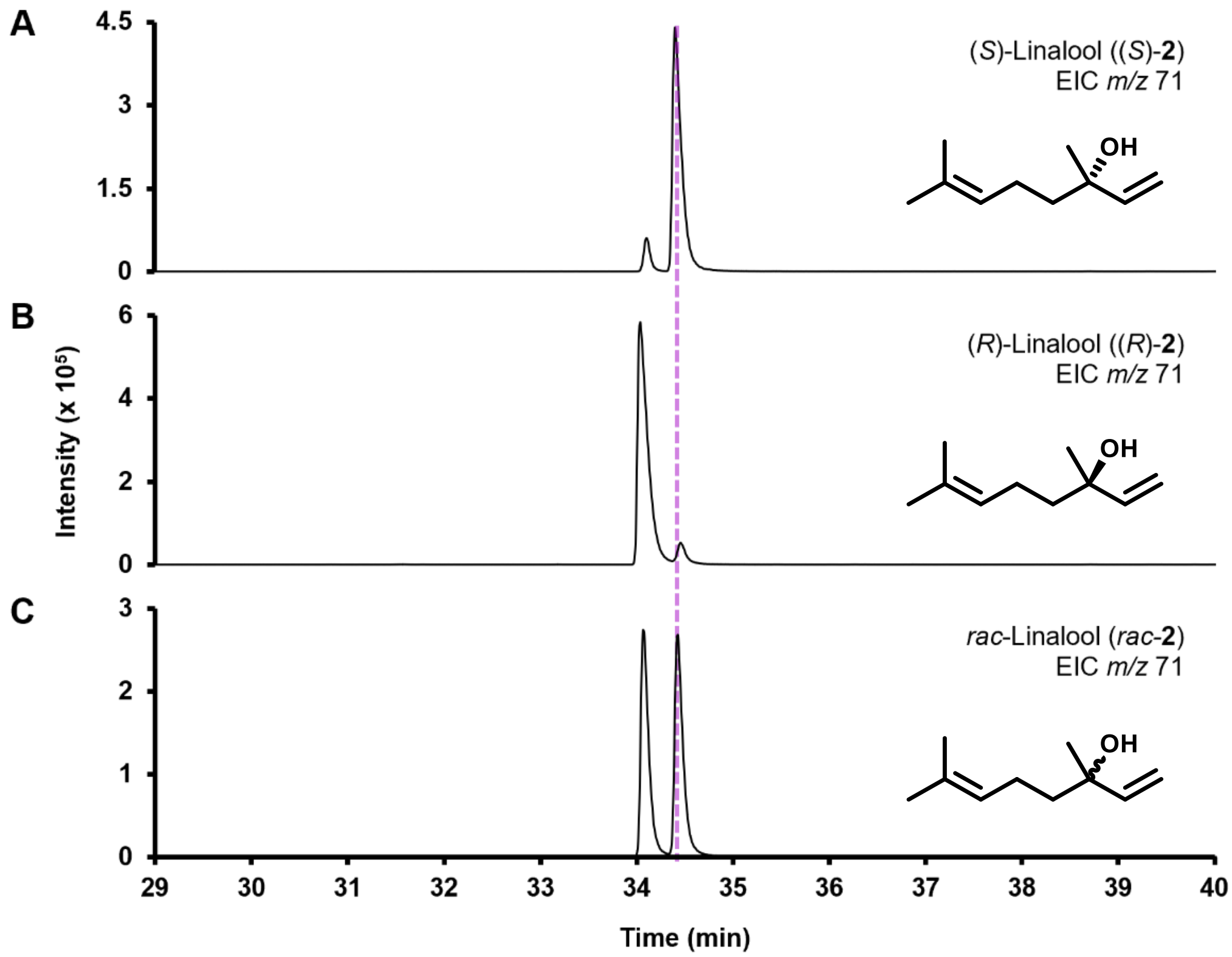

Synthesis of linalool oxide (furanoid) (1) and (pyranoid) (4)

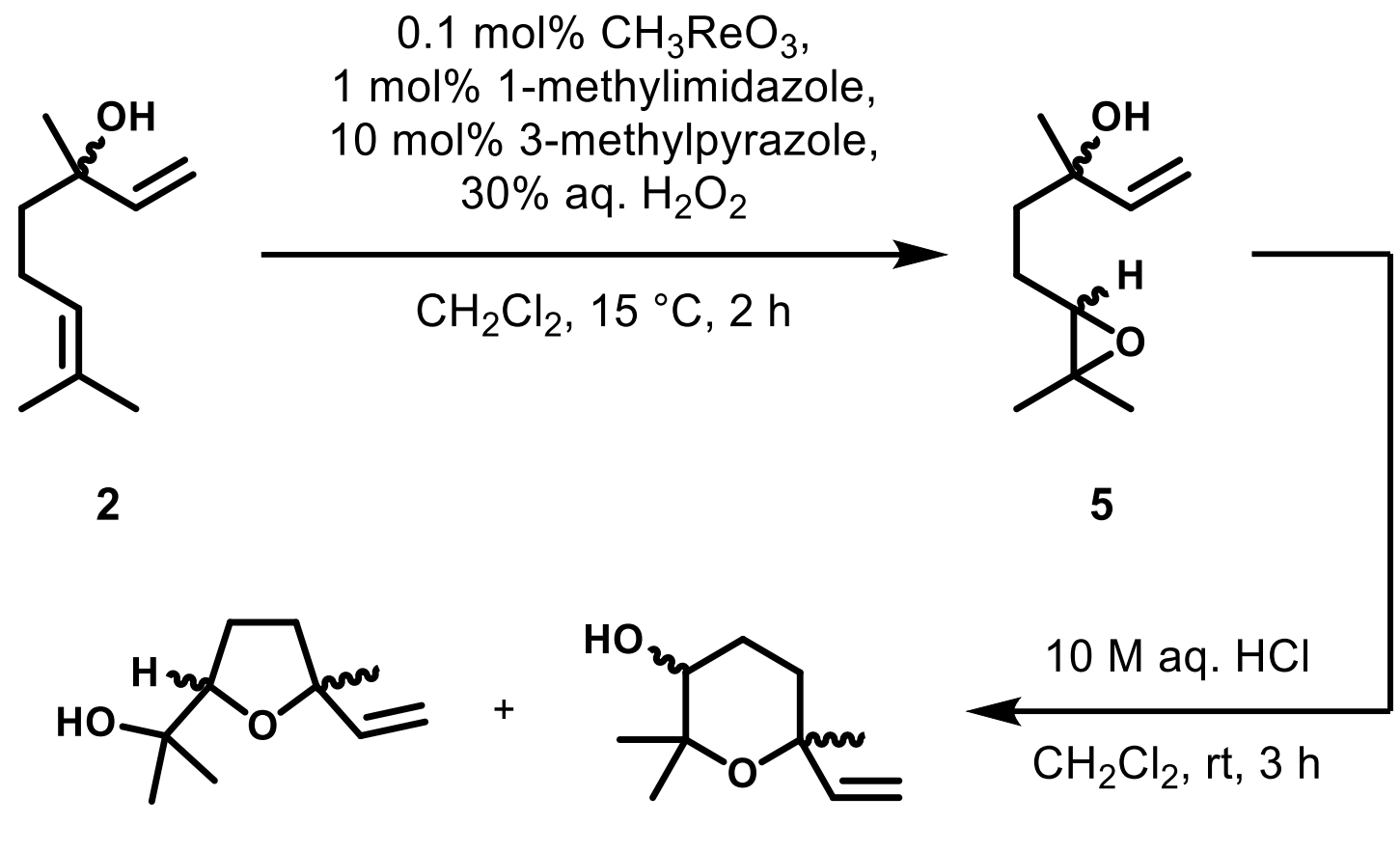

Linalool-6,7-epoxide (**5**, (3*S*)-**5**, and (3*R*)-**5**)

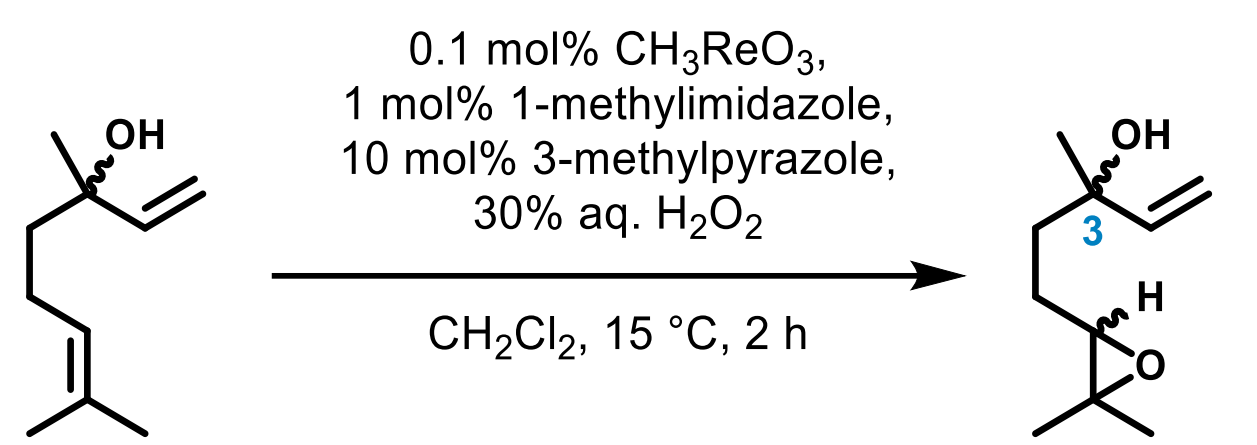

Briefly following a previously reported procedure [61], to a 10 mL round bottom flask containing *rac*-linalool (**2**, 1.80 mL, 10 mmol) in CH_2Cl_2 (5 mL) cooled to 15 °C, was added 3-methyl-1*H*-pyrazole (80 µL, 1 mmol), 1-methyl-1*H*-imidazole (8 µL, 0.1 mmol), and methyltrioxorhenium (2.5 mg, 0.01 mmol). Following the addition of 30% (w/w) aq. H_2O_2 (1.2 mL, 10.6 mmol), the resulting biphasic mixture was vigorously stirred for 2 h at 15 °C. The reaction mass was charged with brine (10 mL) and extracted with CH_2Cl_2 (5 mL x 3). The combined organic layers were washed with sat. aq. $Na_2S_2O_3$ (5 mL) and then dried over anhydrous Na_2SO_4. Careful concentration *in vacuo* gave title compound **5** as a colorless oil (1.701 g, quant.): ^1H NMR ($CDCl_3$, 400 MHz) mixture of diastereoisomers* δ 5.93–5.84 (m, 1H), 5.24–5.19 (m, 1H), 5.08–5.04 (m, 1H), 2.74–2.71 (m, 1H), 1.79–1.49 (m, 4H), 1.30–1.29 (m, 3H), 1.26 (s, 3H). Physical and spectral data agreed with those reported previously [61]. *Both diastereoisomers of **5** are described.

Note: Following this procedure, (3*R*)-**5** and (3*S*)-**5** were analogously prepared from (*R*)- and (*S*)-linalool, respectively.

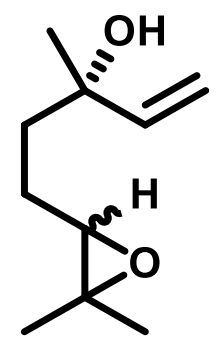

(3*S*)-**5**, quant.

(3*S*)-Linalool-6,7-epoxide ((3*S*)-**5**) was isolated as a colorless oil (1.701 g [quant.] from 1.543 g [10 mmol] of (*S*)-**2**): GC-MS (EI) *m*/*z* (%) *2*) 71 (100), 68 (77), 59 (75), 67 (54), 72 (51), 55 (38), 97 (36), 79 (30), 69 (28), 85 (25), *4*) 71 (100), 68 (78), 59 (75), 67 (54), 72 (49), 97 (37), 55 (36), 79 (31), 69 (28), 85 (25). Physical and spectral data agreed with those reported previously [61].

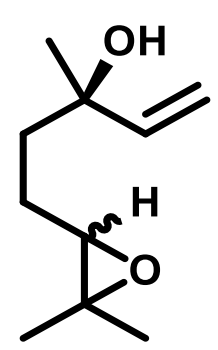

(3*R*)-**5**, quant.

(3*R*)-Linalool-6,7-epoxide ((3*R*)-**5**) was isolated as a colorless oil (1.699 g [quant.] from 1.543 g [10 mmol] of (*R*)-**2**): Physical and spectral data agreed with those reported previously [61].

GC-MS EIC data for (**A**) synthetic linalool-6,7-epoxide **5** (peaks 1–4), (**B**) (3*S*)-**5** (peaks 2 and 4), and (**C**) (3*R*)-**5** (peaks 1 and 3). Separation method: C. *Mixture of stereoisomers.

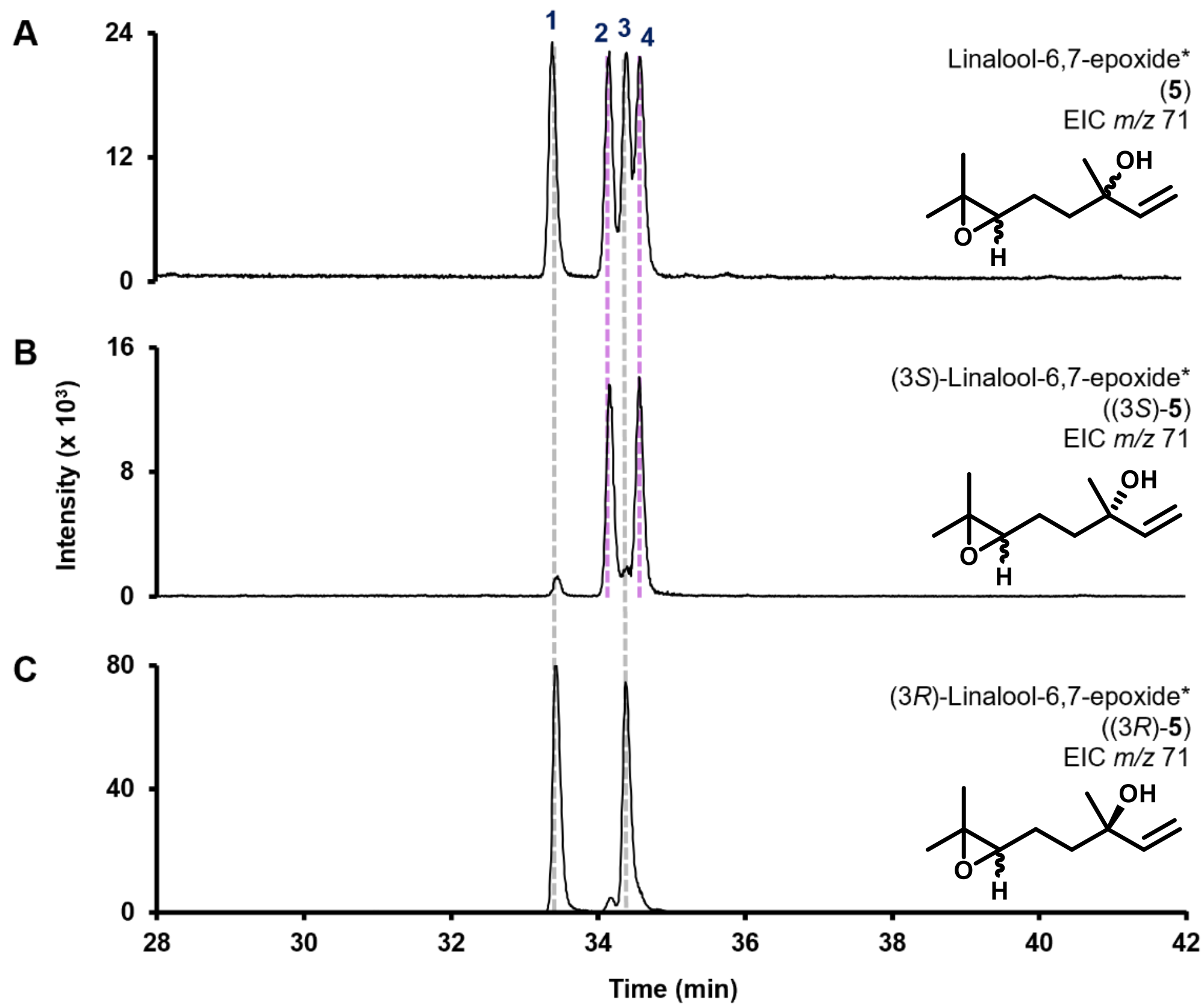

Linalool oxide (furanoid) ((5*S*)-**1** and (5*R*)-**1**) and linalool oxide (pyranoid) ((6*S*)-**4** and (6*R*)-**4**)

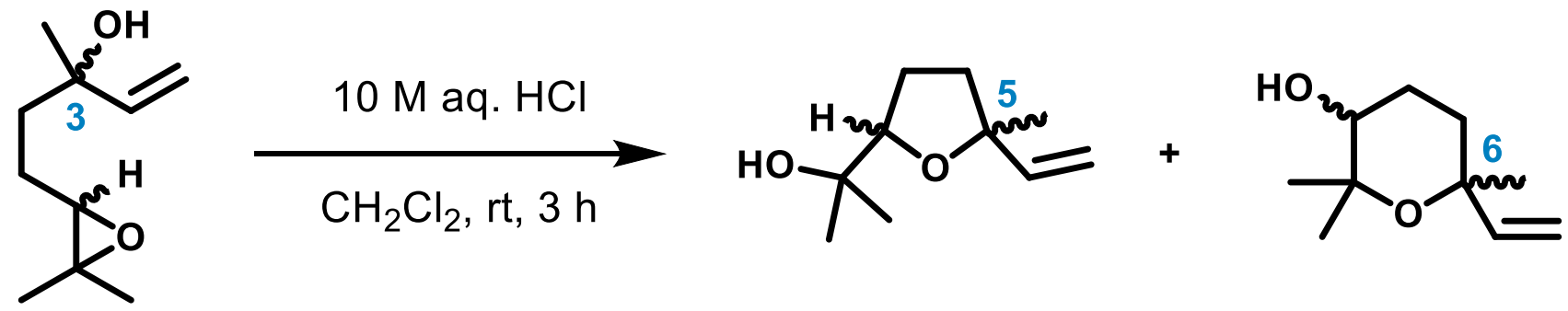

(3*S*)-**5**
(3*R*)-**5**

(5*S*)-**1**, 79%
(5*R*)-**1**, 75%

(6*S*)-**4**,10%
(6*R*)-**4**, 8%

To a 50 mL round bottom flask containing a solution of epoxide (3*S*)-**5** (1.7 g, 10 mmol) dissolved in CH_2Cl_2 (5 mL) was added 10 M aq. HCl (50 µL, 0.5 mmol). After stirring at room temperature for 3 h (complete consumption of starting material **5** was confirmed by TLC [Et_2O/pentane, 3:1]), the reaction mass was partitioned between CH_2Cl_2 (10 mL) and H_2O (20 mL). The resulting aqueous layer was repeatedly extracted with CH_2Cl_2 (10 mL x 3) and the combined organic phases were then dried over anhydrous Na_2SO_4 and carefully concentrated under reduced pressure to afford a colorless oil. Purification of the residue, using gradient flash column chromatography (0–20% Et_2O in pentane), afforded title compounds **1** and **4**.

Note: Following this procedure, (5*S*)-linalool oxide (furanoid) ((5*S*)-**1**) and (6*S*)-linalool oxide (pyranoid) ((6*S*)-**4**) were analogously prepared from (3*S*)-**5**. Similarly, (5*R*)-**1** and (6*R*)-**4** were synthesized from epoxide (3*R*)-**5**.

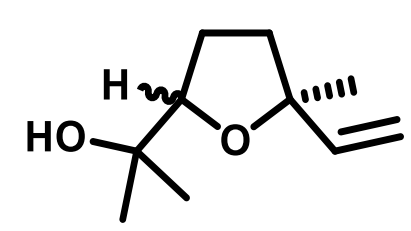

(5*S*)-**1**, 79%

(5*S*)-Linalool oxide (furanoid) ((5*S*)-**1**) was isolated as a colorless oil (1.342 g [79%] from 1.7 g [10 mmol] of (3*S*)-**5**): R_f 0.23 (Et_2O/pentane, 1:4); ^1H NMR ($CDCl_3$, 400 MHz) mixture of diastereoisomers* δ 5.97 (dd, J = 17.4, 10.8 Hz, 0.5H), 5.87 (dd, J = 17.3, 10.6 Hz, 0.5H), 5.21–5.16 (m, 1H), 5.02–4.98 (m, 1H), 3.86 (t, J = 6.9 Hz, 0.5H), 3.80 (t, J = 7.1 Hz, 0.5H), 2.00 (brs, 1H), 1.96–1.68 (m, 4H), 1.31 (s, 3H), 1.232 (s, 1.5H), 1.226 (s, 1.5H), 1.131 (s, 1.5H), 1.126 (s, 1.5H); GC-MS (EI) *m/z* (%) *2*) 59 (100), 94 (74), 111 (56), 93 (56), 68 (37), 55 (36), 67 (33), 81 (26), 83 (21), 79 (18), *4*) 59 (100), 94 (75), 93 (53), 111 (52), 68 (38), 55 (37), 67 (32), 81 (25), 83 (23), 79 (19). Physical and spectral data agreed with those reported previously [62]. *Both diastereoisomers of (5*S*)-**1** are described.

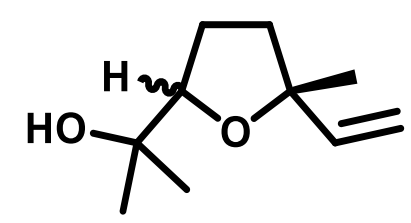

(5*R*)-**1**, 75%

(5*R*)-Linalool oxide (furanoid) ((5*R*)-**1**) was isolated as a colorless oil (1.273 mg [75%] from 1.697 g [9.97 mmol] of (3*R*)-**5**). Physical and spectral data agreed with those reported previously [62].

GC-MS EIC data for (**A**) linalool oxide (furanoid) **1**, (**B**) (5*S*)-**1**, and (**C**) (5*R*)-**1**. Elution order of **1** stereoisomers: *1*) (2*R*,5*R*)-**1**, *2*) (2*S*,5*S*)-**1**, *3*) (2*S*,5*R*)-**1**, and *4*) (2*R*,5*S*)-**1** [63]. Separation method: D. *Mixture of stereoisomers.

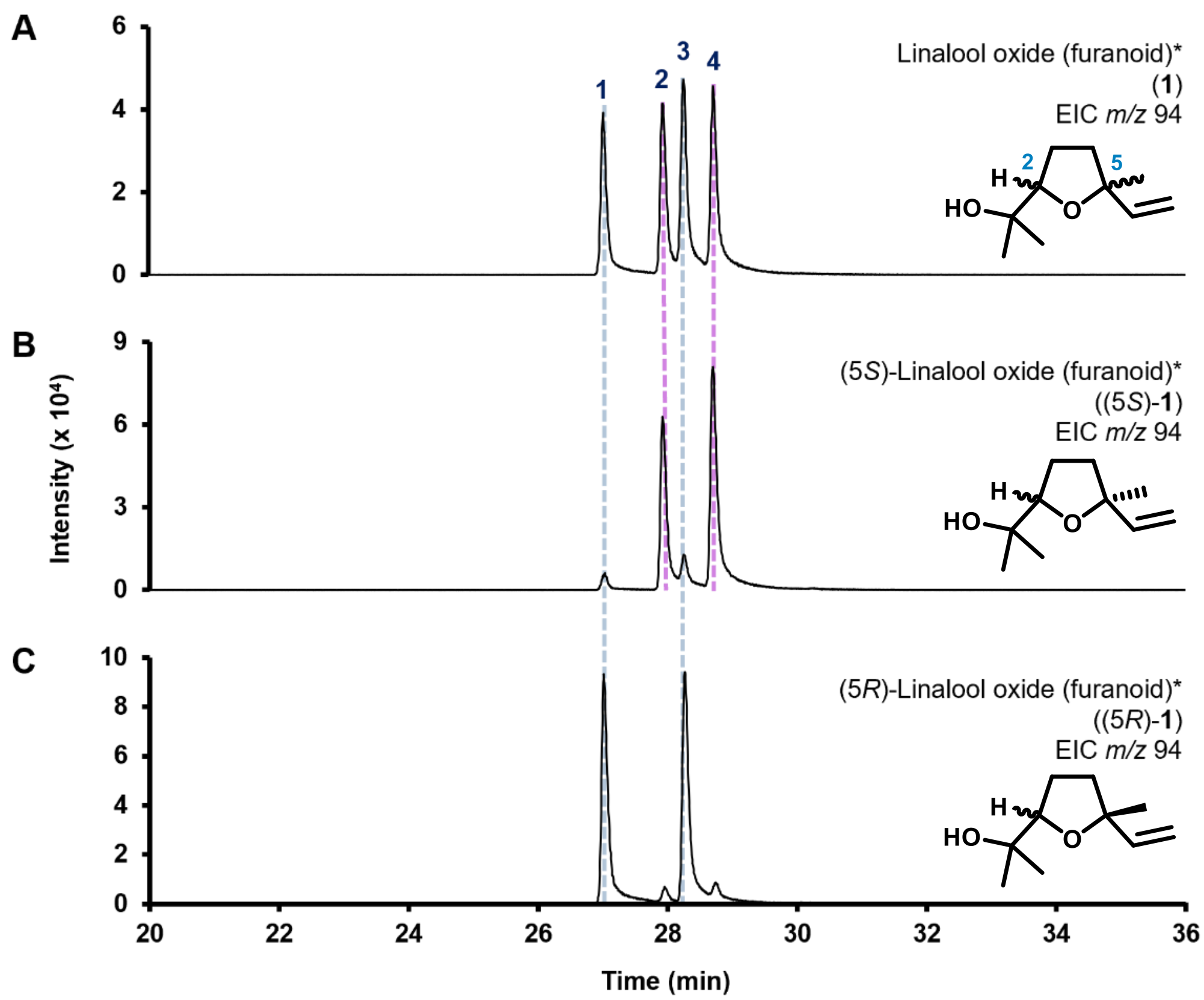

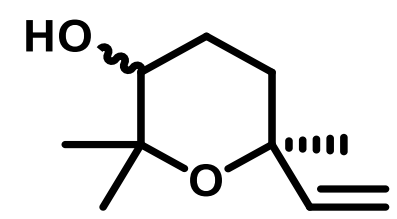

(6*S*)-**4**, 10%

(6*S*)-Linalool oxide (pyranoid) ((6*S*)-**4**) was isolated as a colorless wax (167 mg [10%] from 1.700 g [10 mmol] of (3*S*)-**5**): R_f 0.13 (Et_2O/pentane, 1:4); ^1H NMR ($CDCl_3$, 400 MHz) mixture of diastereoisomers* δ 6.00–5.90 (m, 1H), 5.04–4.95 (m, 2H), 3.45–3.39 (m, 1H), 2.12 (dt, *J* = 13.6, 3.8 Hz, 0.5H), 2.02–1.94 (m, 0.5H), 1.84–1.53 (m, 4H), 1.25 (overlapping s, 1.5H), 1.24 (overlapping s, 1.5H), 1.22 (s, 3H), 1.169 (overlapping s, 1.5H), 1.161 (overlapping s, 1.5H); GC-MS (EI) *m*/*z* (%) *1*) 68 (100), 94 (75), 59 (61), 67 (57), 79 (12), 53 (12), 83 (12), 69 (12), 55 (11), 79 (11), *3*) 68 (100), 94 (75), 59 (60), 67 (52), 79 (23), 69 (14), 84 (13), 53 (12), 81 (12), 55 (11). Physical and spectral data agreed with those reported previously [64]. *Both diastereoisomers of **4** are described.

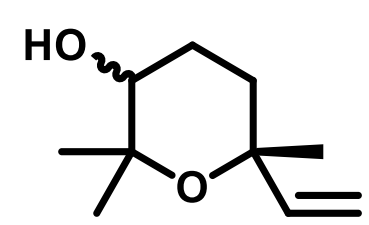

(6*R*)-**4**, 8%

(6*R*)-Linalool oxide (pyranoid) ((6*R*)-**4**) was isolated as a colorless wax (136 mg [8%] from 1.697 g [9.97 mmol] of (3*R*)-**5**). Physical and spectral data agreed with those reported previously [64].

GC-MS EIC data for (**A**) linalool oxide (pyranoid) **4**, (**B**) (6*S*)-**4**, and (**C**) (6*R*)-**4**. Elution order of **4** stereoisomers: *1*) (3*R*,6*S*)-**4**, *2*) (3*S*,6*R*)-**4**, *3*) (3*S*,6*S*)-**4**, and *4*) (3*R*,6*R*)-**4** [63]. Separation method: D. *Mixture of stereoisomers.

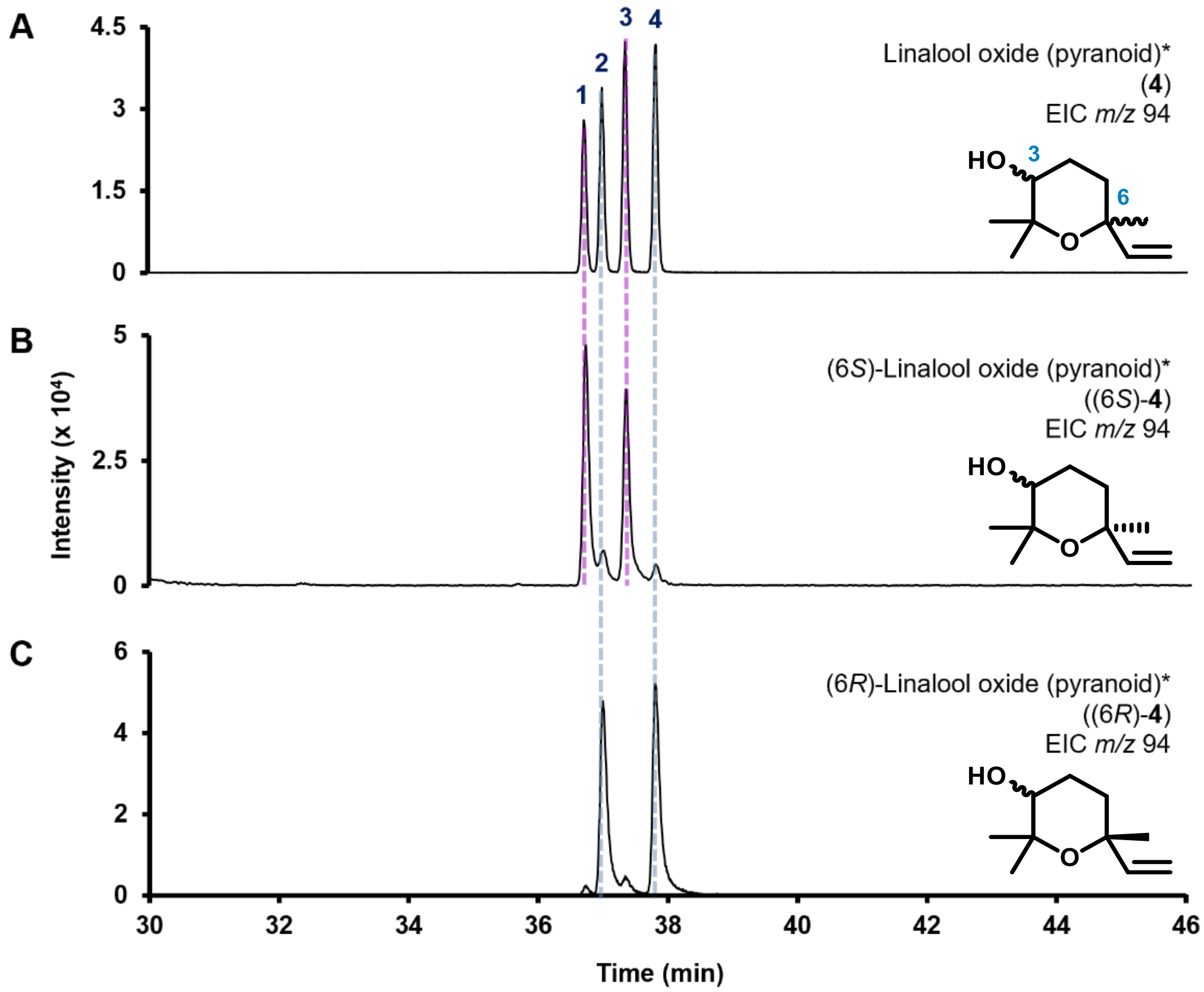

Synthesis of 8-Oxolinalool (7)

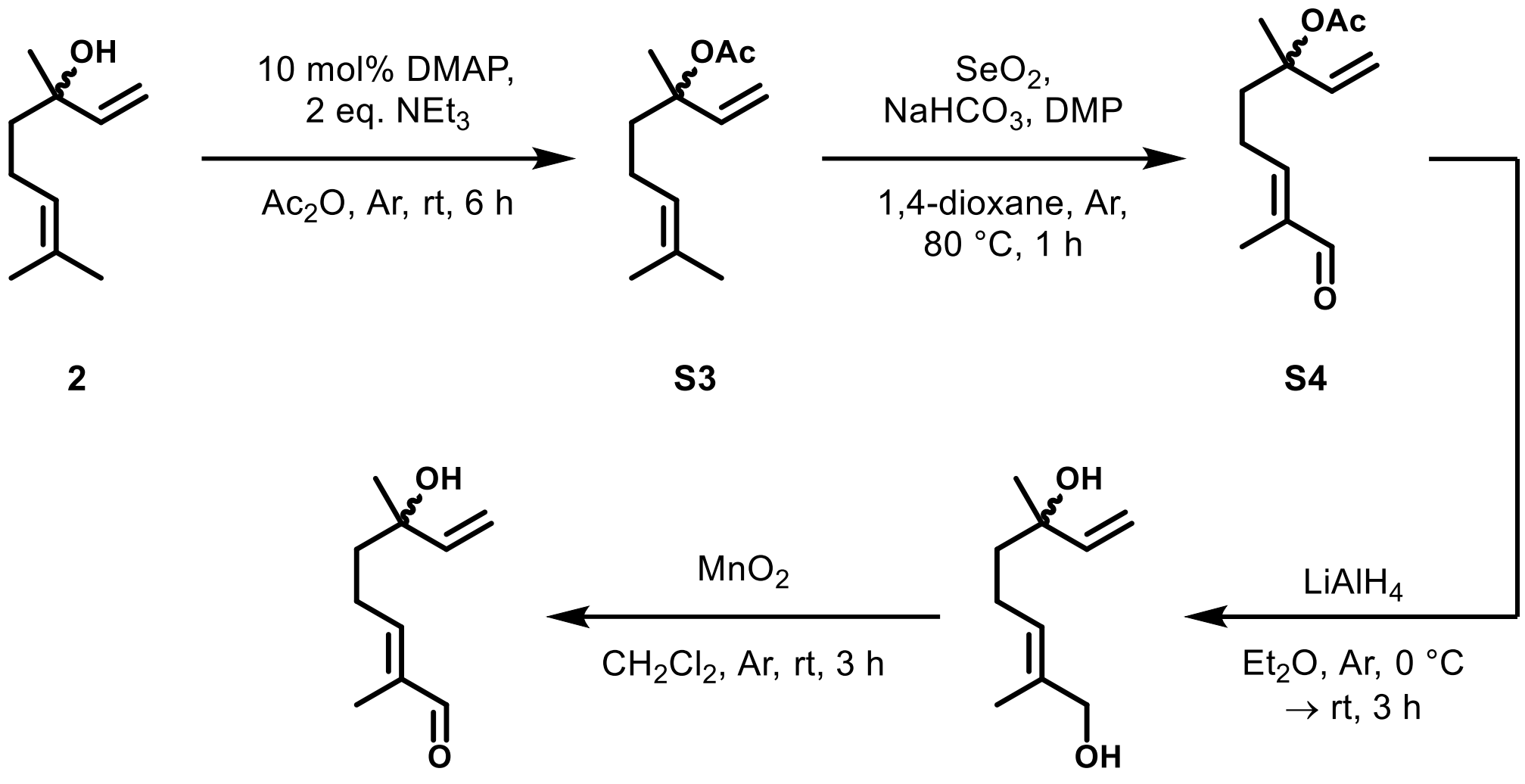

Linalyl acetate (**S3**)

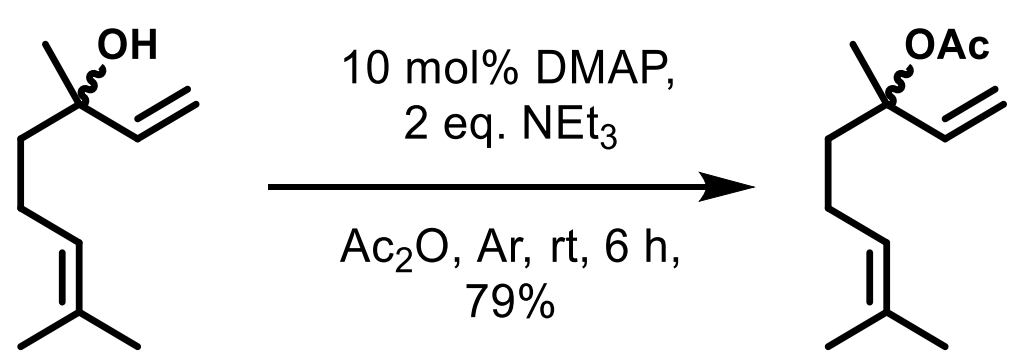

To a dry 100 mL round bottom flask under an Ar atmosphere containing *rac*-**2** (5 mL, 27.94 mmol) and 4-dimethylaminopyridine (341 mg, 2.79 mmol) was added acetic anhydride (5.28 mL, 55.88 mmol) and triethylamine (7.79 mL, 55.88 mmol). After stirring at room temperature for 6 h, the resulting deep amber solution was cooled to 0 °C, slowly charged with sat. aq. $NaHCO_3$ (100 mL), and allowed to stir for a further 30 min whilst warming to room temperature. The mixture was extracted with Et_2O (25 mL x 3) and the combined organic layers were sequentially washed with sat aq. $NaHCO_3$ (25 mL x 3), 0.1 M aq. HCl (25 mL x 2), H_2O (25 mL), and brine (25 mL). The organic phase was dried over anhydrous Na_2SO_4 and carefully concentrated under reduced pressure to afford a residue that was purified by gradient flash column chromatography (0–5% Et_2O in hexane) to furnish acetate *rac*-**S3** as a colorless oil (4.332 g, 79%): R_f 0.23 (Et_2O/hexane, 1:19); ^1H NMR ($CDCl_3$, 400 MHz) δ 5.97 (dd, J = 17.5, 11.0 Hz, 1H), 5.15 (dd, J = 17.4, 0.9 Hz, 1H), 5.12 (dd, J = 11.0, 0.9 Hz, 1H), 5.11–5.06 (overlapping m, 1H), 2.00 (s, 3H), 1.98–1.94 (m, 2H), 1.91–1.72 (m, 2H), 1.67 (appd, J = 0.8 Hz, 3H), 1.59 (s, 3H), 1.54 (s, 3H); GC-MS (EI) *m/z*

(%) 93 (100), 121 (36), 80 (35), 69 (21), 92 (17), 71 (14), 91 (14), 79 (13), 94 (13), 136 (12). Physical and spectral data agreed with those reported previously [65].

Note: Following this procedure, (*S*)-**S3** and (*R*)-**S3** were analogously prepared from (*S*)- and (*R*)-**2**, respectively.

GC-MS data for *rac*-**S3**, including (**A**) EIC and (**B**) EI-MS spectrum. Separation method: A

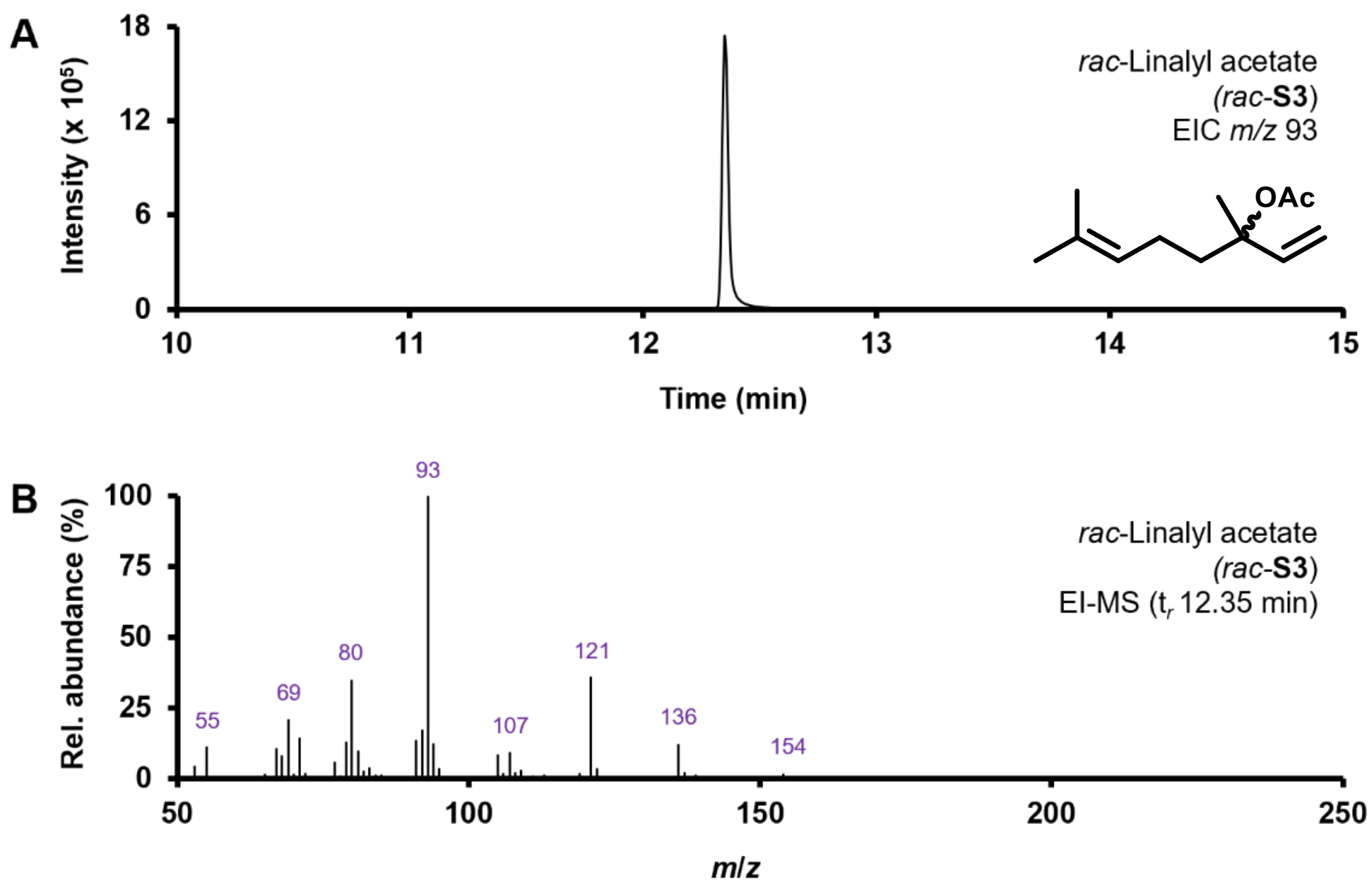

3,7-Dimethyl-8-oxoocta-1,6-dien-3-yl acetate (**S4**)

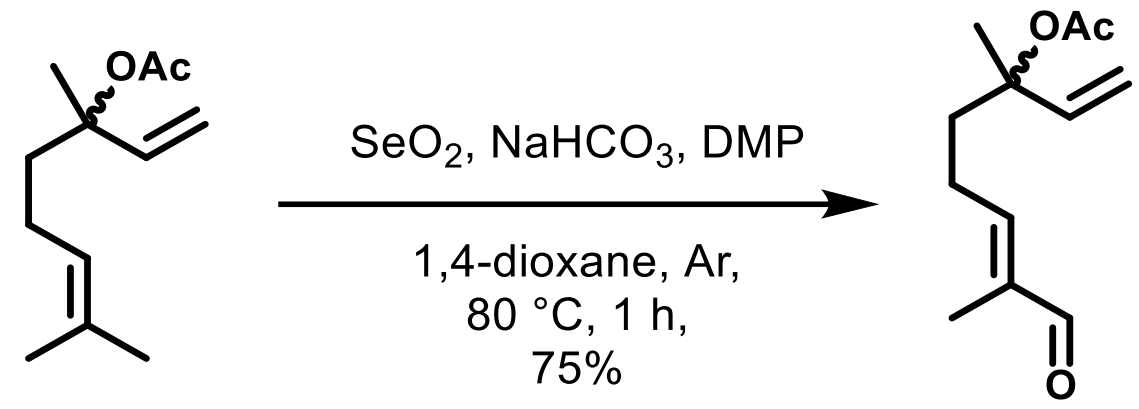

S3 **S4**

To a dry 250 mL round bottom flask under an Ar atmosphere, containing acetate **S3** (4.044 g, 20.60 mmol) and anhydrous 1,4-dioxane (41.2 mL), was sequentially added SeO_2 (4.572 g, 41.20 mmol), $NaHCO_3$ (3.461 g, 41.20 mmol), and Dess-Martin periodinane (17.475 g, 41.20 mmol). The beige suspension was heated to 80 °C (oil bath) for 1 h and then cooled to room temperature. The resulting burgundy suspension was passed through a short pad of Celite® and the wet cake was repeatedly washed with portions of Et_2O (20 mL x 5). Upon cooling to 0 °C, the combined filtrate was slowly poured onto a rapidly stirring ice-cold mixture of sat. aq. $NaHCO_3$ (50 mL) and sat. aq. $Na_2S_2O_3$ (50 mL). After warming to room temperature and stirring for 15 min, the mixture was extracted with Et_2O (50 mL x 3). The combined organic layers were washed with brine (20 mL) and dried over anhydrous Na_2SO_4. Careful concentration under reduced pressure afforded a residue that was distilled *in vacuo* (b.p. 147 °C/19 mbar) to afford α,β-unsaturated aldehyde *rac*-**S4** as a bright yellow oil (3.248 g, 75%): R_f 0.27 (Et_2O/pentane, 1:4); ^1H NMR ($CDCl_3$, 400 MHz) δ 9.38 (s, 1H), 6.48–6.44 (m, 1H), 5.95 (dd, *J* = 17.5, 11.0 Hz, 1H), 5.21–5.15 (m, 2H), 2.36 (q, *J* = 7.8 Hz, 2H), 2.12–2.04 (m, 1H), 2.01 (s, 3H), 1.94–1.86 (m, 1H), 1.73 (m, 3H), 1.58 (m, 3H); GC-MS (EI) *m*/*z* (%) 71 (100), 82 (98), 84 (67), 150 (66), 93 (63), 121 (63), 95 (60), 80 (52), 107 (49), 108 (46). Physical and spectral data agreed with those reported previously [66].

Note: Following this procedure, (3*S*)-**S4** and (3*R*)-**S4** were analogously prepared from (3*S*)- and (3*R*)-**S3**, respectively.

GC-MS data for *rac*-**S4**, including (**A**) EIC and (**B**) EI-MS spectrum. Separation method: A

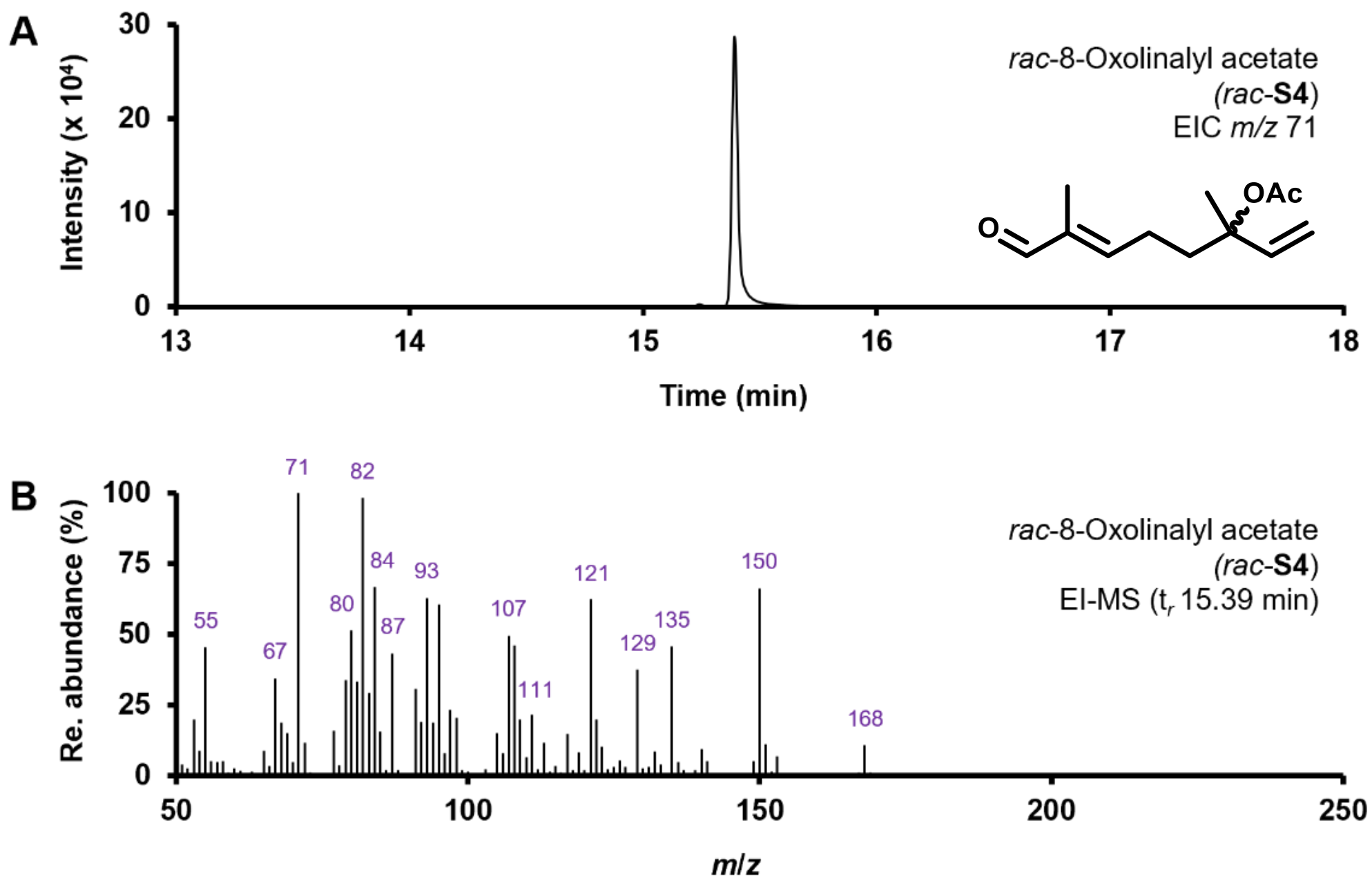

8-Hydroxylinalool (**8**)

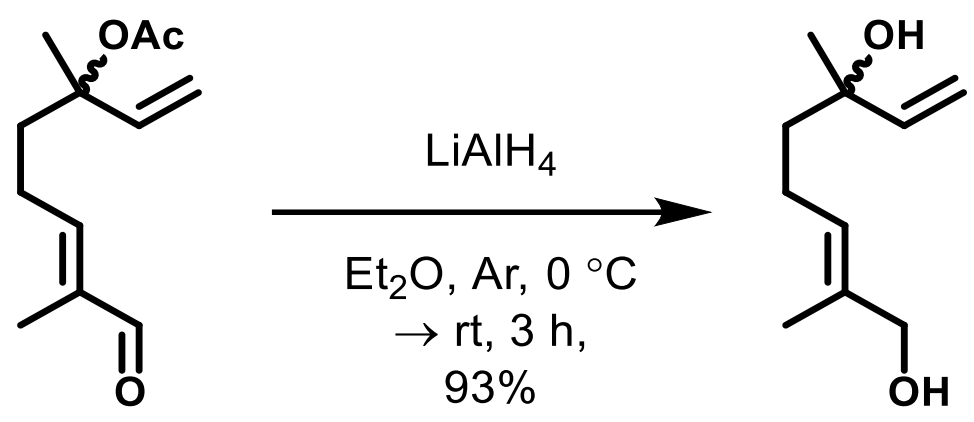

To a dry 25 mL round bottom flask under an Ar atmosphere, containing a suspension of $LiAlH_4$ (783 mg, 20.64 mmol) in anhydrous Et_2O (5 mL) cooled to 0 °C, was added dropwise a solution of aldehyde **S4** (868 mg, 4.13 mmol) in anhydrous Et_2O (5 mL). After warming to room temperature and stirring for 3 h (complete consumption of starting material **S4** was confirmed by TLC [Et_2O/pentane, 1:1]), the reaction mass was cooled to 0 °C and then cautiously treated dropwise with H_2O (0.78 mL), 15% (w/v) aq. NaOH (0.78 mL), and H_2O (2.34 mL). The resulting grey suspension was further diluted with Et_2O (10 mL) and stirred at room temperature for 15 min. Filtration of the colorless suspension through a short pad of Celite® gave a colorless filtrate that was dried over anhydrous $MgSO_4$ and carefully concentrated *in vacuo* to afford a light yellow residue which was then purified by gradient flash column chromatography (0–100% Et_2O in pentane) to yield diol **8** as a colorless oil (654 mg, 93%): R_f 0.34 (Et_2O); ^1H NMR ($CDCl_3$, 400 MHz) δ 5.91 (dd, J = 17.3, 10.7 Hz, 1H), 5.43–5.39 (m, 1H), 5.21 (dd, J = 17.4, 1.2 Hz, 1H), 5.07 (dd, J = 10.8, 1.2 Hz, 1H), 3.98 (s, 2H), 2.15–2.01 (m, 2H), 1.65 (s, 3H), 1.61–1.55 (m, 4H), 1.29 (s, 3H); GC-MS (EI) *m*/*z* (%) 71 (100), 67 (81), 55 (39), 68 (39), 82 (35), 93 (29), 79 (28), 81 (26), 84 (25), 110 (23). Physical and spectral data agreed with those reported previously [66].

Note: Following this procedure, (*S*)-**8** and (*R*)-**8** were analogously prepared from (*S*)- and (*R*)-**S4**, respectively.

GC-MS EIC data for (**A**) *rac*-8-hydroxylinalool (*rac*-**8**), (**B**) (*S*)-**8**, and (**C**) (*R*)-**8**. Separation method: E.

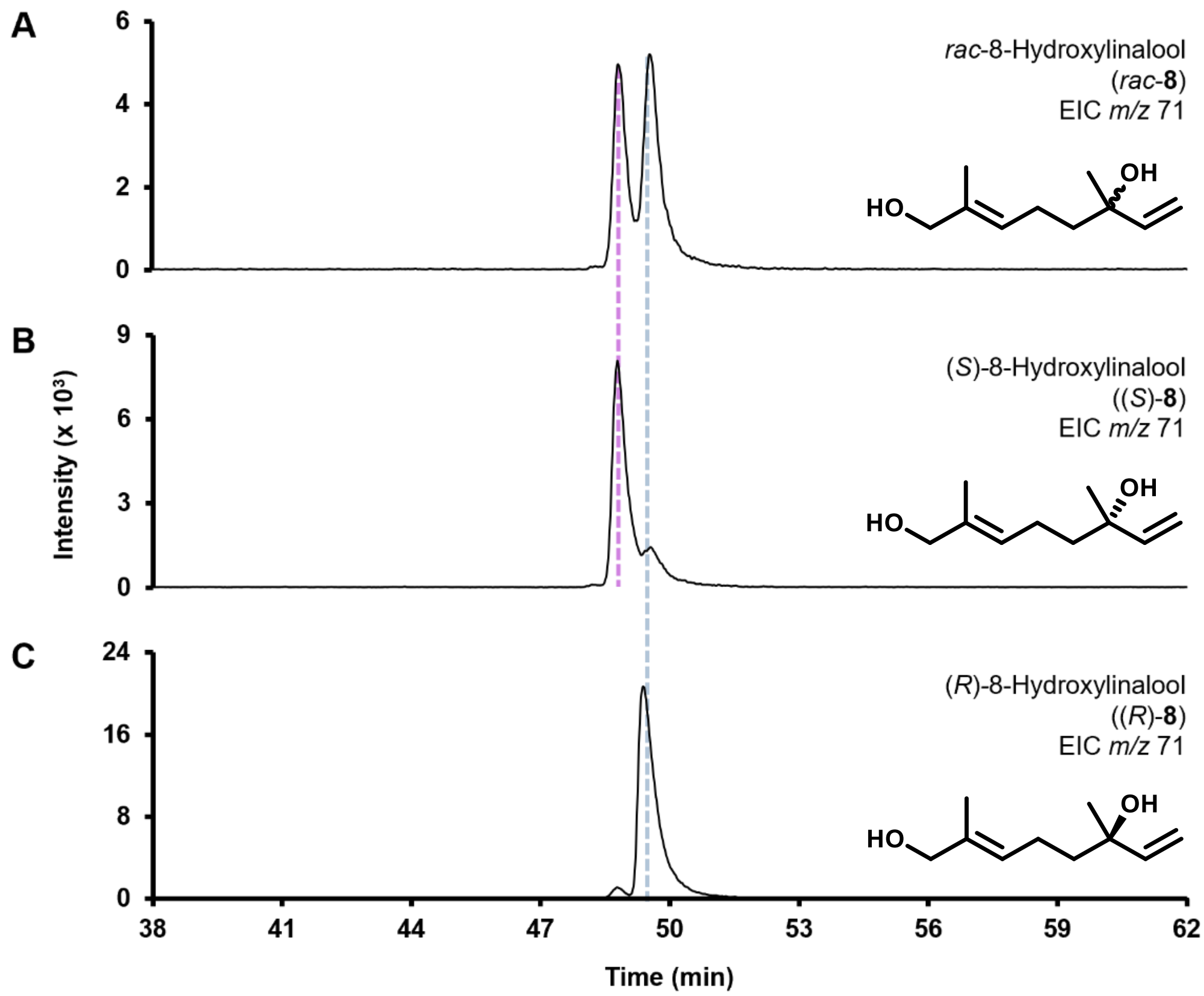

8-Oxolinalool (**7**)

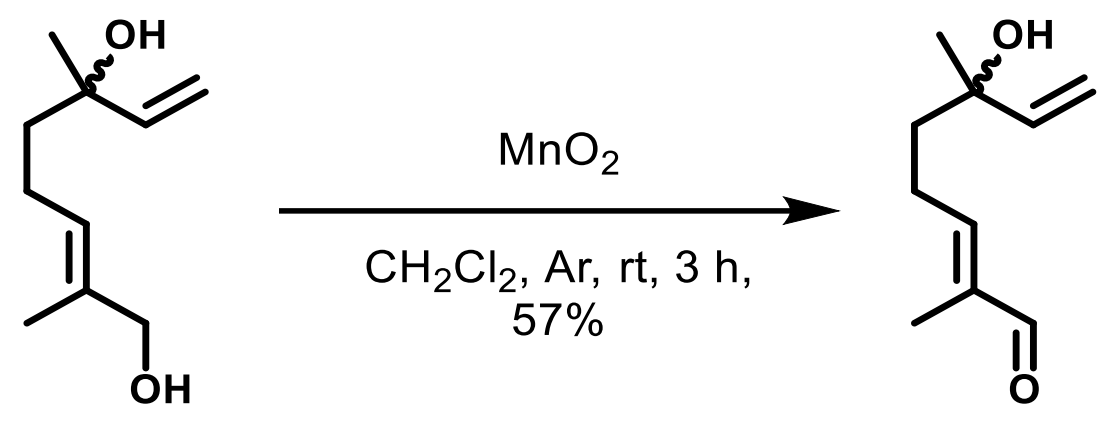

8 **7**

To a dry 25 mL round bottom flask under an Ar atmosphere, containing diol **8** (386 mg, 2.27 mmol) in anhydrous CH_2Cl_2 (11.5 mL), was added MnO_2 (2.960 g, 34.05 mmol). After stirring in darkness at room temperature for 3 h, the resulting black suspension was eluted through a short plug of silica with Et_2O (10 mL x 10). Careful concentration of the eluate under reduced pressure gave a colorless oil that was further purified by gradient flash column chromatography (0–50% Et_2O in hexane) to afford title compound **7** as a colorless oil (218 mg, 57%): R_f 0.28 (Et_2O/pentane, 1:1); ^{1}H NMR ($CDCl_3$, 400 MHz) *δ* 9.38 (d, *J* = 1.8 Hz, 1H), 6.51–6.47 (m, 1H), 5.92 (ddd, *J* = 17.3, 10.8, 1.2 Hz, 1H), 5.27–5.23 (m, 1H), 5.13–5.10 (m, 1H), 2.48–2.33 (m, 2H), 1.73 (s, 3H), 1.72–1.65 (m, 2H), 1.57 (brs, 1H), 1.33 (s, 3H); GC-MS (EI) *m*/*z* (%) 71 (100), 87 (34), 82 (30), 55 (28), 83 (26), 98 (20), 97 (15), 95 (14), 67 (14), 84 (13). Physical and spectral data agreed with those reported previously [67].

Note: Following this procedure, (*S*)-**7** and (*R*)-**7** were analogously prepared from (*S*)- and (*R*)-**8**, respectively.

GC-MS EIC chromatograms for (**A**) *rac*-8-oxolinalool (*rac*-**7**), (**B**) (*S*)-**7**, and (**C**) (*R*)-**7**. Separation method: F.

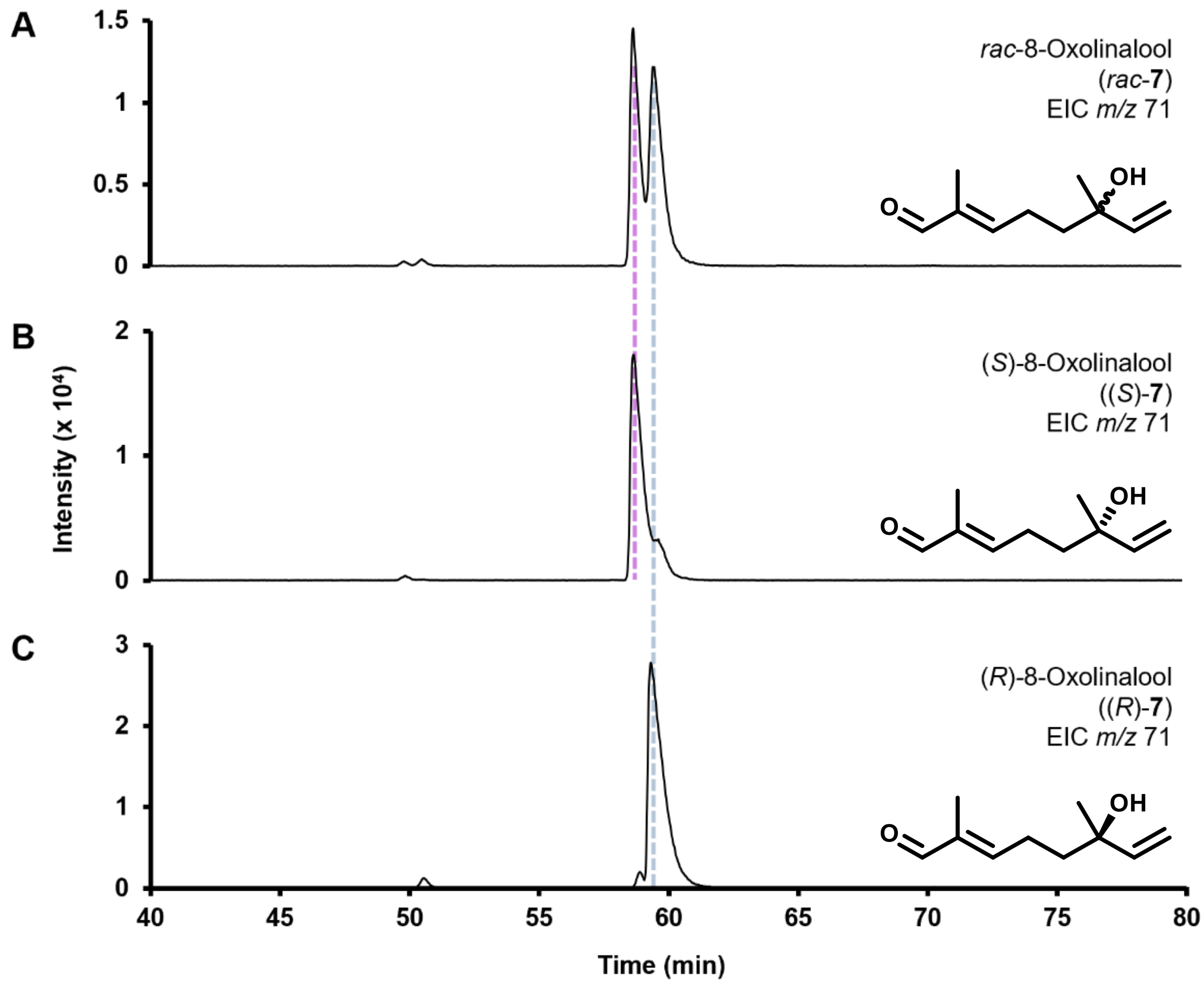

Synthesis of lilac aldehyde (3) and lilac alcohol (6)

Lilac alcohol **6** was prepared over two steps from **S4**, following Ohno's route [68].

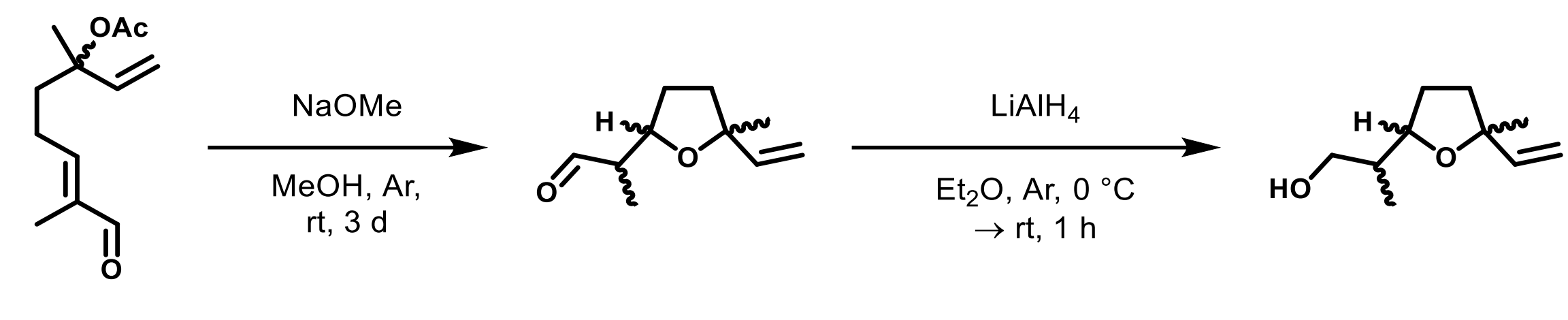

S4 **3** **6**

Lilac aldehyde (**3**)

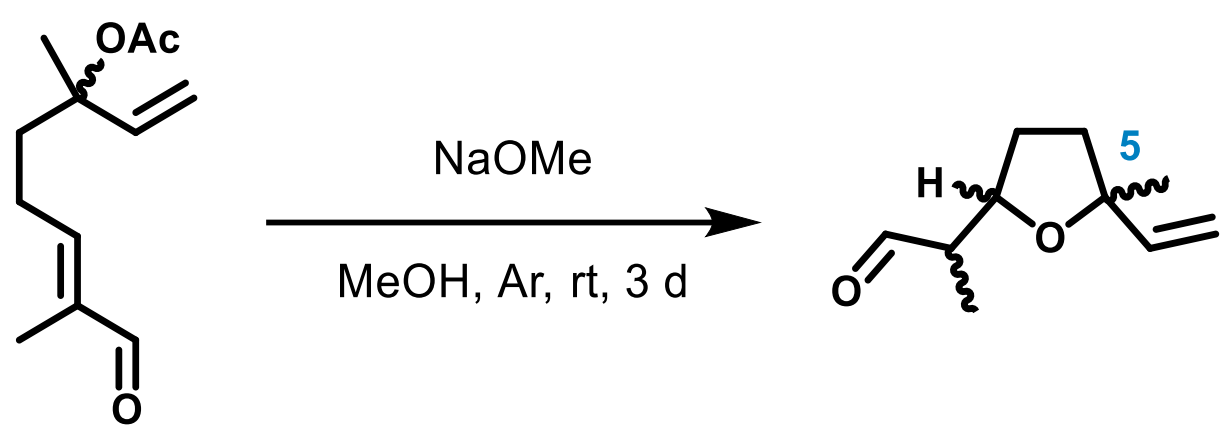

rac-**S4**	**3**, 15%
(3*S*)-**S4**	(5*S*)-**3**, 12%
(3*R*)-**S4**	(5*R*)-**3**, 18%

To a dry 250 mL round bottom flask under an Ar atmosphere, containing acetate *rac*-**S4** (3.233 g, 15.38 mmol) dissolved in anhydrous MeOH (154 mL), was added 5.4 M NaOMe in MeOH (1.14 mL, 6.15 mmol). After stirring in darkness at room temperature for 3 days, the resulting amber solution was adjusted to pH 7 using Dowex™ 50W X8 resin and filtered. The filtrate was dried over anhydrous Na_2SO_4 and carefully concentrated under reduced pressure to afford a residue that was then fractionally distilled *in vacuo* (b.p. 99–101 °C/31 mbar) to give a colorless oil that was further purified by gradient flash column chromatography (0–25% Et_2O in petroleum ether [b.p. 40–60 °C]) to yield title compound **3** as a colorless oil† (388 mg, 15%): R_f 0.23 and 0.20 (Et_2O/pentane, 1:9); ^1H NMR ($CDCl_3$, 400 MHz) mixture of stereoisomers* δ 9.81 (d, J = 2.5 Hz, 0.2H), 9.80 (d, J = 1.3 Hz, 0.2H), 9.79 (d, J = 2.4 Hz, 0.4H), 9.77 (d, J = 1.6 Hz, 0.2H), 5.94–5.81 (m, 1H), 5.22–5.15 (m, 1H), 5.03–4.97 (m, 1H), 4.29–4.11 (m, 1H), 2.64–2.46 (m, 1H), 2.11–2.00 (m, 1H), 1.96–1.86 (m, 1H), 1.82–1.66 (m, 2H), 1.31 (d, J = 3.0 Hz, 3H), 1.16 (d, J = 7.0 Hz, 0.6H), 1.12 (d, J = 7.0 Hz, 0.7H), 1.07 (d, J = 7.0 Hz, 1H), 1.04 (d, J = 7.0 Hz, 0.8H). Physical and spectral data agreed with those reported previously [68]. *All four diastereoisomers of **3** are described; †Lilac aldehyde **3** is highly volatile and also degrades upon standing at room temperature. Following isolation, **3** should be immediately stored at −20 °C under Ar.

Note: Following this procedure, (5*R*)-**3** and (5*S*)-**3** were analogously prepared from (*R*)- and (*S*)-**S4**, respectively.

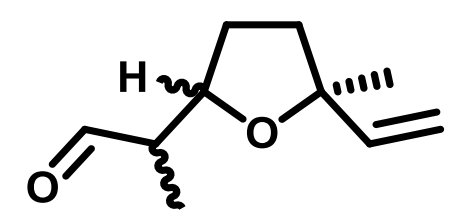

(5*S*)-**3**, 12%

(5*S*)-lilac aldehyde ((5*S*)-**3**) was isolated as a colorless oil (249 mg [12%] from 2.595 g [12.34 mmol] of (*S*)-**3**): GC-MS (EI) *m*/*z* (%) *1*) 55 (100), 93 (68), 111 (65), 67 (52), 71 (43), 69 (36), 110 (34), 81 (32), 68 (30), 153 (27), *2*) 55 (100), 93 (64), 71 (55), 111 (53), 67 (48), 69 (36), 81 (31), 68 (31), 153 (28), 110 (27), *7*) 55 (100), 111 (86), 93 (70), 67 (42), 69 (38), 71 (29), 81 (28), 110 (27), 153 (26), 68 (24), *8*) 55 (100), 93 (60), 111 (58), 71 (43), 67 (38), 69 (35), 153 (30), 81 (26), 68 (24), 110 (20). Physical and spectral data agreed with those reported previously [68].

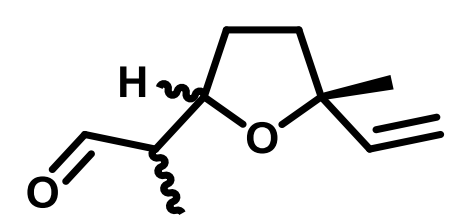

(5*R*)-**3**, 18%

(5*R*)-lilac aldehyde ((5*R*)-**3**) was isolated as a colorless oil (454 mg [18%] from 3.154 g [15 mmol] of (*R*)-**3**). Physical and spectral data agreed with those reported previously [68].

GC-MS EIC data for (**A**) lilac aldehyde **3**, (**B**) (5*S*)-**3**, and (**C**) (5*R*)-**3**. Elution order of **3** stereoisomers: *1*) (2´*S*,2*S*,5*S*)-**3**, *2*) (2´*R*,2*S*,5*S*)-**3**, *3*) (2´*R*,2*R*,5*R*)-**3**, *4*) (2´*S*,2*R*,5*R*)-**3**, *5*) (2´*S*,2*S*,5*R*)-**3**, *6*) (2´*R*,2*S*,5*R*)-**3**, *7*) (2´*S*,2*R*,5*S*)-**3**, and *8*) (2´*R*,2*R*,5*S*)-**3** [69]. Separation method: C. *Mixture of stereoisomers.

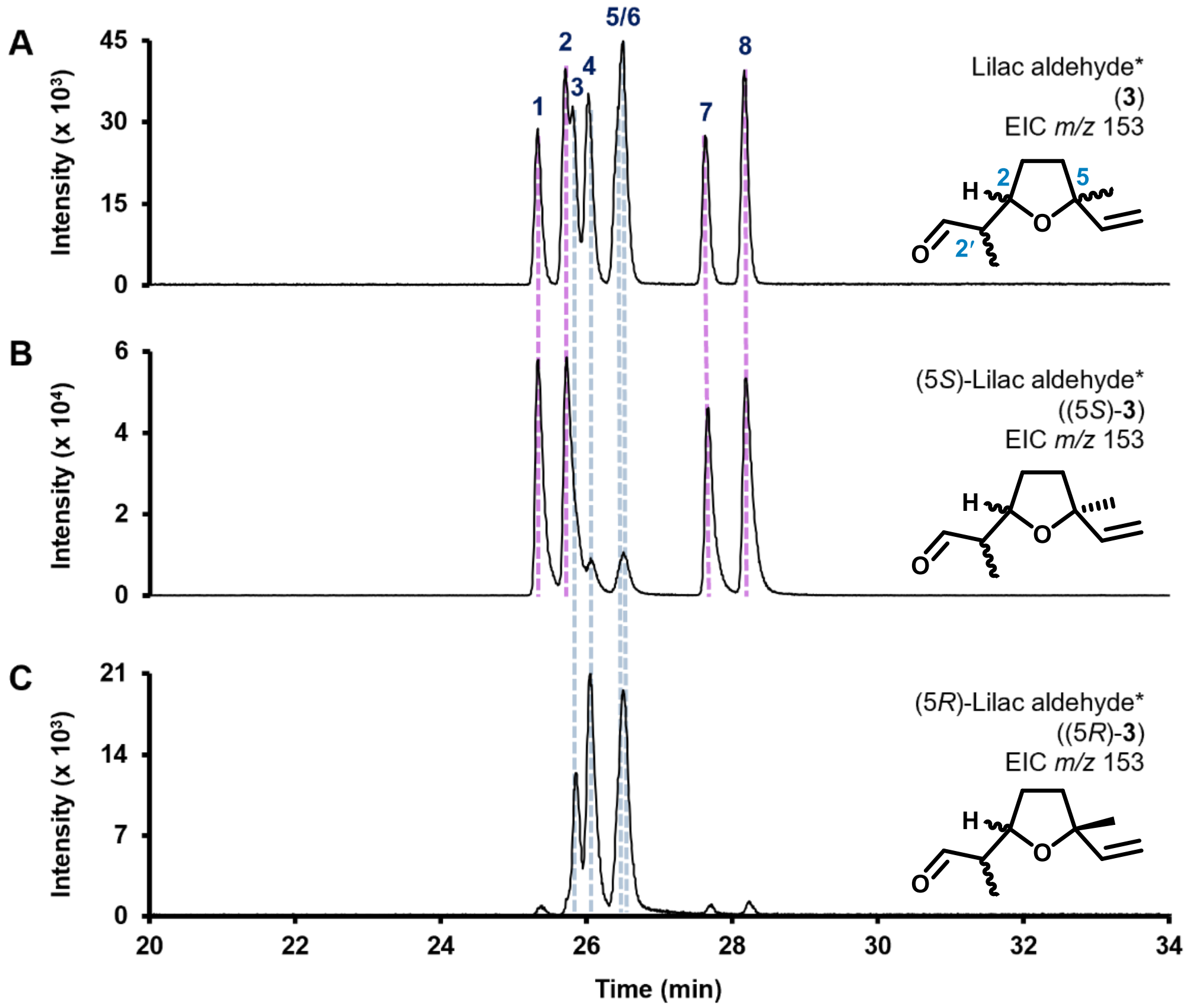

Lilac alcohol (**6**)

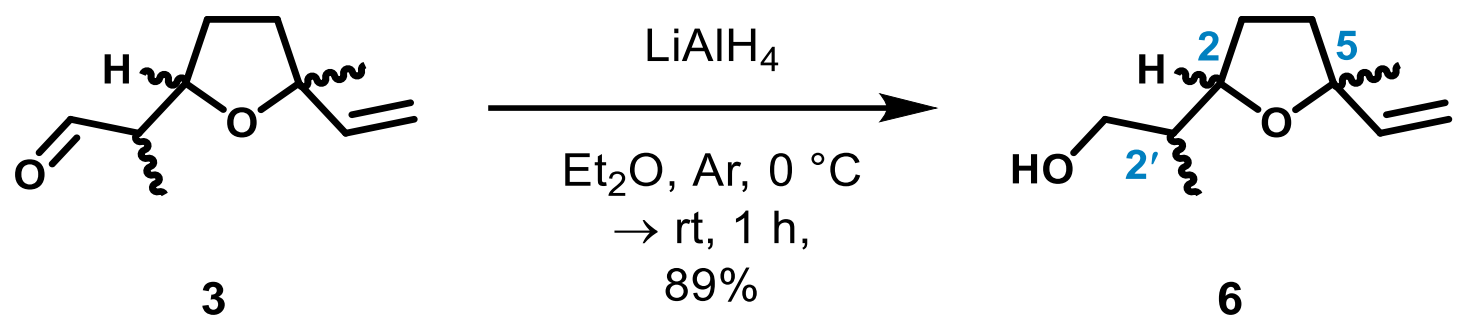

To a dry 8 mL vial under an Ar atmosphere, containing $LiAlH_4$ (54 mg, 1.42 mmol) suspended in anhydrous Et_2O (0.5 mL) at 0 °C, was added dropwise a solution of aldehyde **3** (100 mg, 0.59 mmol) in anhydrous Et_2O (0.6 mL). After warming to room temperature and stirring for 1 h the resulting suspension was cooled to 0 °C and cautiously treated stepwise with H_2O (0.05 mL), 15% (w/v) aq. NaOH (0.05 mL), H_2O (1.5 mL), and Et_2O (2 mL). Upon stirring at room temperature for 30 min, the grey mixture was filtered through a short pad of neutral Al_2O_3 and the resulting filtrate carefully concentrated under reduced pressure to afford title compound **6** as a colorless oil (89 mg, 89%): R_f 0.63 (Et_2O); ^{1}H NMR ($CDCl_3$, 400 MHz) mixture of stereoisomers* δ 5.97–5.81 (m, 1H), 5.23–5.14 (m, 1H), 5.01–4.97 (m, 1H), 4.16–4.10 (m, 0.4H), 3.86–3.77 (m, 0.6H), 3.72–3.55 (m, 2H), 2.07–1.94 (m, 1H), 1.93–1.55 (m, 5H), 1.31 (s, 3H), 0.94 (dd, J = 7.0 Hz, 0.3H), 0.90 (dd, J = 7.0 Hz, 0.9H), 0.80 (dd, J = 6.8 Hz, 0.8H), 0.78 (dd, J = 6.5 Hz, 1H). Physical and spectral data agreed with those reported previously.[68] *All four diastereoisomers of **6** are described.

Note: Following this procedure, (5*R*)-**6** and (5*S*)-**6** were analogously prepared from (5*R*)- and (5*S*)-**3**, respectively.

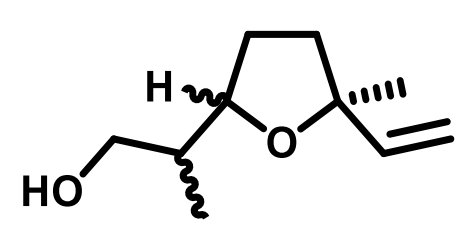

(5*S*)-**6**, 87%

(5*S*)-lilac alcohol ((5*S*)-**6**) was isolated as a colorless oil (132 mg [87%] from 150 mg [0.89 mmol] of (5*S*)-**3**): GC-MS (EI) *m*/*z* (%) *1*) 111 (100), 93 (77), 55 (60), 67 (33), 71 (25), 81 (21), 69 (19), 155 (18), 83 (12), 91 (11), *4*) 111 (100), 93 (78), 55 (63), 67 (30), 71 (24), 81 (20), 155 (19), 69 (19), 83 (12), 91 (11), *5*) 111 (100), 93 (79), 55 (62), 67 (37), 71 (22), 81 (22), 69 (19), 155 (16), 83 (13), 91 (11), *8*) 111 (100), 93 (82), 55 (70), 67 (35), 71 (27), 81 (24), 155 (22), 69 (19), 59 (18), 83 (13). Physical and spectral data agreed with those reported previously [68].

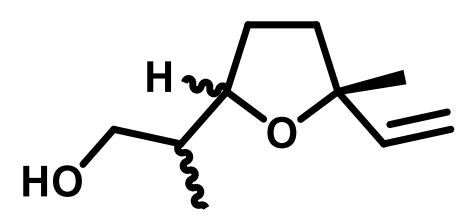

(5*R*)-**6**, 79%

(5*R*)-lilac alcohol ((5*R*)-**6**) was isolated as a colorless oil (120 mg [79%] from 150 mg [0.89 mmol] of (5*R*)-**3**). Physical and spectral data agreed with those reported previously [68].

GC-MS EIC data for (**A**) lilac alcohol **6**, (**B**) (5*S*)-**6**, and (**C**) (5*R*)-**6**. Elution order of **6** stereoisomers: *1*) (2´*S*,2*S*,5*S*)-**6**, *2*) (2´*R*,2*R*,5*R*)-**6**, *3*) (2´*R*,2*S*,5*R*)-**6**, *4*) (2´*S*,2*R*,5*S*)-**6**, *5*) (2´*R*,2*S*,5*S*)-**6**, *6*) (2´*S*,2*R*,5*R*)-**6**, *7*) (2´*S*,2*S*,5*R*)-**6**, and *8*) (2´*R*,2*R*,5*S*)-**6** [69]. Separation method: C. *Mixture of stereoisomers.

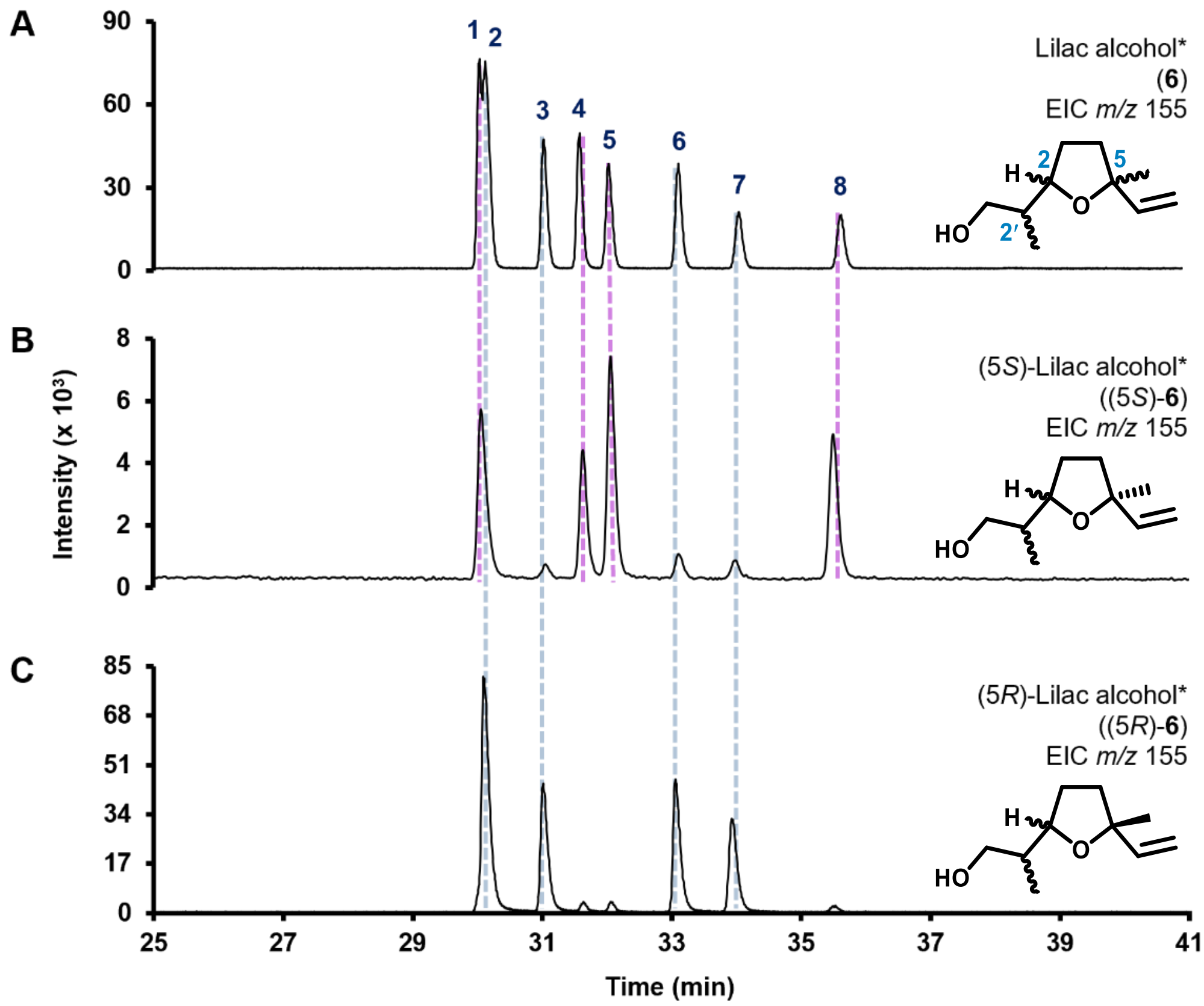

NMR Data

^{1}H NMR ($CDCl_3$, 400 MHz) **5**

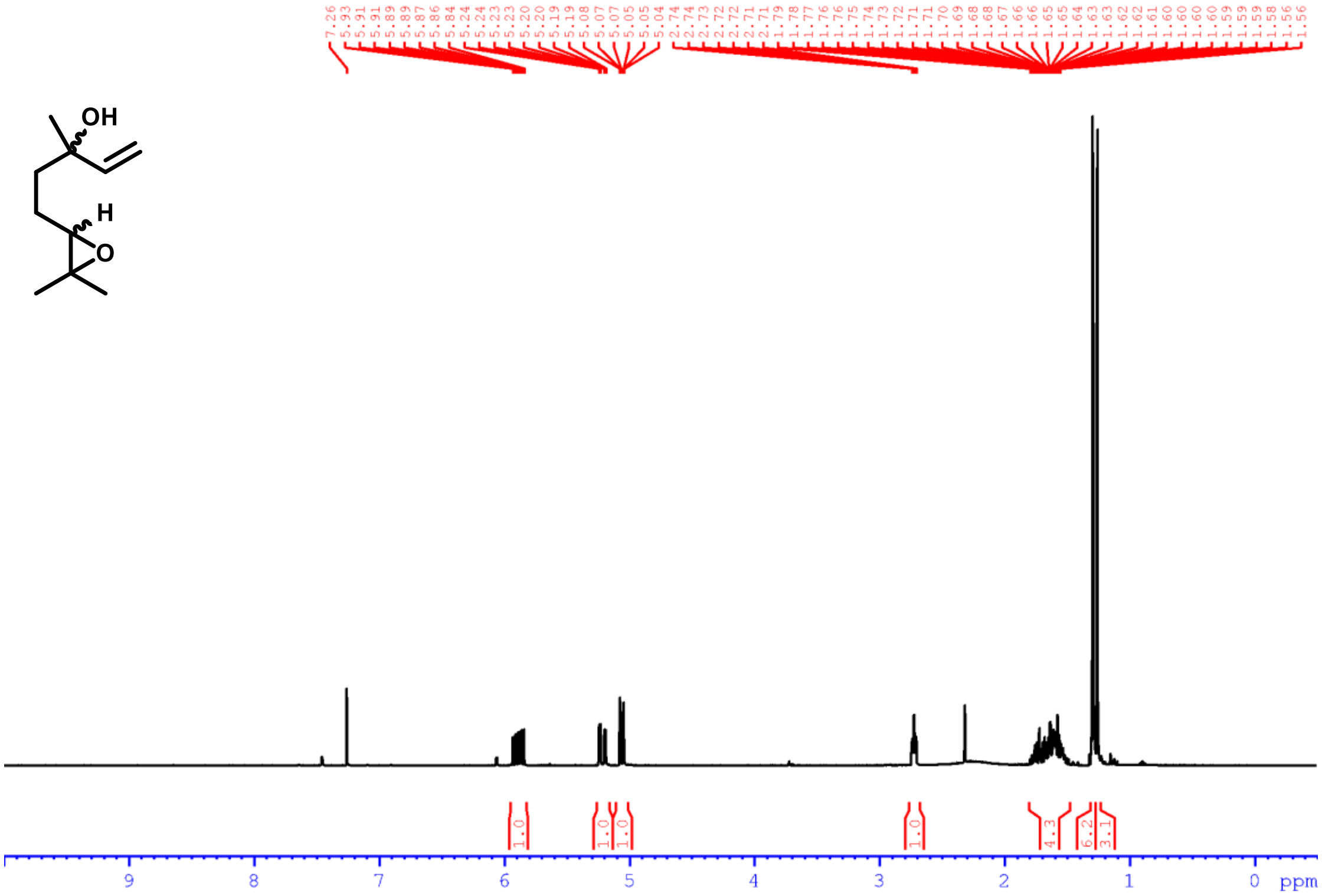

^{1}H NMR ($CDCl_3$, 400 MHz) (5*S*)-**1**

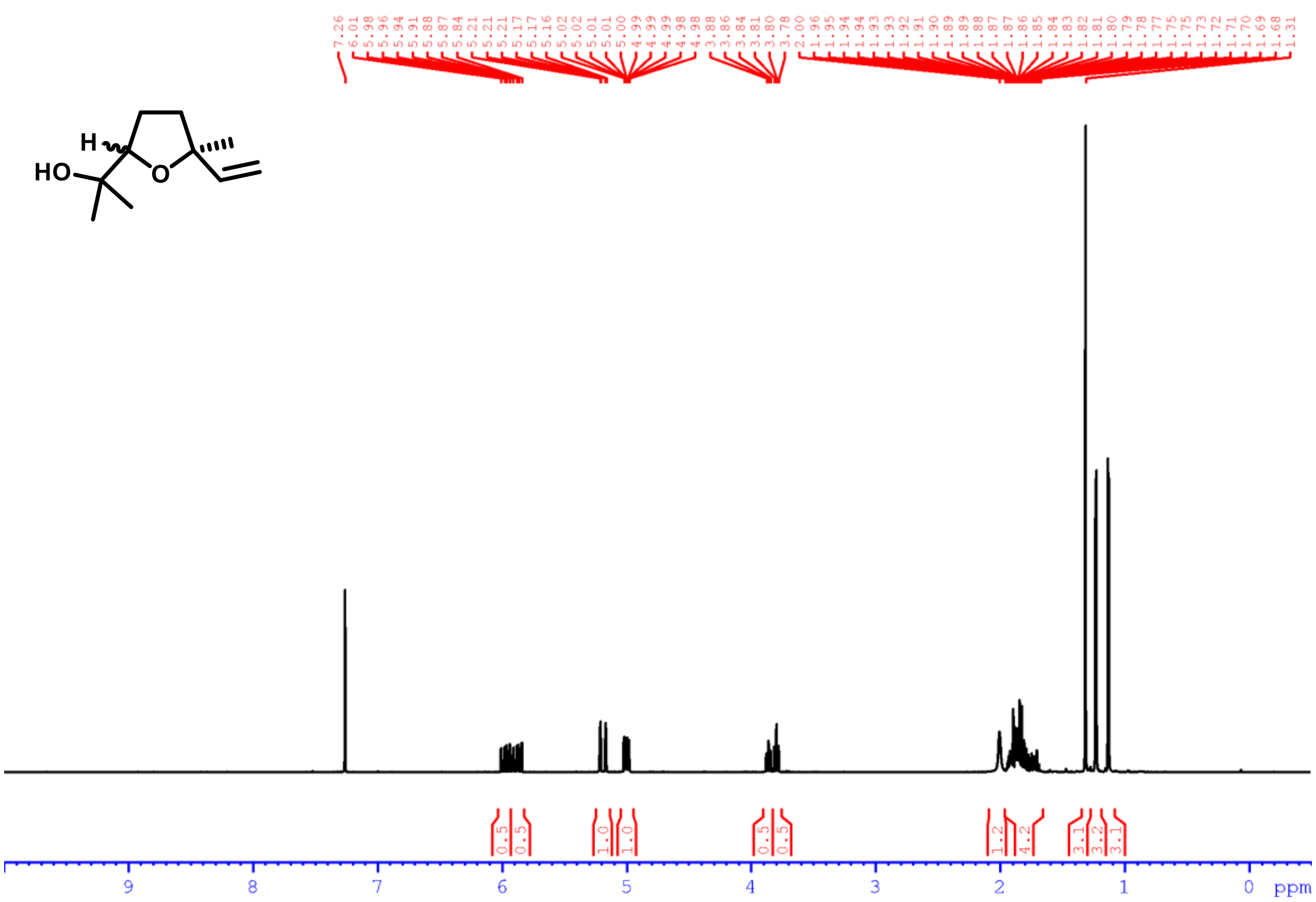

^{1}H NMR ($CDCl_3$, 400 MHz) (6*S*)-**4**

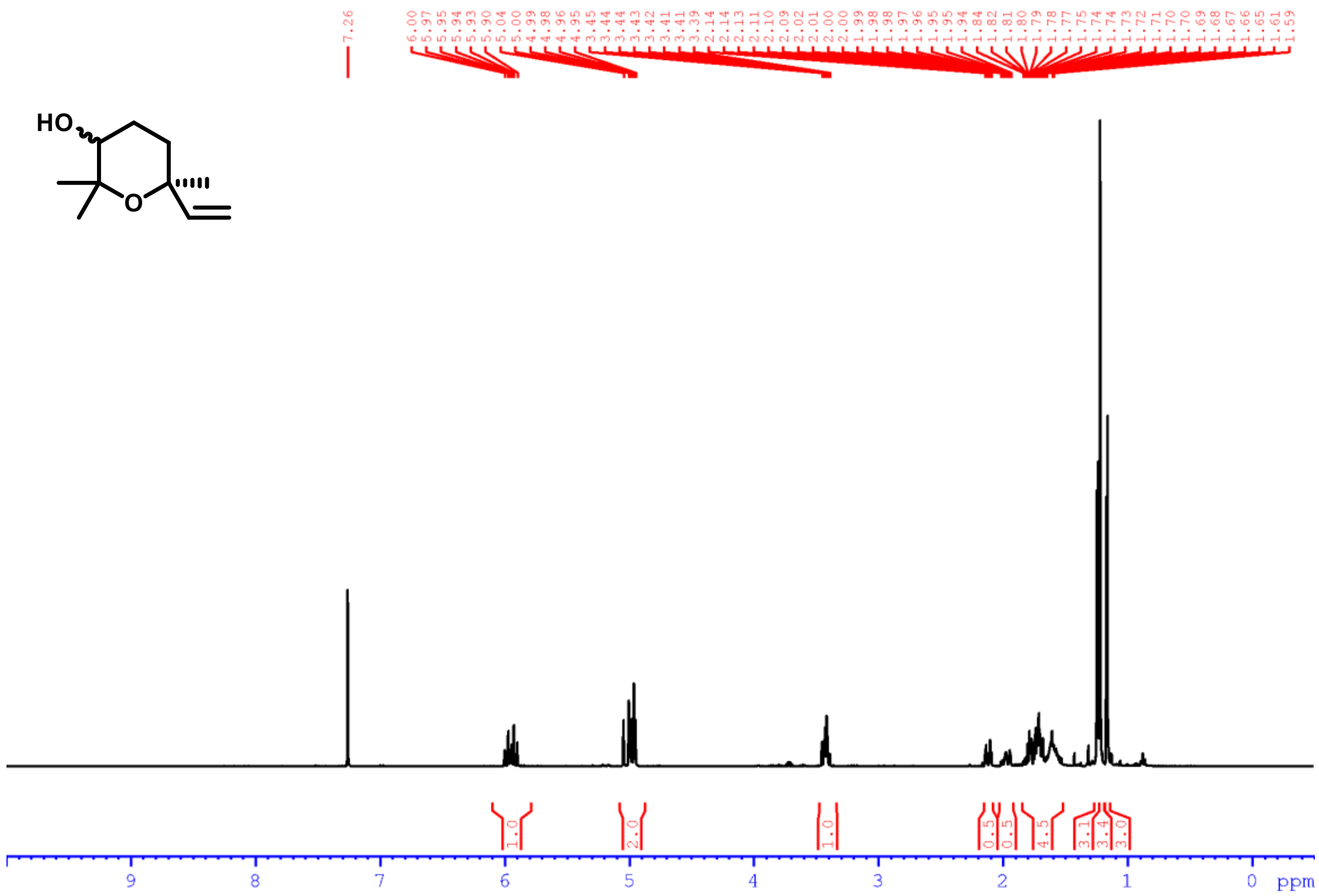

^{1}H NMR ($CDCl_3$, 400 MHz) *rac*-**S3**

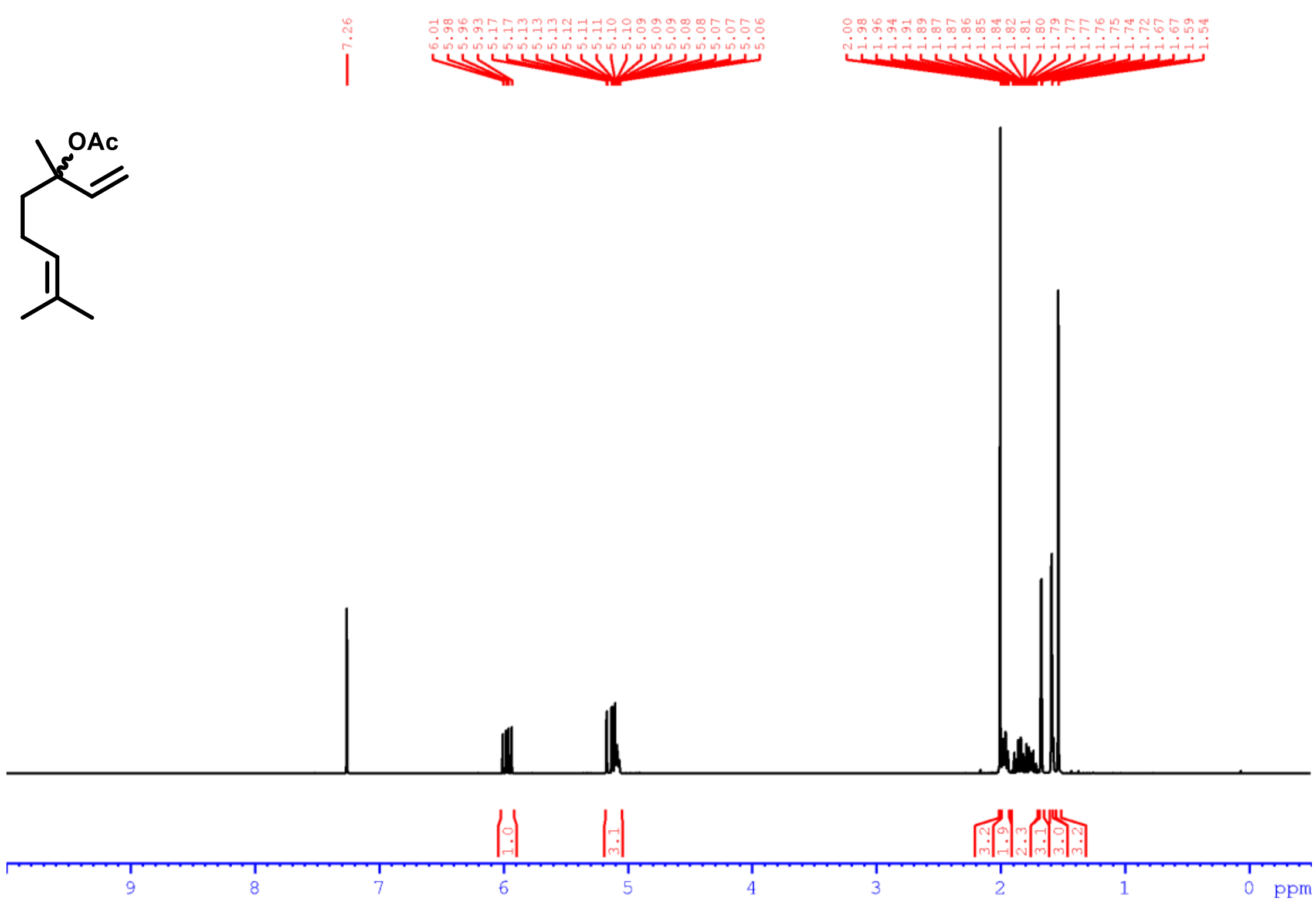

^{1}H NMR ($CDCl_3$, 400 MHz) *rac*-**S4**

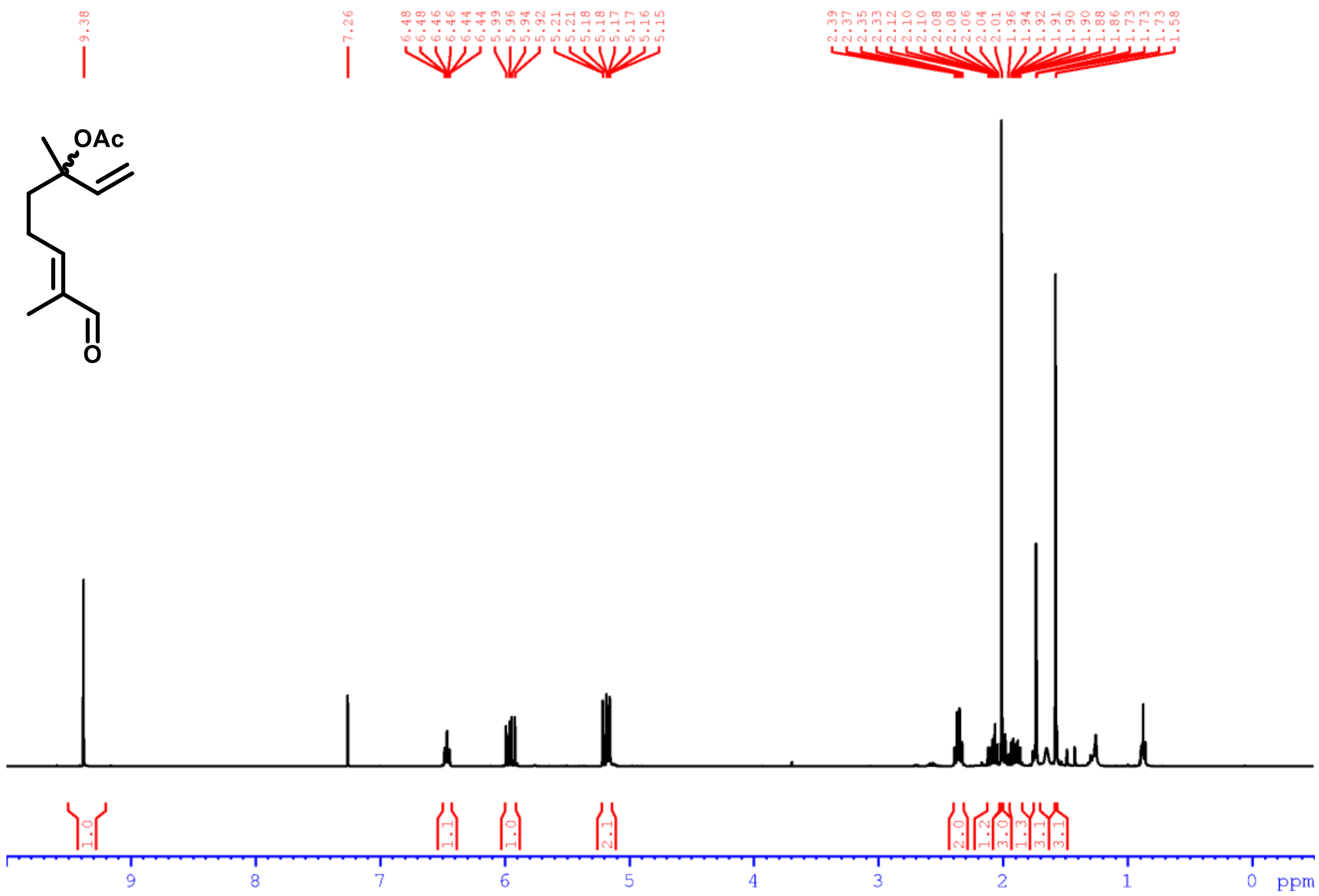

^{1}H NMR ($CDCl_3$, 400 MHz) *rac*-**8**

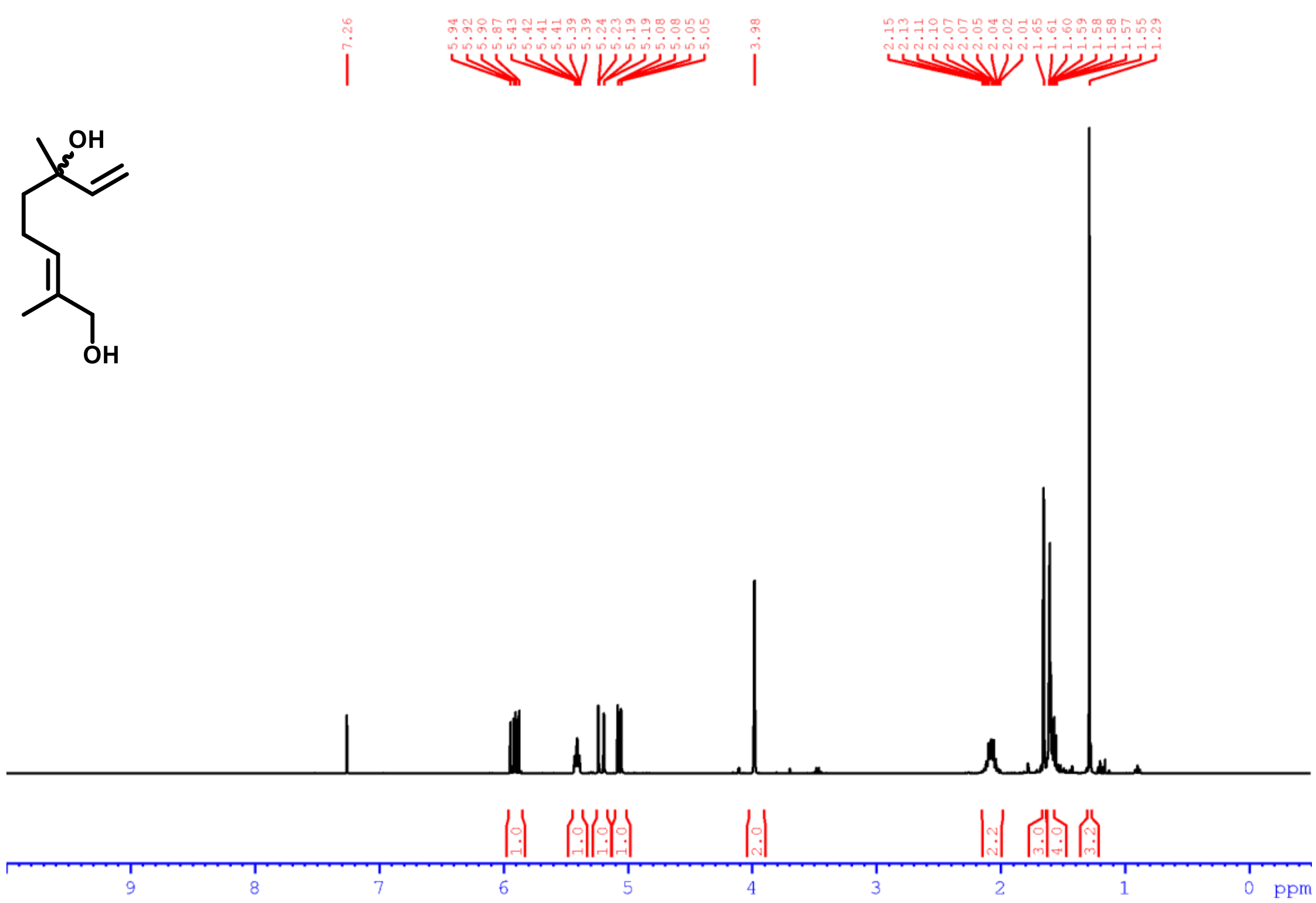

^1H NMR ($CDCl_3$, 400 MHz) *rac*-**7**

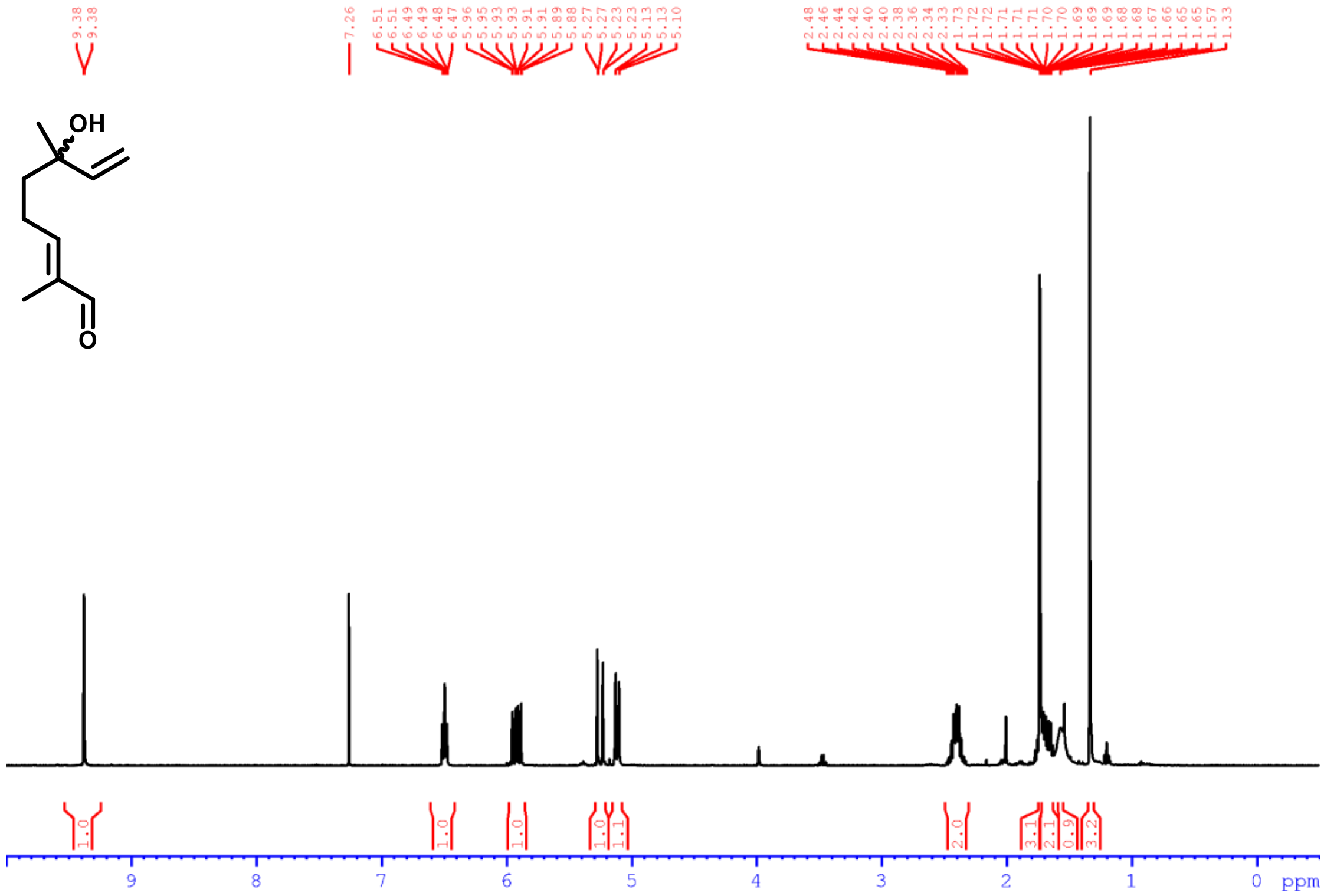

^1H NMR ($CDCl_3$, 400 MHz) **3**

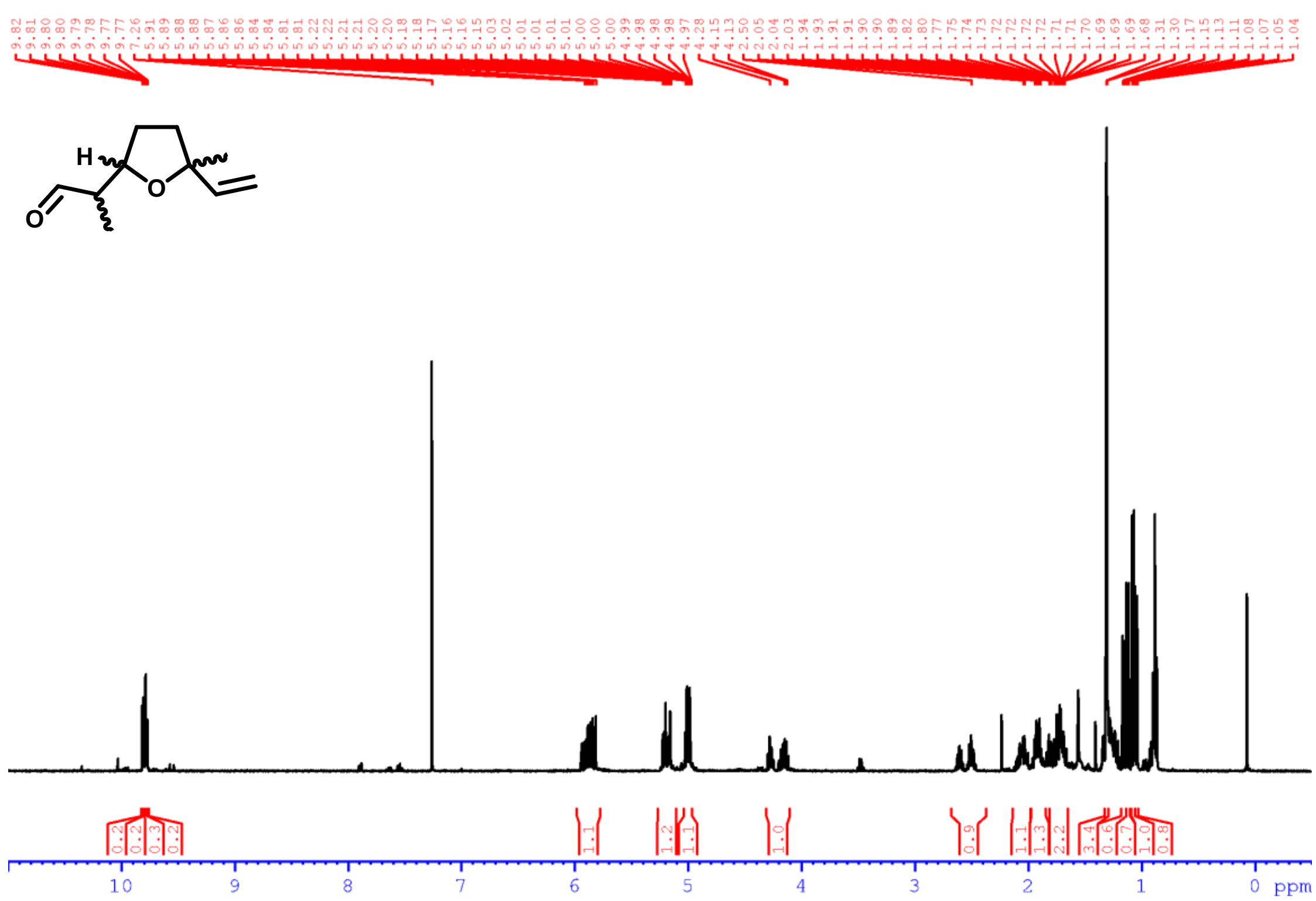

^{1}H NMR ($CDCl_3$, 400 MHz) **6**

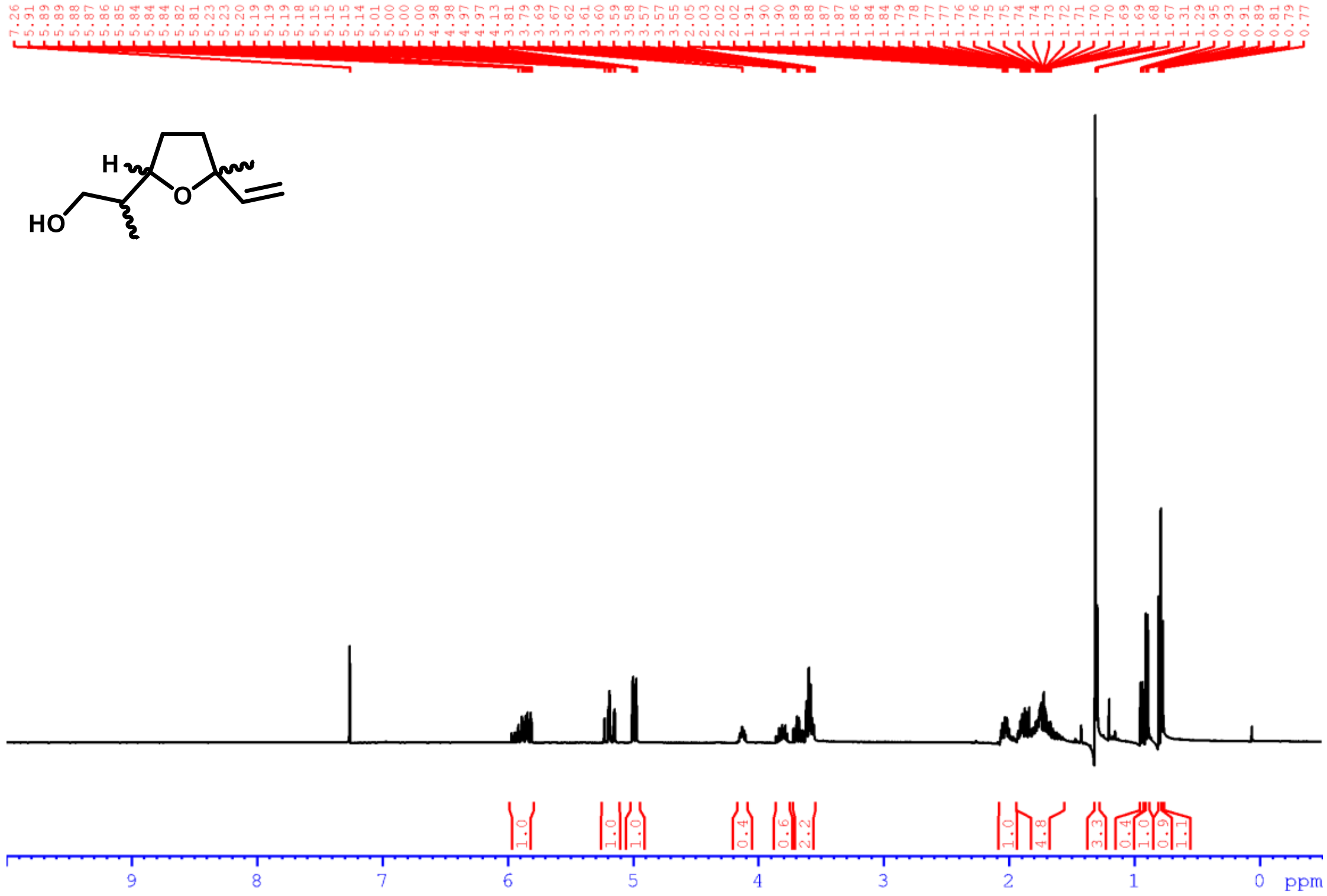

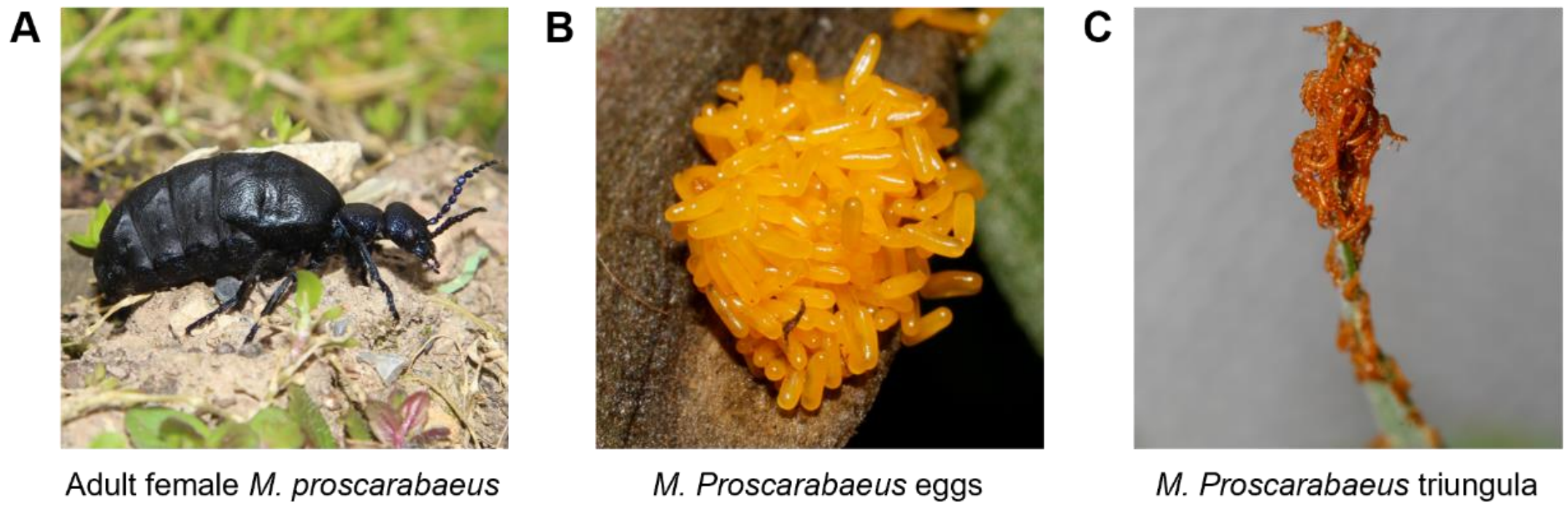

Supplementary Fig. 1. Collection and cultivation of *M. proscarabaeus* triungula.

(**A**) Wild adult female *M*. *proscarabaeus* collected in the field (Feb–Apr, 2024–25, Jena, Thuringia, Germany); (**B**) *M*. *proscarabaeus* eggs; (**C**) newly hatched *M*. *proscarabaeus* triungula aggregating on wheatgrass.

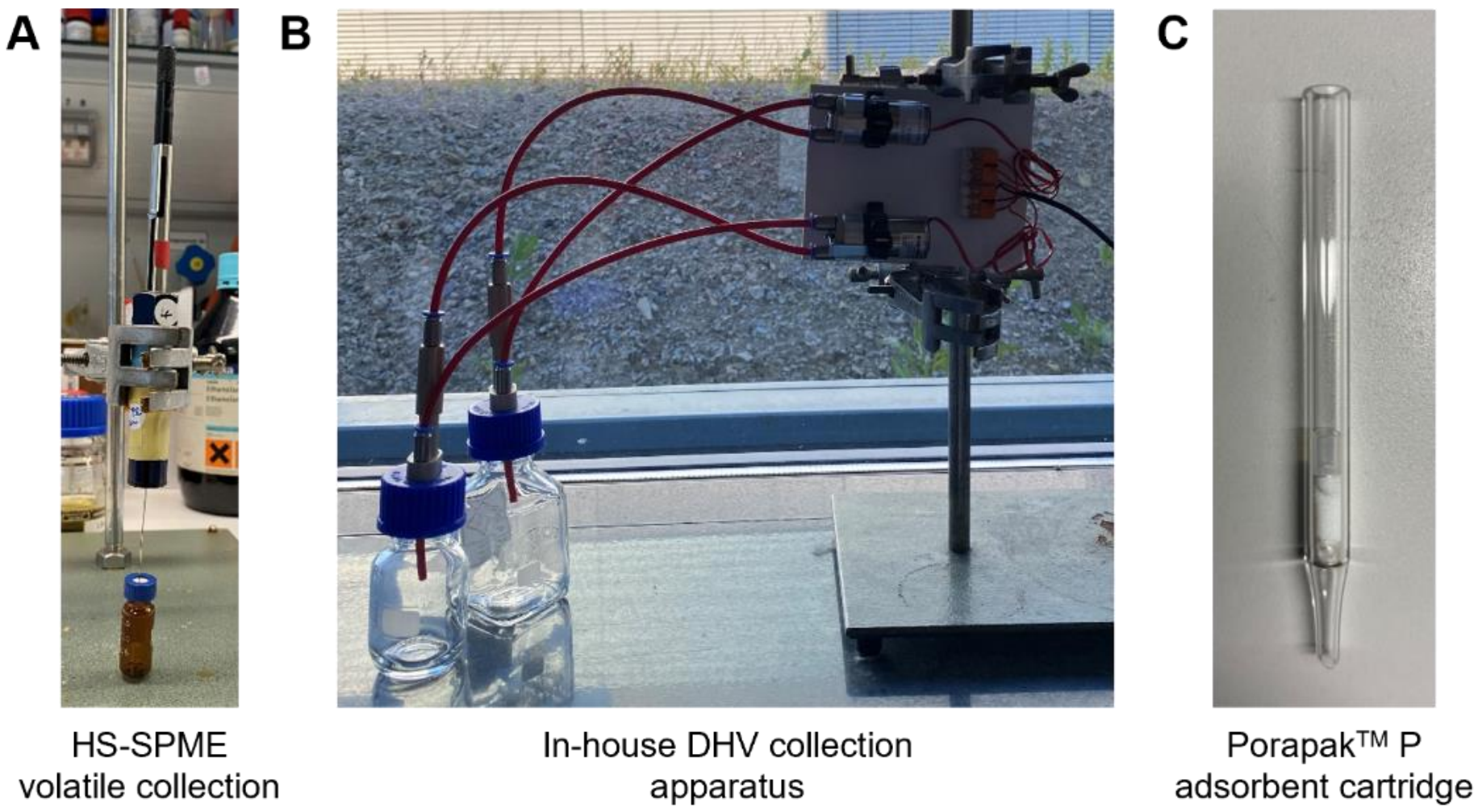

Supplementary Fig. 2. Triungulin volatile collection.

(**A**) Triungulin volatiles were collected using HS-SPME or (**B**) with an in-house dynamic headspace volatile (DHV) collection sampling apparatus fitted with a (**C**) removable Porapak™ P adsorbent cartridge.

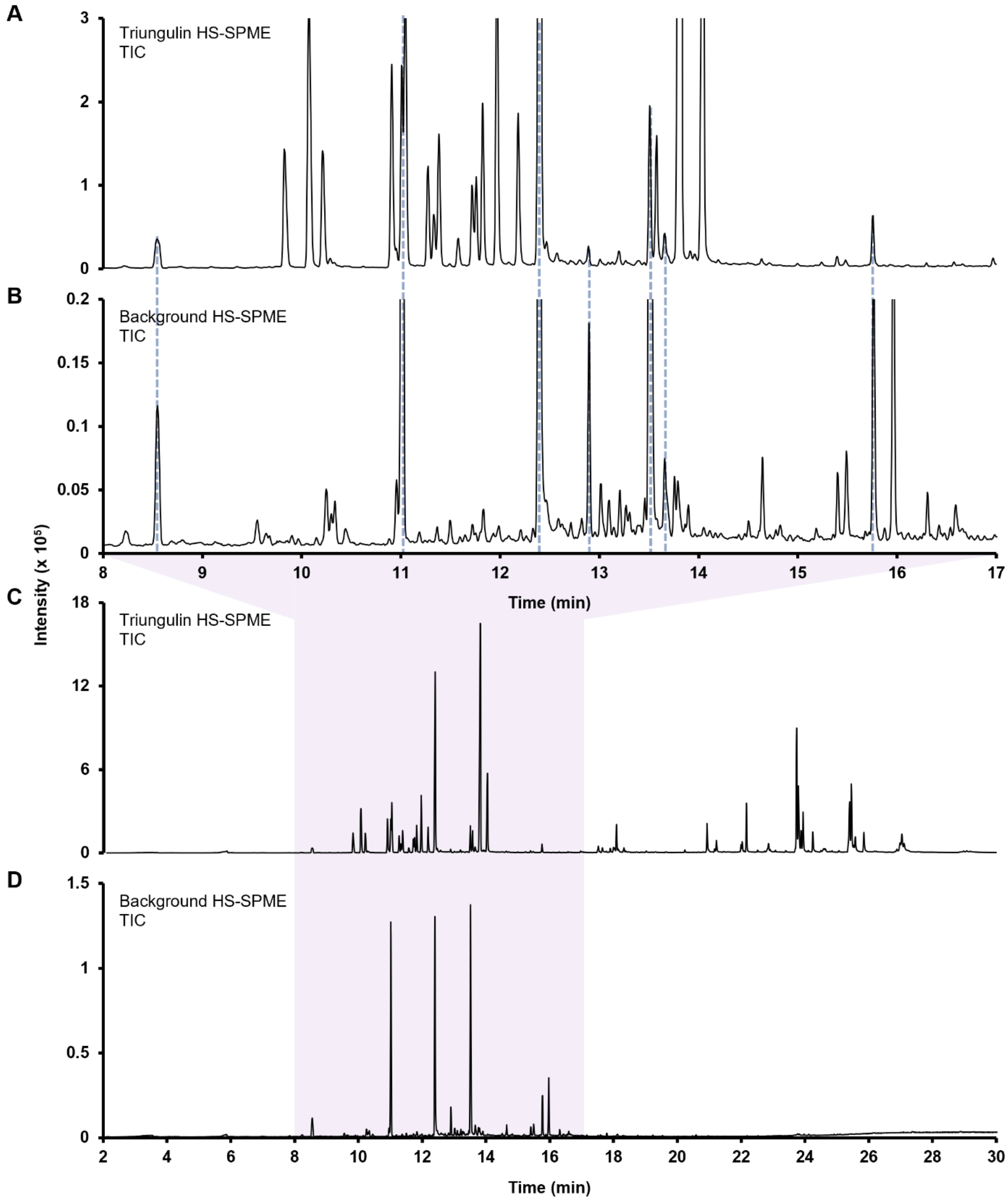

Supplementary Fig. 3. Comparison of HS-SPME GC-MS volatile profile of triungula with background.

(**A**) Expansion of GC-MS total ion chromatogram (TIC) of triungulin volatile profile. (**B**) Expanded TIC obtained for background. (**C**) Complete TIC for triungula showing over thirty distinct C_{19}–C_{27}

saturated and unsaturated long-chain hydrocarbons that were tentatively identified using the National Institute of Standards and Technology (NIST) MS-Library v. 3.0 (2023). (**D**) Full TIC for background. Separation method: A.

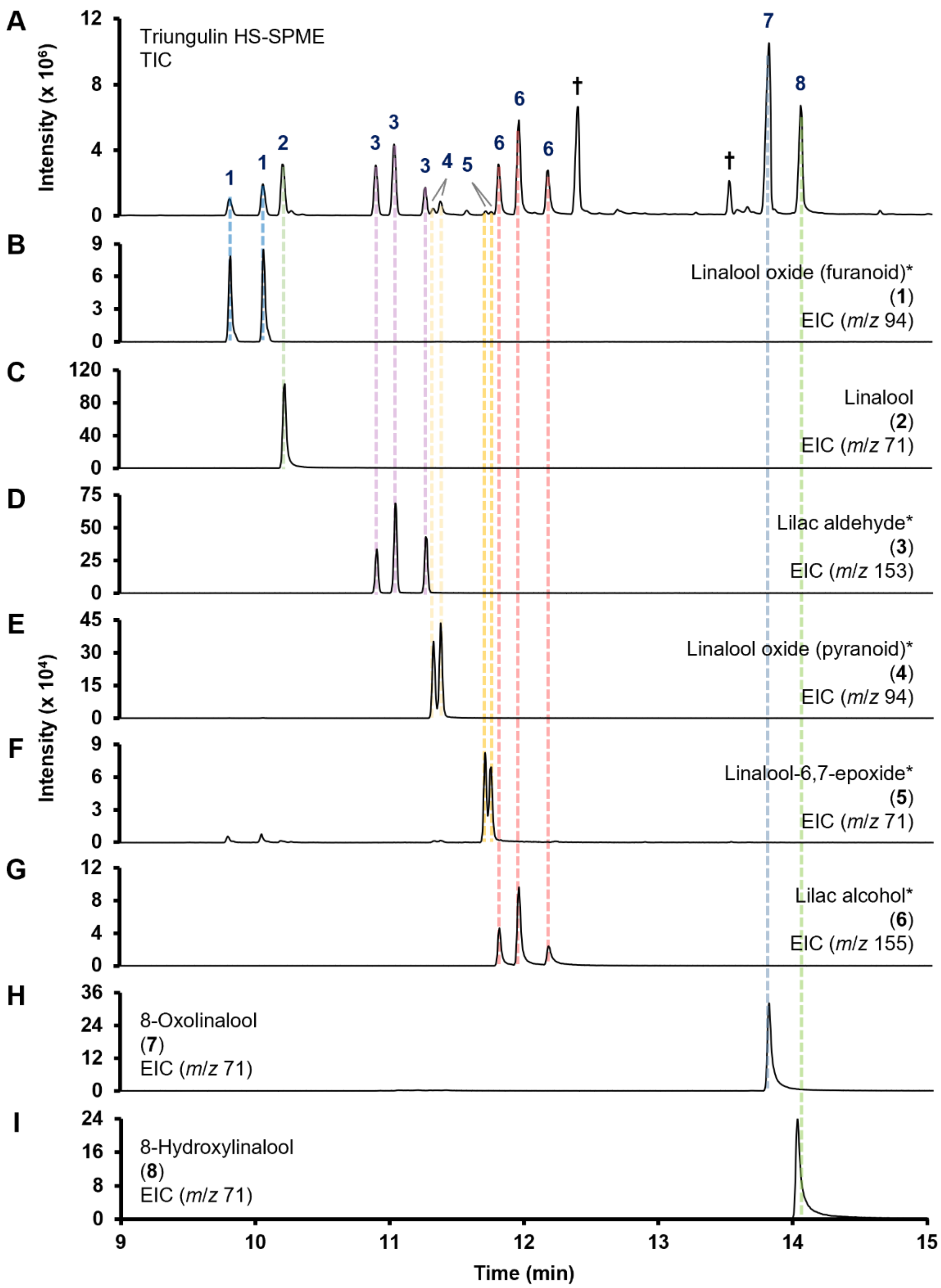

Supplementary Fig. 4. Identification of triungulin monoterpenoid volatiles.

Comparison of GC-MS chromatographic data obtained for (**A**) HS-SPME of triungula, (**B**) linalool oxide (furanoid) (**1**)*, (**C**) linalool (**2**), (**D**) lilac aldehyde (**3**)*, (**E**) linalool oxide (pyranoid) (**4**)*, (**F**) linalool-6,7-epoxide (**5**)*, (**G**) lilac alcohol (**6**)*, (**H**) 8-oxolinalool (**7**), and (**I**) 8-hydroxylinalool (**8**). Separation method: A. *Mixture of stereoisomers. †Background.

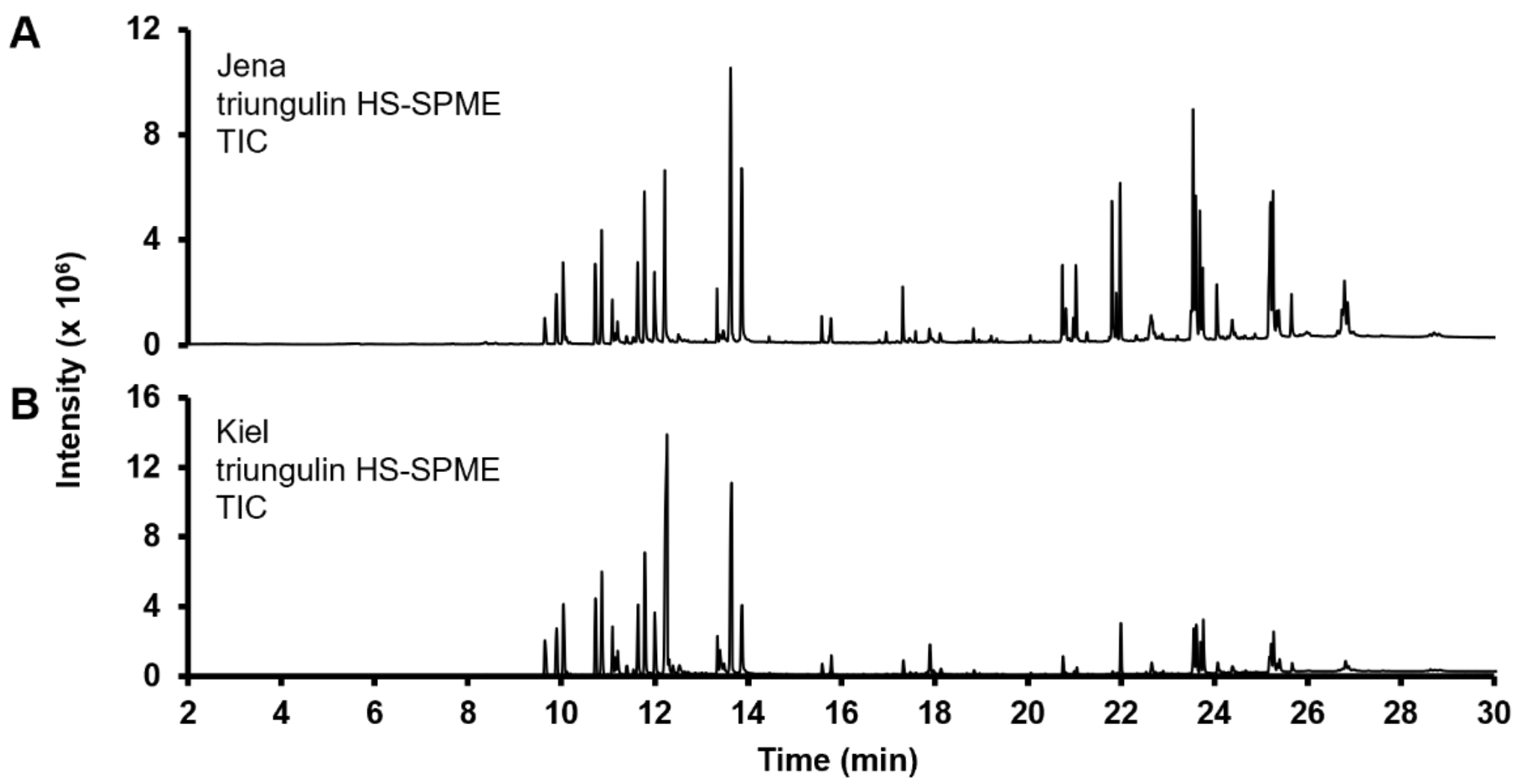

Supplementary Fig. 5. Comparison of HS-SPME GC-MS data for allopatric populations of *M. proscarabaeus* larvae.

GC-MS TIC data obtained for two geographically-distinct triungulin populations found in (**A**) Jena and (**B**) Knoop/Kiel showed no notable difference in monoterpenoid bouquet composition. Separation method: A.

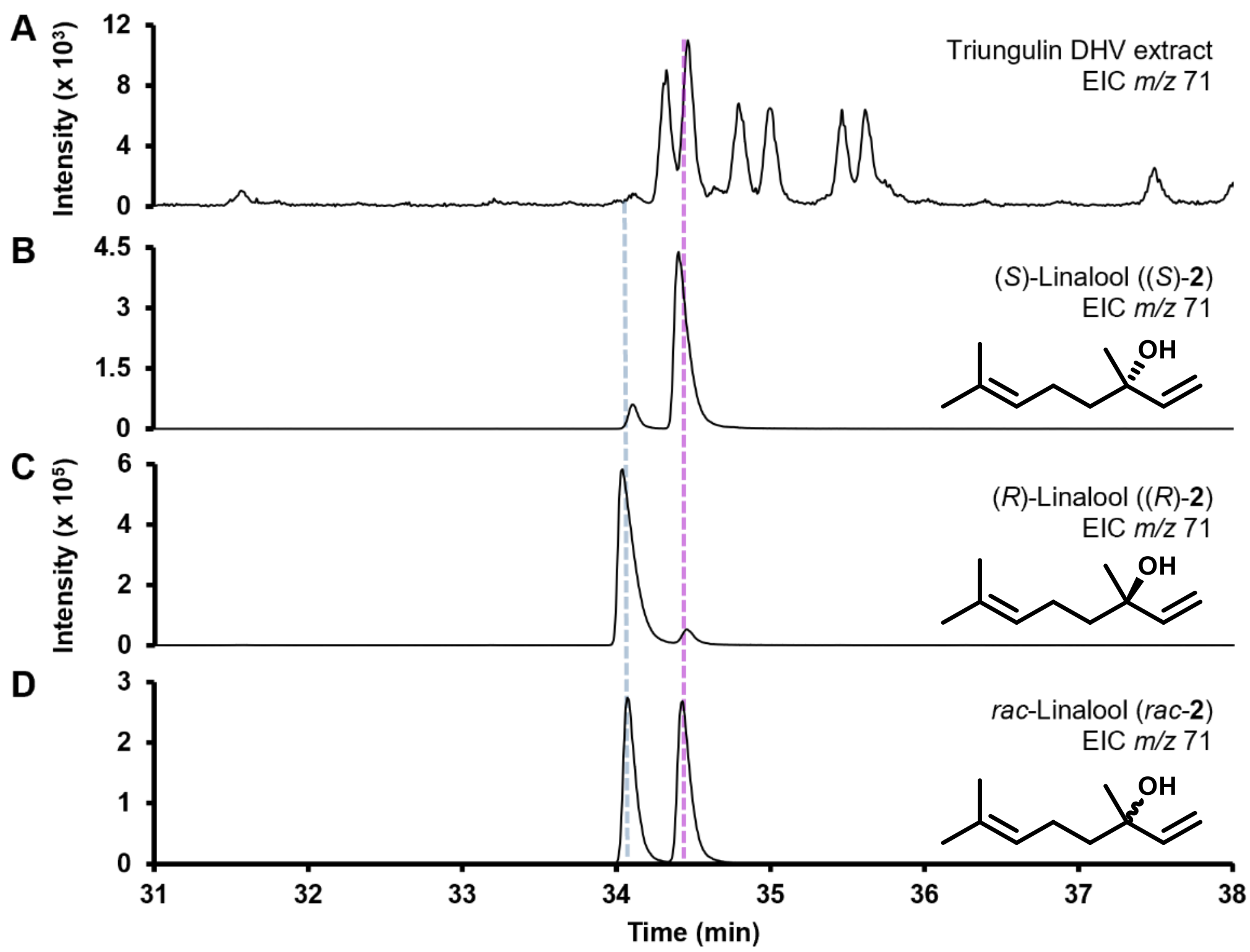

Supplementary Fig. 6. Triungula exclusively emit (*S*)-linalool ((*S*)-2).

Comparison of GC-MS chromatographic data obtained for (**A**) triungula DHV collection extract, (**B**) (*S*)-**2**, (**C**) (*R*)-**2**, and (**D**) *rac*-**2**. Separation method: B.

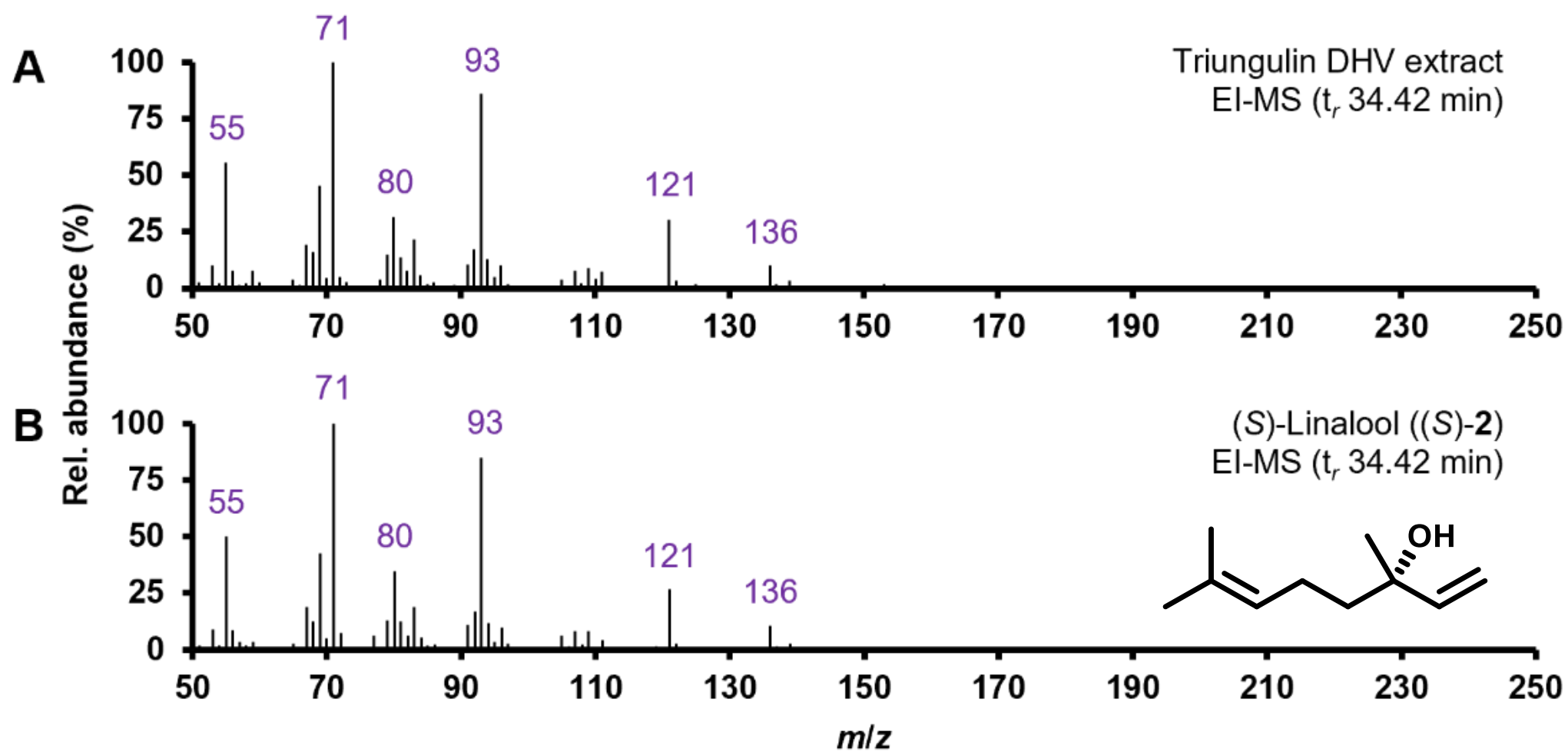

Supplementary Fig. 7. EI-MS spectra confirm peak identity as (*S*)-linalool ((*S*)-2).

Comparison of EI-MS data obtained for (**A**) triungulin DHV collection extract peak (t_r 34.42 min, **Supplementary Fig. 6**) and (**B**) (*S*)-**2** reference standard.

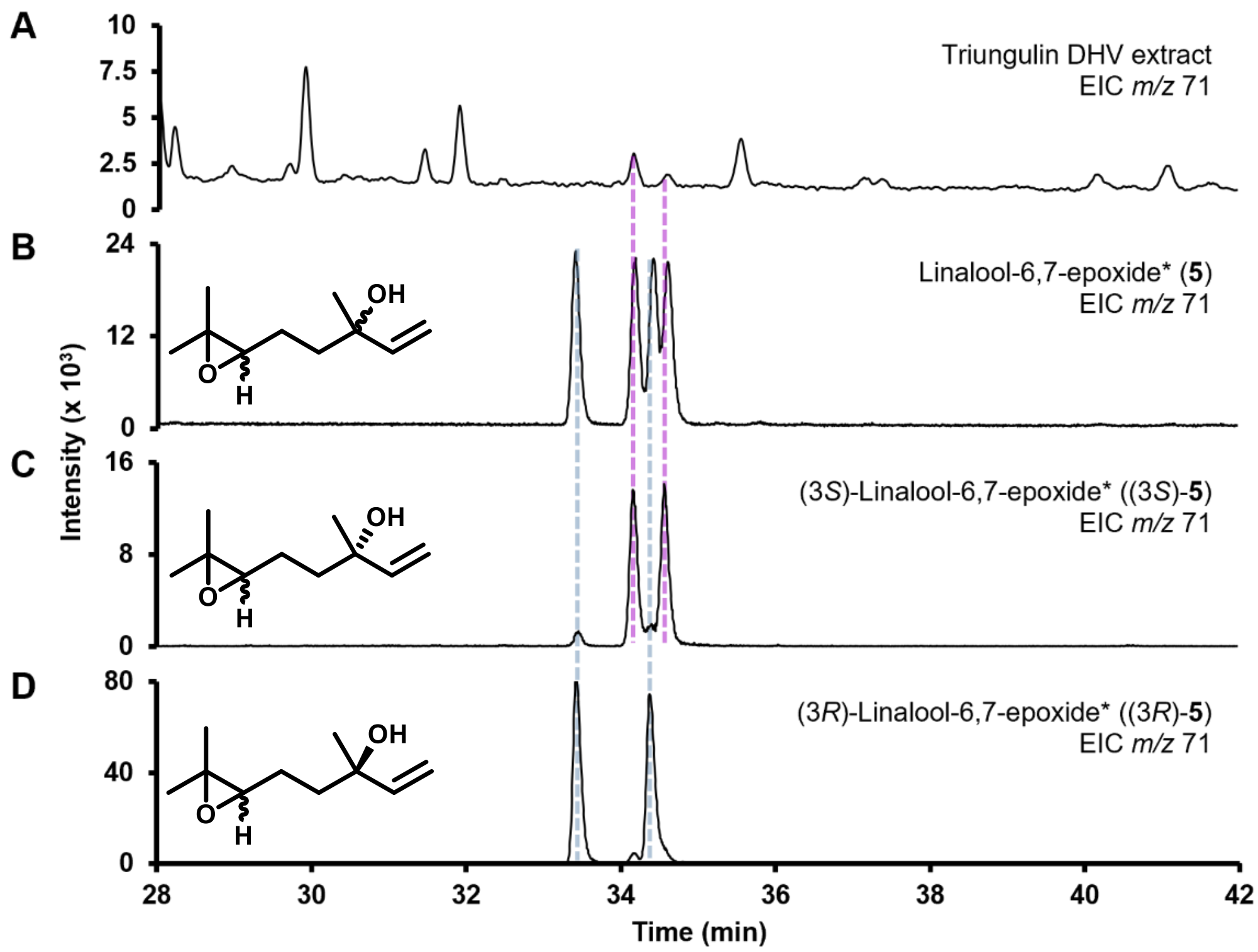

Supplementary Fig. 8. Triungula emit both stereoisomers of (3*S*)-linalool-6,7-epoxide.

Comparison of GC-MS chromatographic data obtained for (**A**) triungula DHV collection extract, (**B**) linalool-6,7-epoxide (**5**), (**C**) (3*S*)-**5**, and (**D**) (3*R*)-**5**. Separation method: C. *Mixture of stereoisomers.

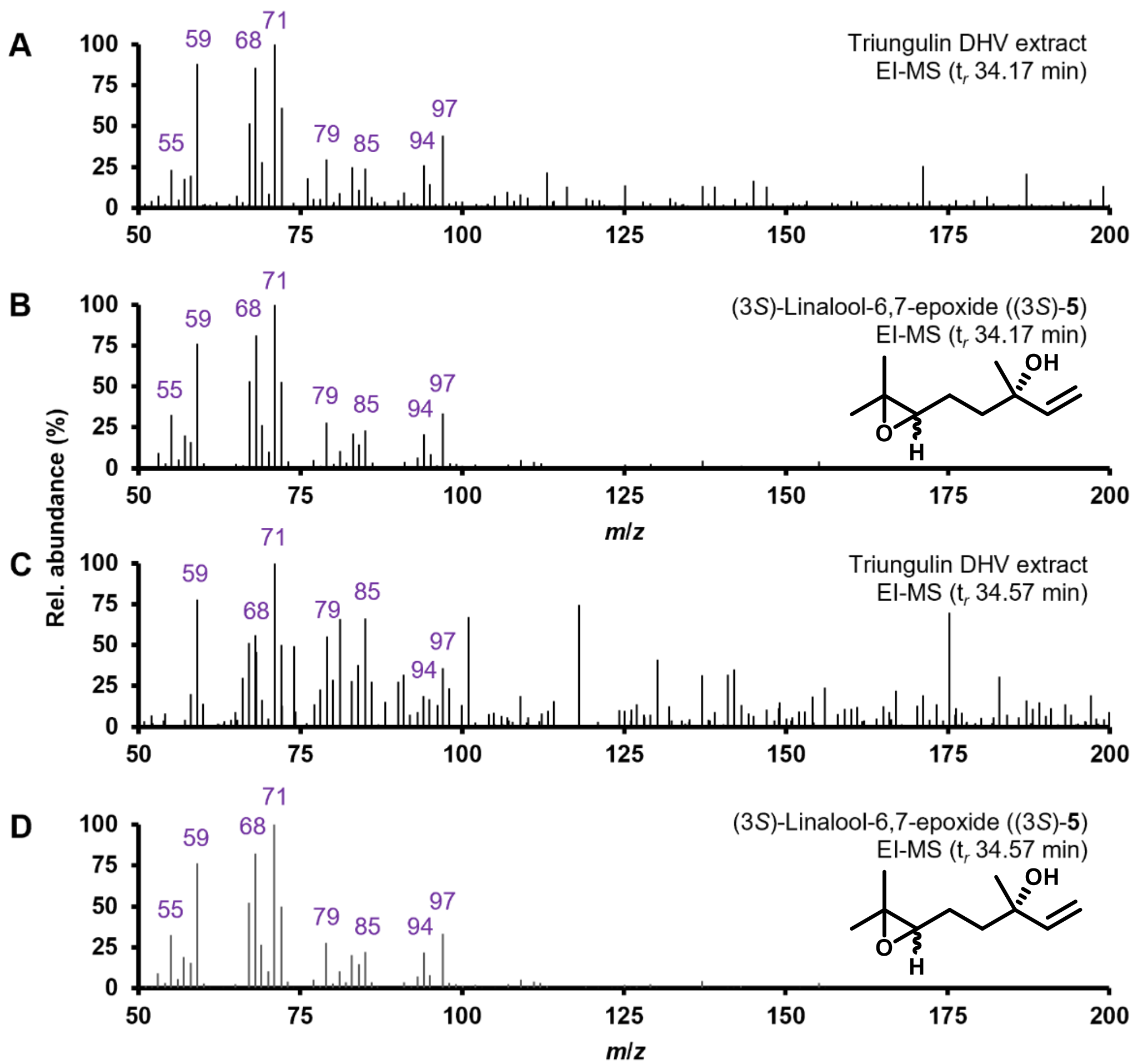

Supplementary Fig. 9. EI-MS spectra confirm peak identities as both stereoisomers of (3*S*)-linalool-6,7-epoxide.

Comparison of EI-MS data obtained for (**A**) triungulin DHV collection extract peak (t_r 34.17 min, **Supplementary Fig. 8**), (**B**) (3*S*)-**5** reference standard (t_r 34.17 min, **Supplementary Fig. 8**), (**C**) triungulin DHV collection extract peak (t_r 34.57 min, **Supplementary Fig. 8**), and (**D**) (3*S*)-**5** reference standard (t_r 34.57 min, **Supplementary Fig. 8**).

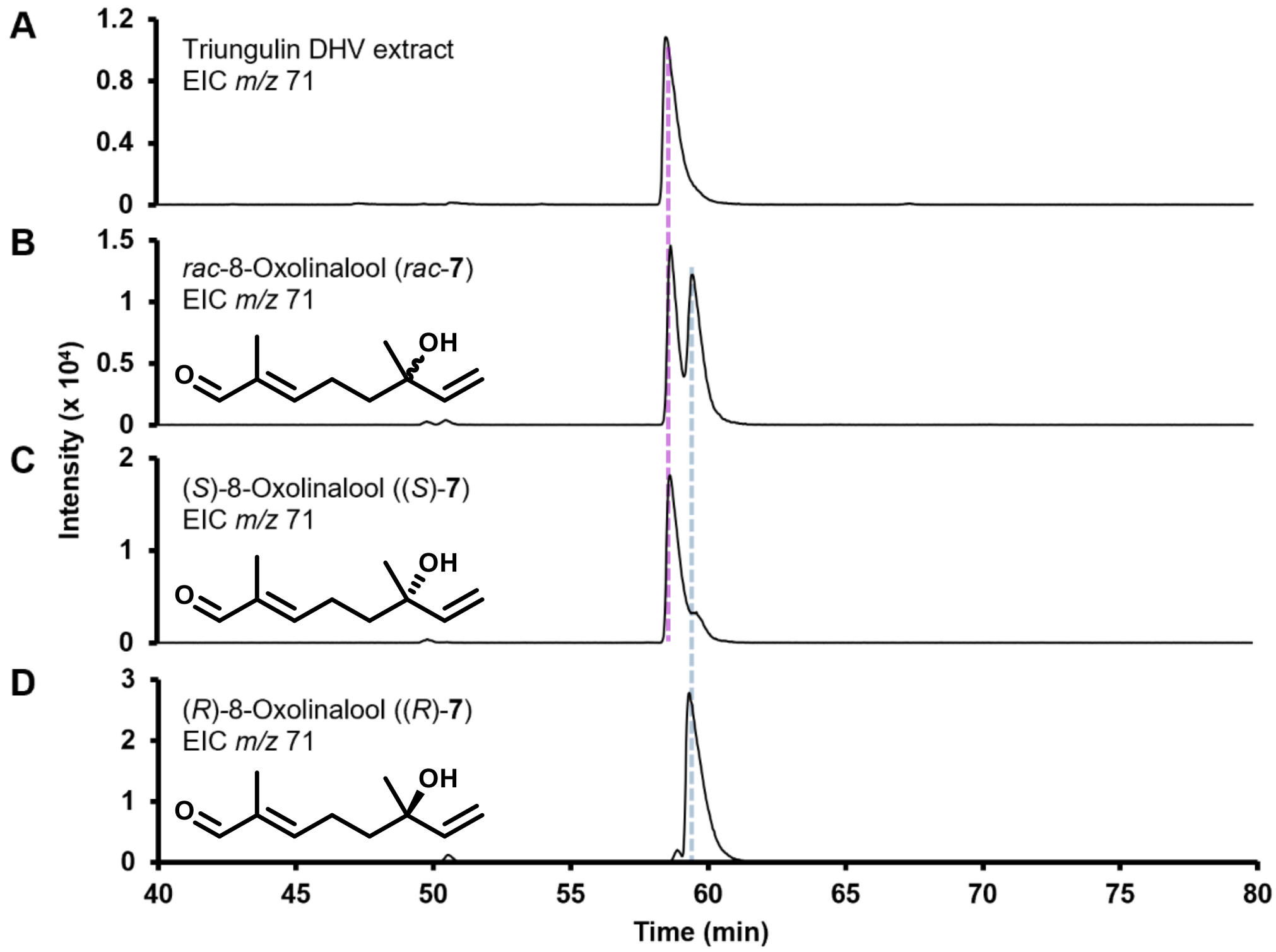

Supplementary Fig. 10. Triungula emit (*S*)-8-oxolinalool ((*S*)-7).

Comparison of GC-MS chromatographic data obtained for (**A**) triungula DHV collection extract, (**B**) *rac*-8-oxolinalool (*rac*-**7**), (**C**) (*S*)-**7**, and (**D**) (*R*)-**7**. Separation method: F.

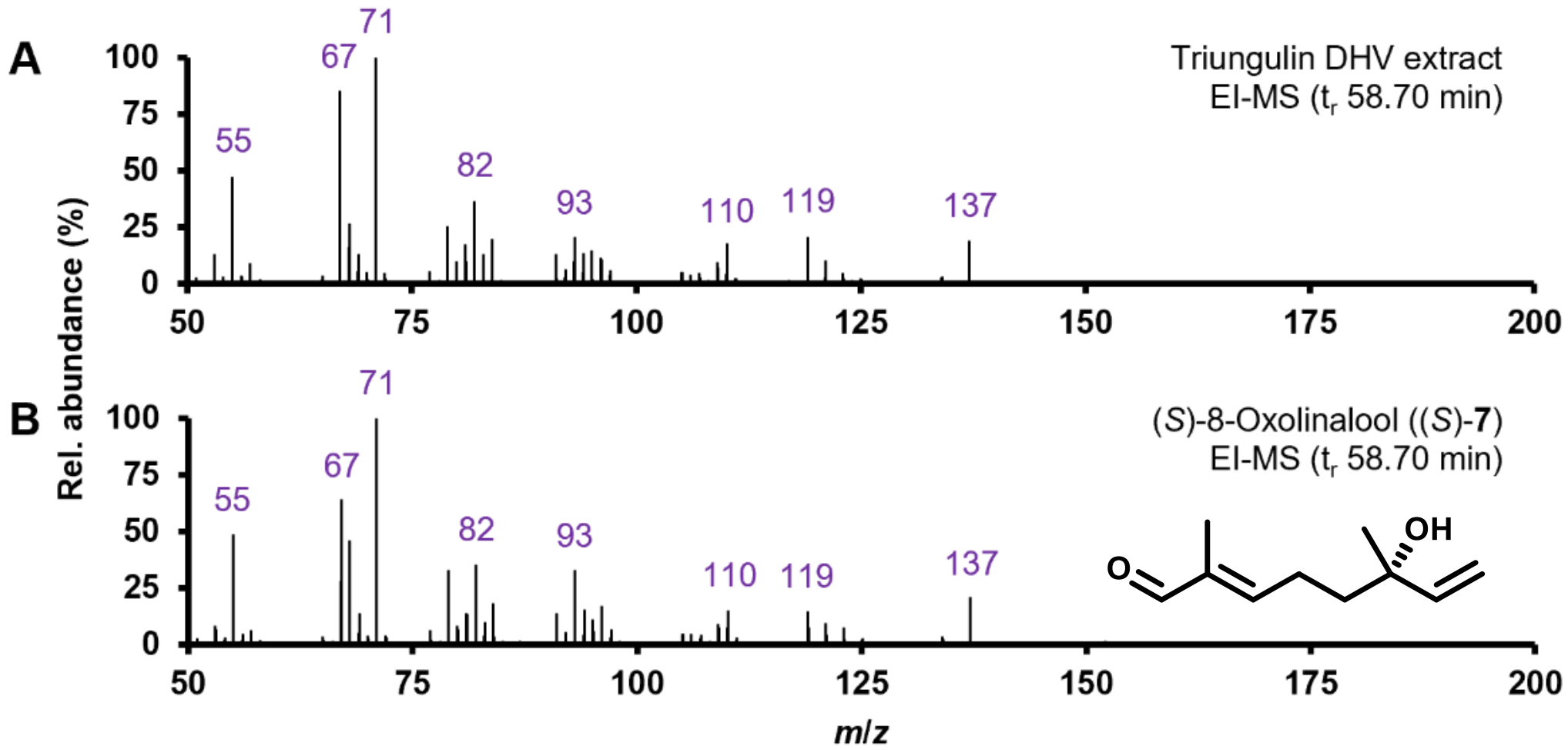

Supplementary Fig. 11. EI-MS spectra confirm peak identity as (*S*)-8-oxolinalool ((*S*)-7).

Comparison of EI-MS data obtained for (**A**) triungulin DHV collection extract peak (t_r 58.70 min, **Supplementary Fig. 10**) and (**B**) (*S*)-**7** reference standard.

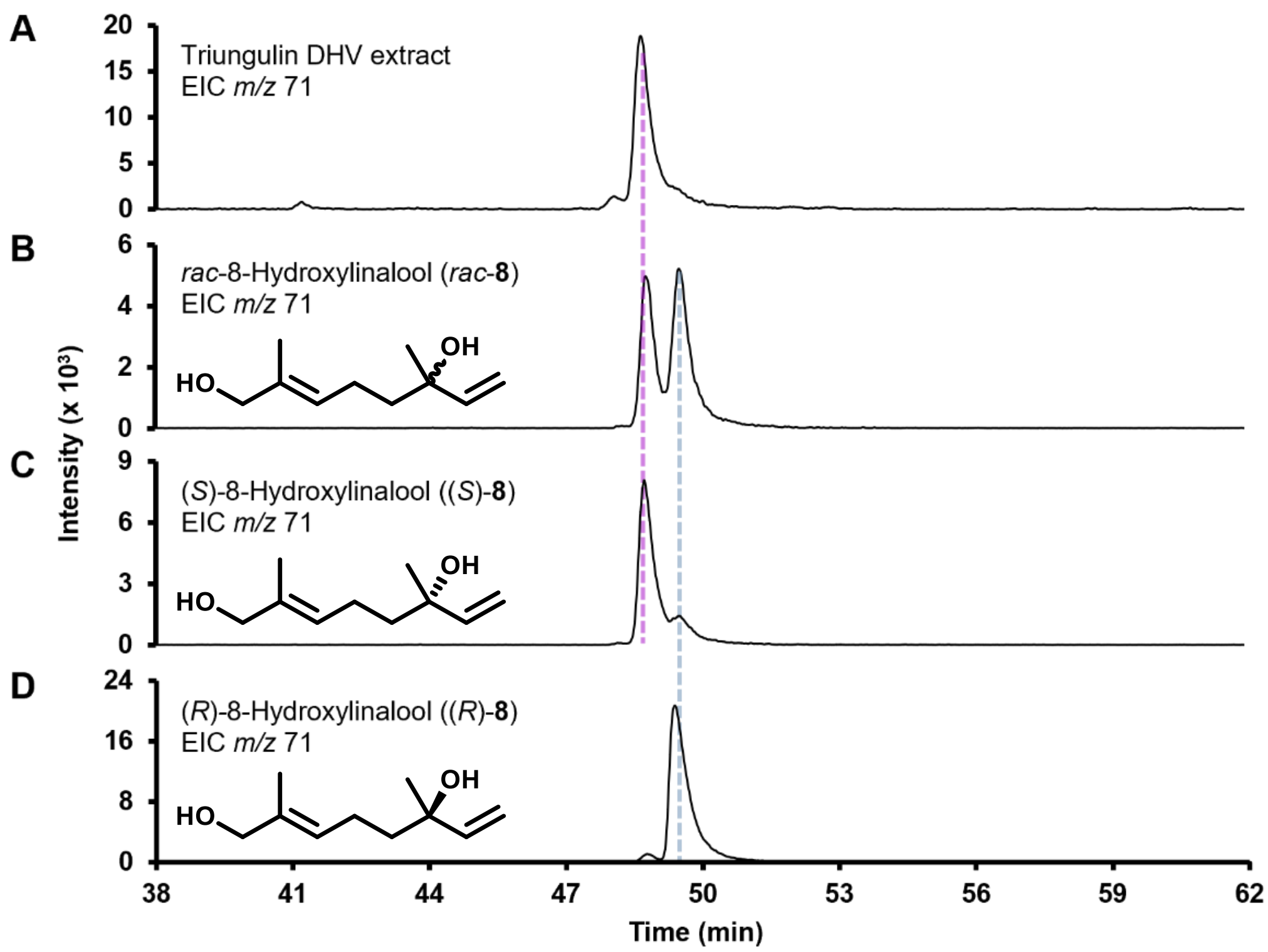

Supplementary Fig. 12. Triungula emit (*S*)-8-hydroxylinalool ((*S*)-8).

Comparison of GC-MS chromatographic data obtained for (**A**) triungula DHV collection extract, (**B**) *rac*-8-hydroxylinalool (*rac*-**8**), (**C**) (*S*)-**8**, and (**D**) (*R*)-**8**. Separation method: E.

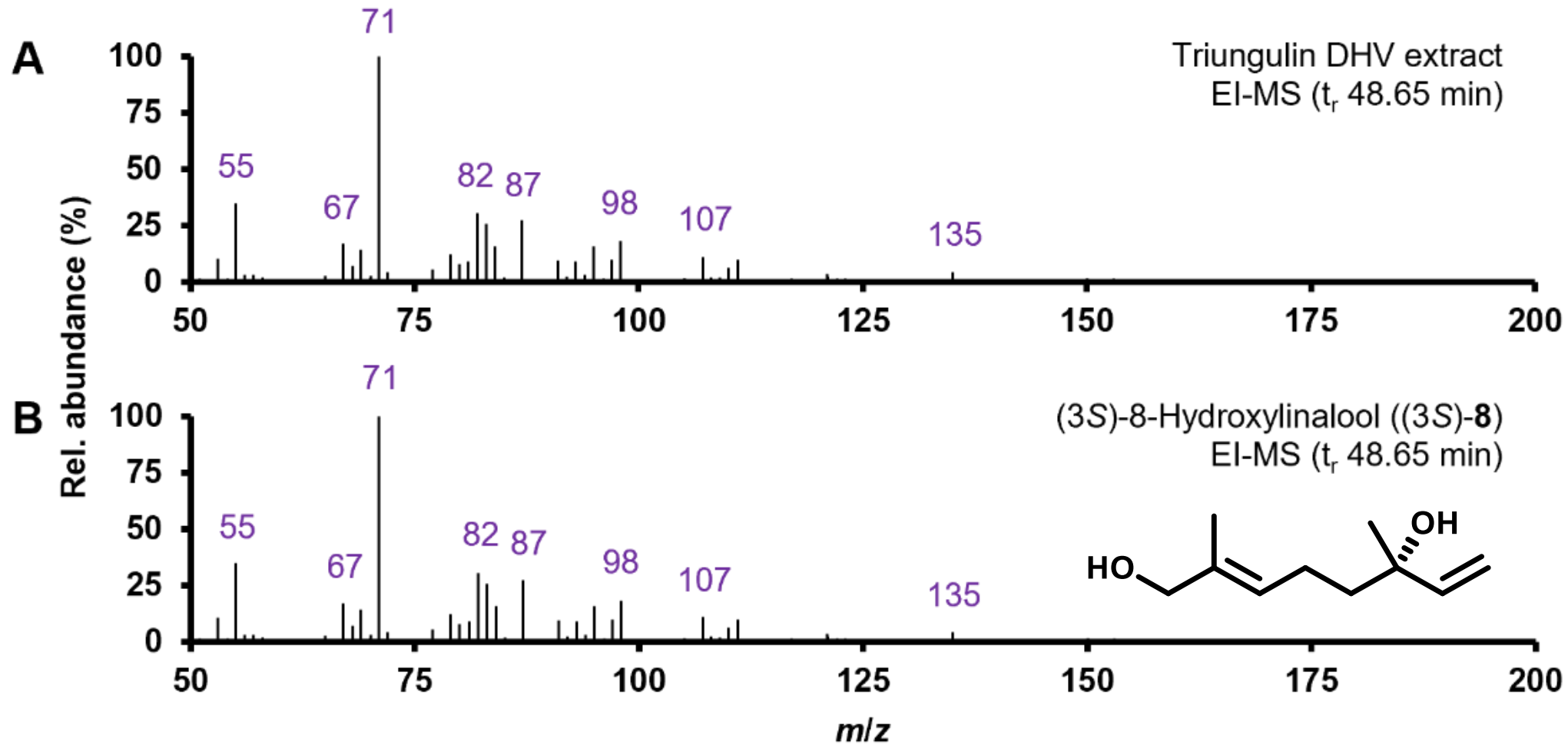

Supplementary Fig. 13. EI-MS spectra confirm peak identity as (*S*)-8-hydroxylinalool ((*S*)-8).

Comparison of EI-MS data obtained for (**A**) triungulin DHV collection extract peak (t_r 48.65 min, **Supplementary Fig. 12**) and (**B**) (*S*)-**8** reference standard.

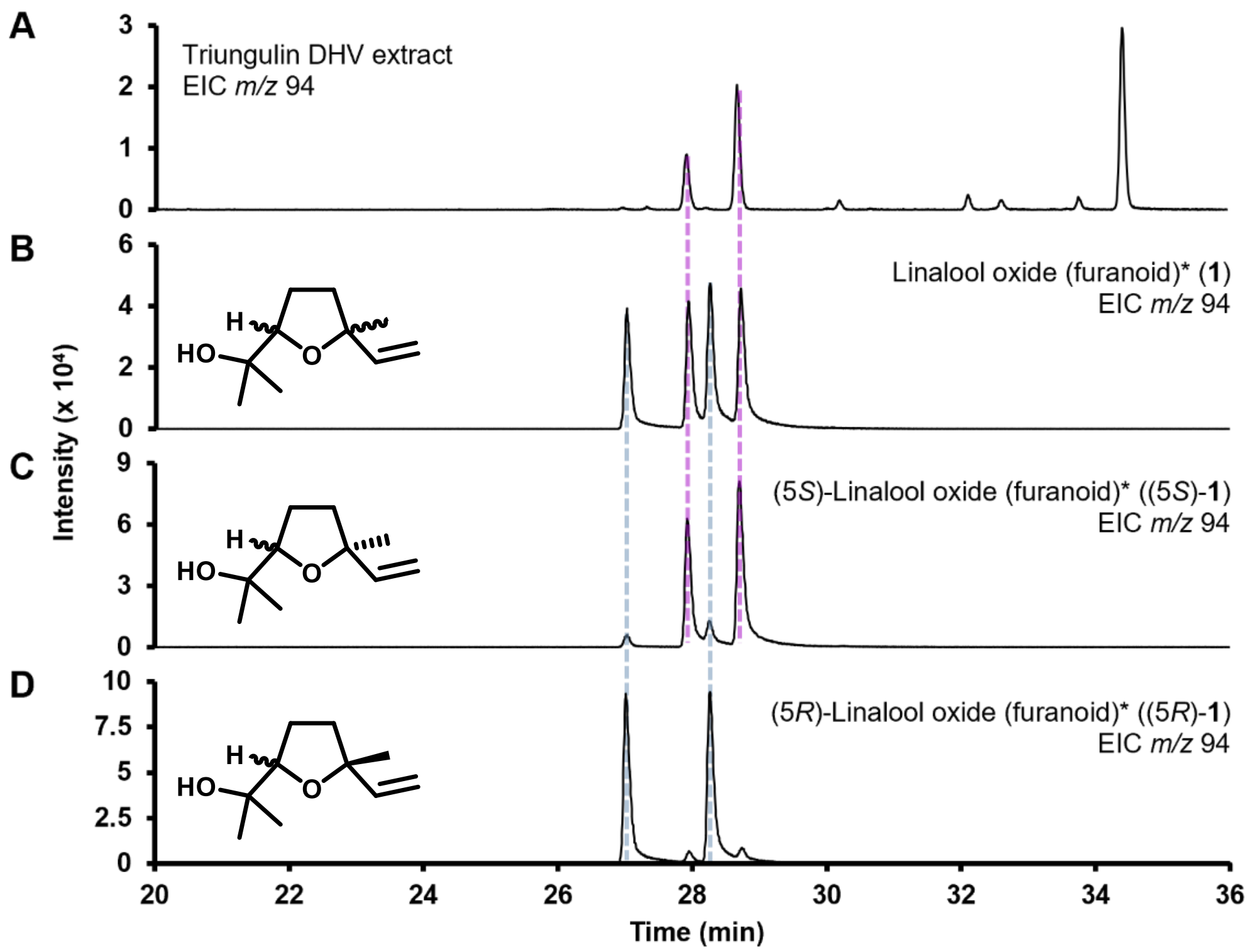

Supplementary Fig. 14. Triungula emit (2*S*,5*S*)- and (2*R*,5*S*)-linalool oxide (furanoid) ((5*S*)-1).

Comparison of GC-MS chromatographic data obtained for (**A**) triungula DHV collection extract, (**B**) linalool oxide (furanoid) (**1**), (**C**) (5*S*)-**1**, and (**D**) (5*R*)-**1**. Separation method: D. *Mixture of stereoisomers.

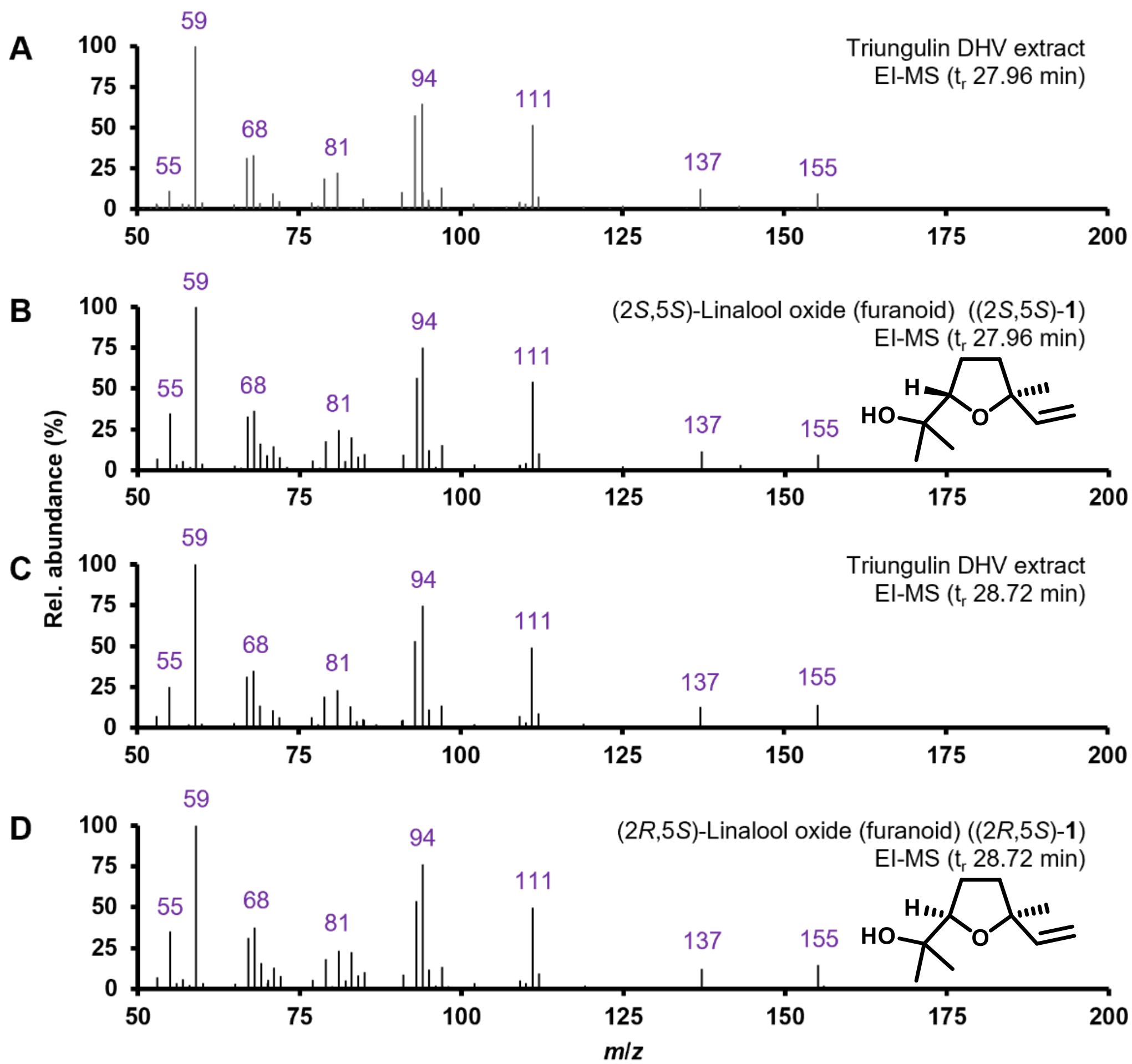

Supplementary Fig. 15. EI-MS spectra confirm peak identities as both stereoisomers of (5*S*)-linalool oxide (furanoid) ((5*S*)-1).

Comparison of EI-MS data obtained for (**A**) triungulin DHV collection extract peak (t_r 27.96 min, **Supplementary Fig. 14**), (**B**) (2*S*,5*S*)-**1** (t_r 27.96 min, **Supplementary Fig. 14**), (**C**) triungulin DHV collection extract peak (t_r 28.72 min, **Supplementary Fig. 14**), and (**D**) (2*R*,5*S*)-**1** (t_r 28.72 min, **Supplementary Fig. 14**).

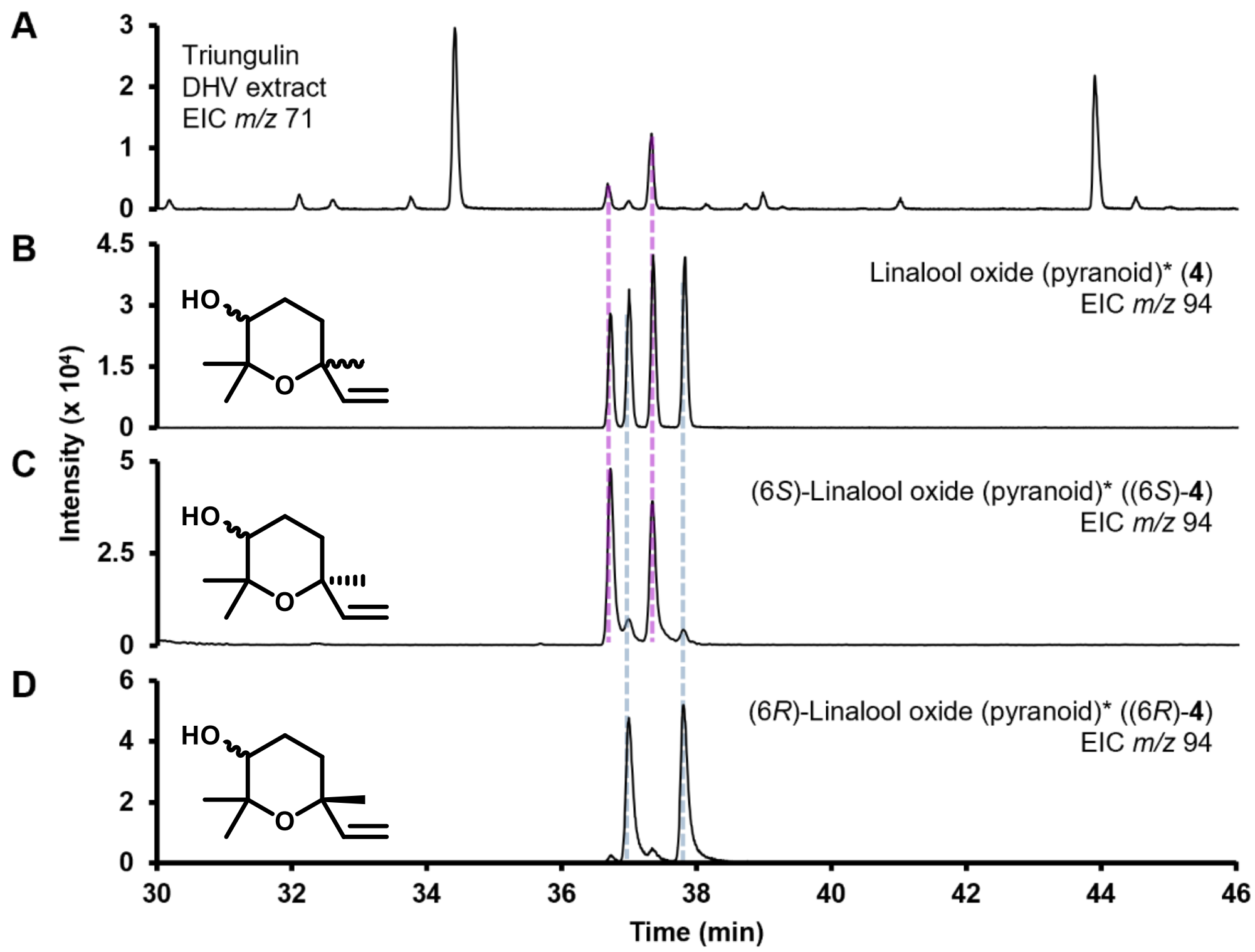

Supplementary Fig. 16. Triungula emit (3*R*,6*S*)- and (3*S*,6*S*)-linalool oxide (pyranoid) ((6*S*)-4).

Comparison of GC-MS chromatographic data obtained for (**A**) triungula DHV collection extract, (**B**) linalool oxide (pyranoid) (**4**), (**C**) (6*S*)-**4**, and (**D**) (6*R*)-**4**. Separation method: D. *Mixture of stereoisomers.

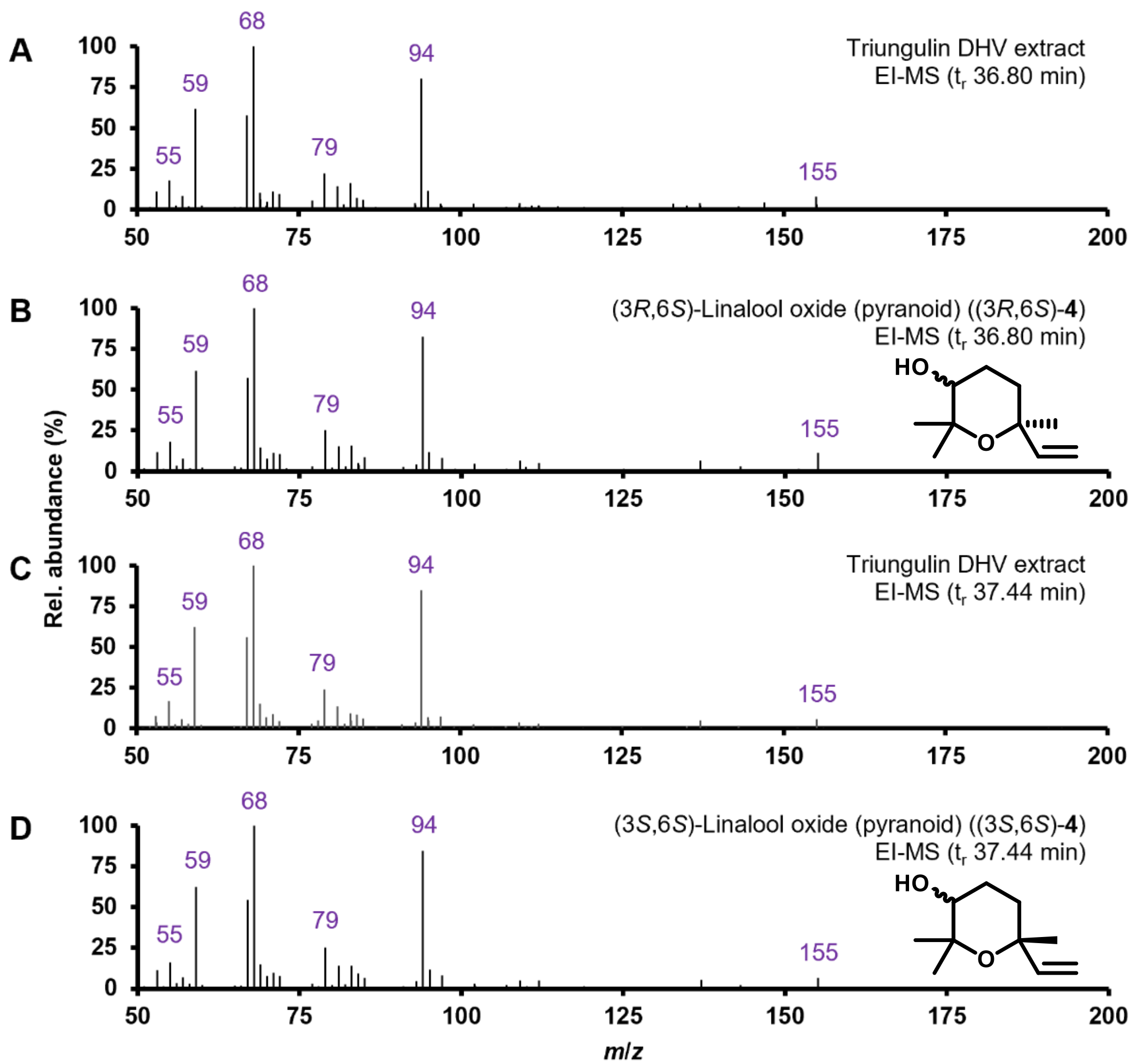

Supplementary Fig. 17. EI-MS spectra confirm peak identities as both stereoisomers of (6*S*)-linalool oxide (pyranoid).

Comparison of EI-MS data obtained for (**A**) triungulin DHV collection extract peak (t_r 36.80 min, **Supplementary Fig. 16**), (**B**) (3*R*,6*S*)-**4** (t_r 36.80 min, **Supplementary Fig. 16**), (**C**) triungulin DHV collection extract peak (t_r 37.44 min, **Supplementary Fig. 16**), and (**D**) (3*S*,6*S*)-**4** (t_r 37.44 min, **Supplementary Fig. 16**).

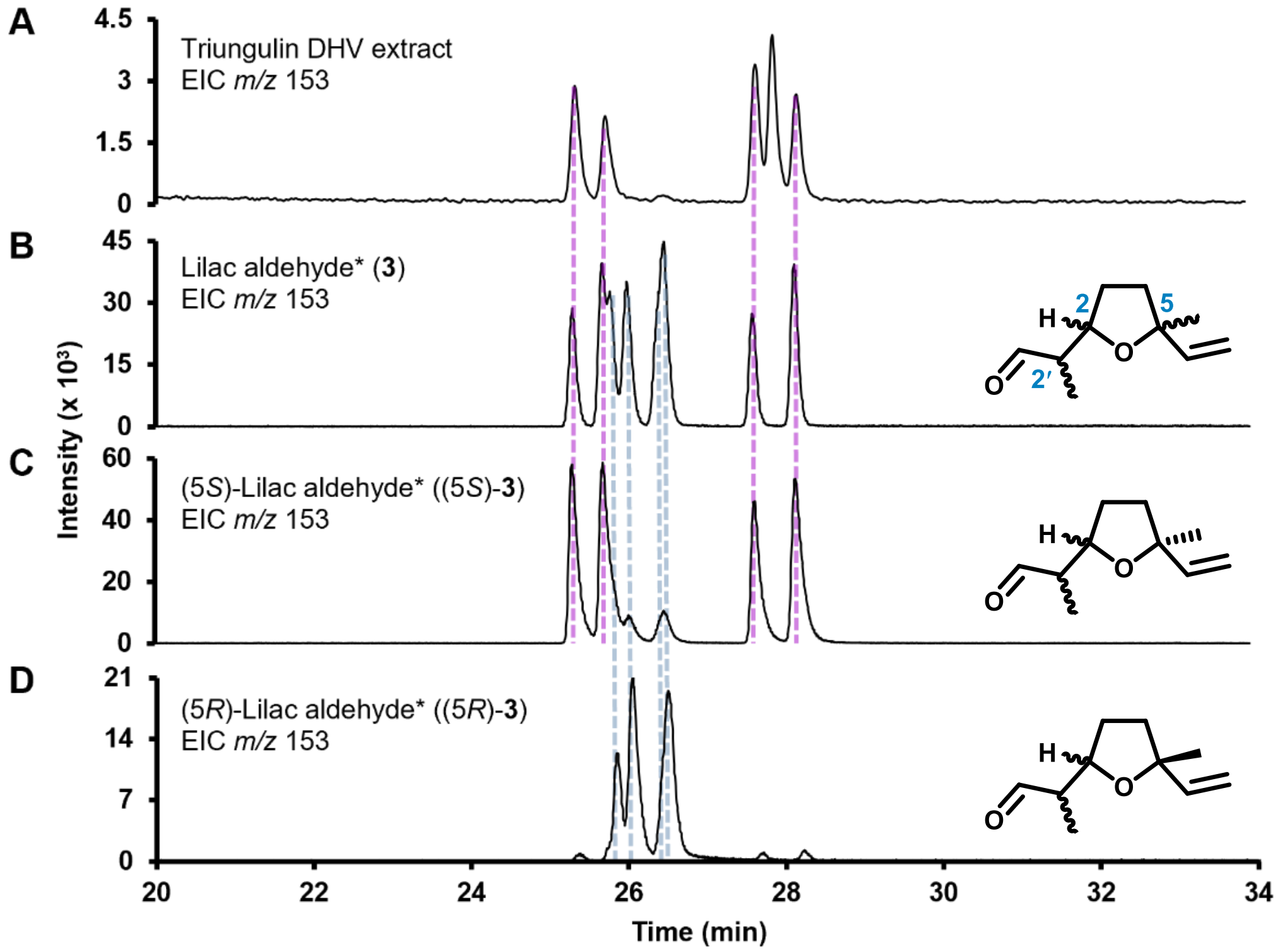

Supplementary Fig. 18. Triungula emit (2´*S*,2*S*,5*S*)-, (2´*R*,2*S*,5*S*)-, (2´*S*,2*R*,5*S*)-, and (2´*R*,2*R*,5*S*)-lilac aldehyde ((5*S*)-3).

Comparison of GC-MS chromatographic data obtained for (**A**) triungula DHV collection extract, (**B**) lilac aldehyde (**3**), (**C**) (5*S*)-**3**, and (**D**) (5*R*)-**3**. Separation method: C. *Mixture of stereoisomers.

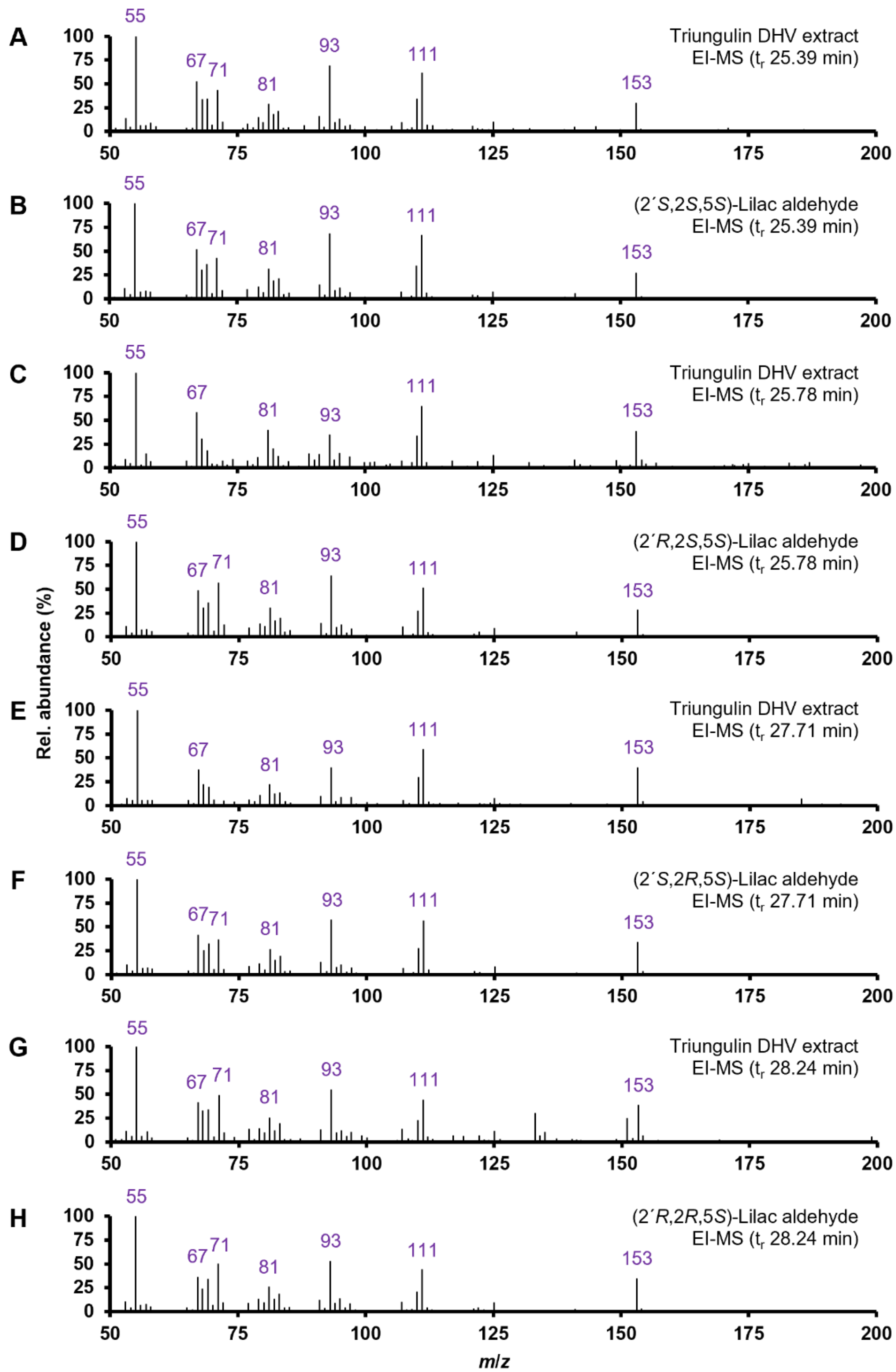

Supplementary Fig. 19. EI-MS spectra confirm peak identities of four stereoisomers of (5*S*)-lilac aldehyde ((5*S*)-3).

Comparison of EI-MS data obtained for (**A**) triungulin DHV collection extract peak (t_r 25.39 min, **Supplementary Fig. 18**), (**B**) (2´*S*,2*S*,5*S*)-**3** (t_r 25.39 min, **Supplementary Fig. 18**), (**C**) triungulin

DHV collection extract peak (t_r 25.78 min, **Supplementary Fig. 18**), (**D**) (2´*R*,2*S*,5*S*)-**3** (t_r 25.78 min, **Supplementary Fig. 18**), (**E**) triungulin DHV collection extract peak (t_r 27.71 min, **Supplementary Fig. 18**), (**F**) (2´*S*,2*R*,5*S*)-**3** (t_r 27.71 min, **Supplementary Fig. 18**), (**G**) triungulin DHV collection extract peak (t_r 28.24 min, **Supplementary Fig. 18**), and (**H**) (2´*R*,2*R*,5*S*)-**3** (t_r 28.24 min, **Supplementary Fig. 18**).

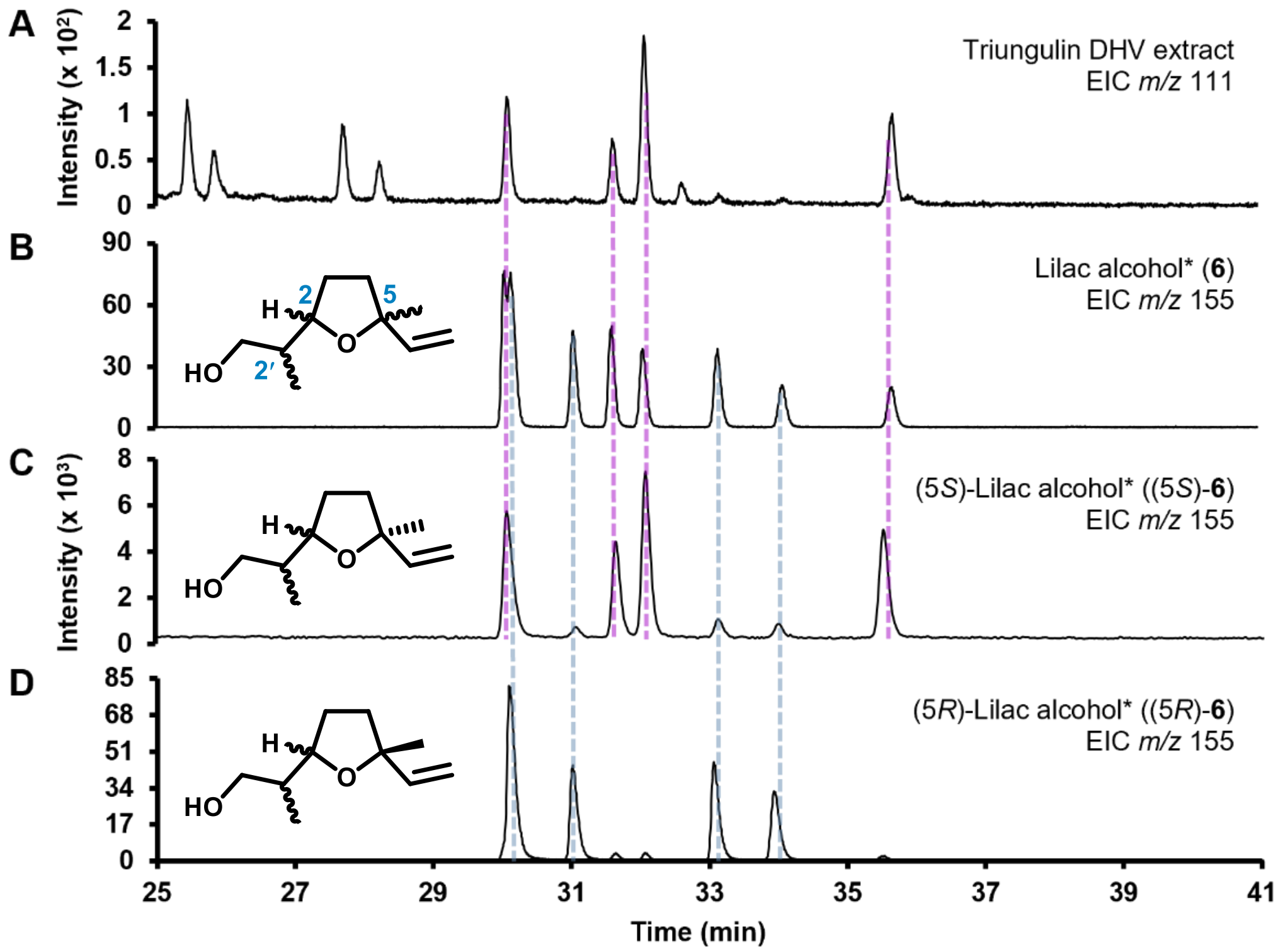

Supplementary Fig. 20. Triungula emit (2´*S*,2*S*,5*S*)-, (2´*S*,2*R*,5*S*)-, (2´*R*,2*S*,5*S*)-, and (2´*R*,2*R*,5*S*)-lilac alcohol (((5*S*)-6).

Comparison of GC-MS chromatographic data obtained for (**A**) triungula DHV collection extract, (**B**) lilac alcohol (**6**), (**C**) (5*S*)-**6**, and (**D**) (5*R*)-**6**. Separation method: C. *Mixture of stereoisomers.

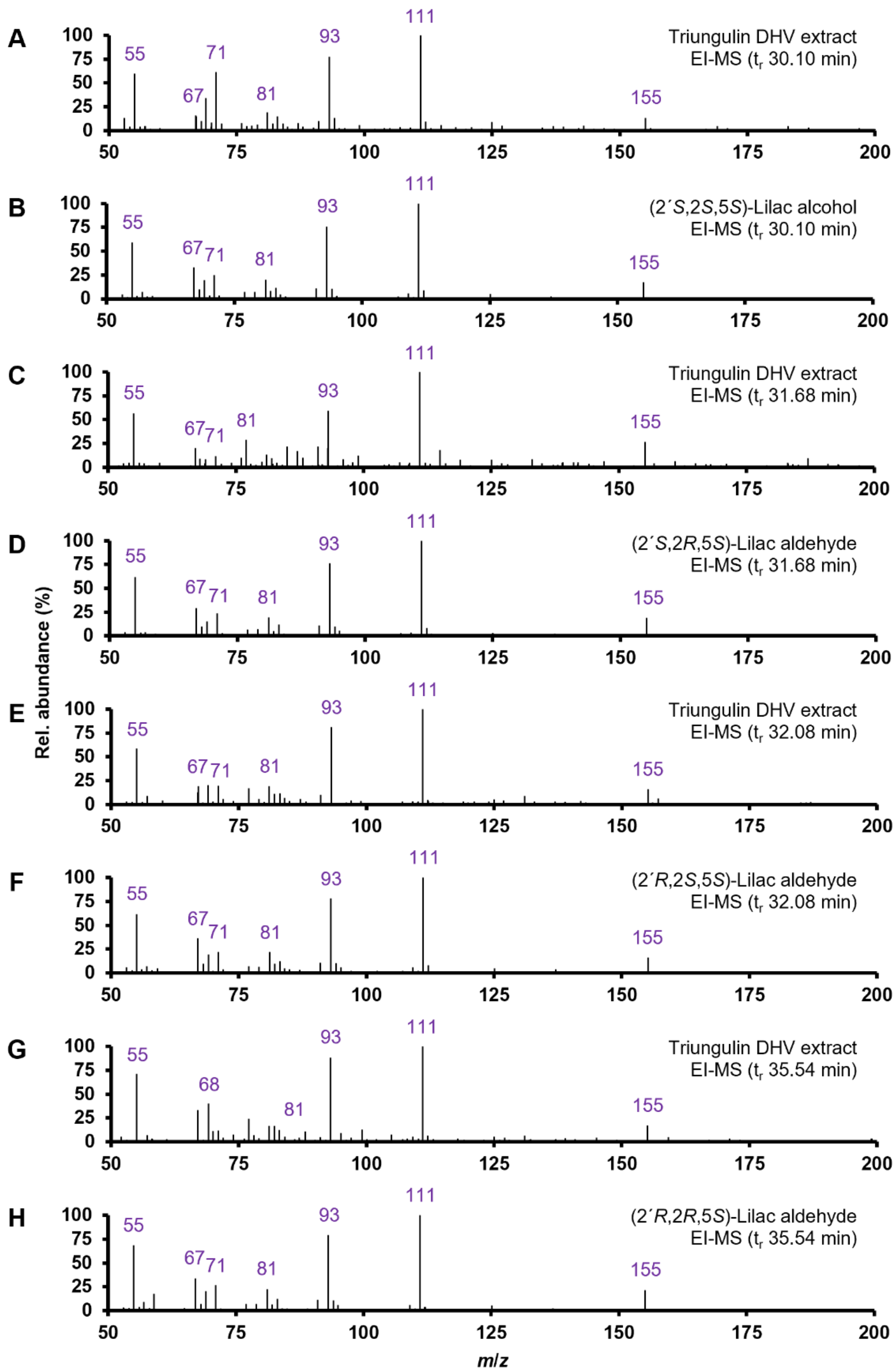

Supplementary Fig. 21. EI-MS spectra confirm peak identities of four stereoisomers of (5*S*)-lilac alcohol ((5*S*)-6).

Comparison of EI-MS data obtained for (**A**) triungulin DHV collection extract peak (t_r 30.10 min, **Supplementary Fig. 20**), (**B**) (2´*S*,2*S*,5*S*)-**6** (t_r 30.10 min, **Supplementary Fig. 20**), (**C**) triungulin

DHV collection extract peak (t_r 31.68 min, **Supplementary Fig. 20**), (**D**) (2´*S*,2*R*,5*S*)-**6** (t_r 31.68 min, **Supplementary Fig. 20**), (**E**) triungulin DHV collection extract peak (t_r 32.08 min, **Supplementary Fig. 20**), (**F**) (2´*R*,2*S*,5*S*)-**6** (t_r 32.08 min, **Supplementary Fig. 20**), (**G**) triungulin DHV collection extract peak (t_r 35.54 min, **Supplementary Fig. 20**), and (**H**) (2´*R*,2*R*,5*S*)-**3** (t_r 35.54 min, **Supplementary Fig. 20**).

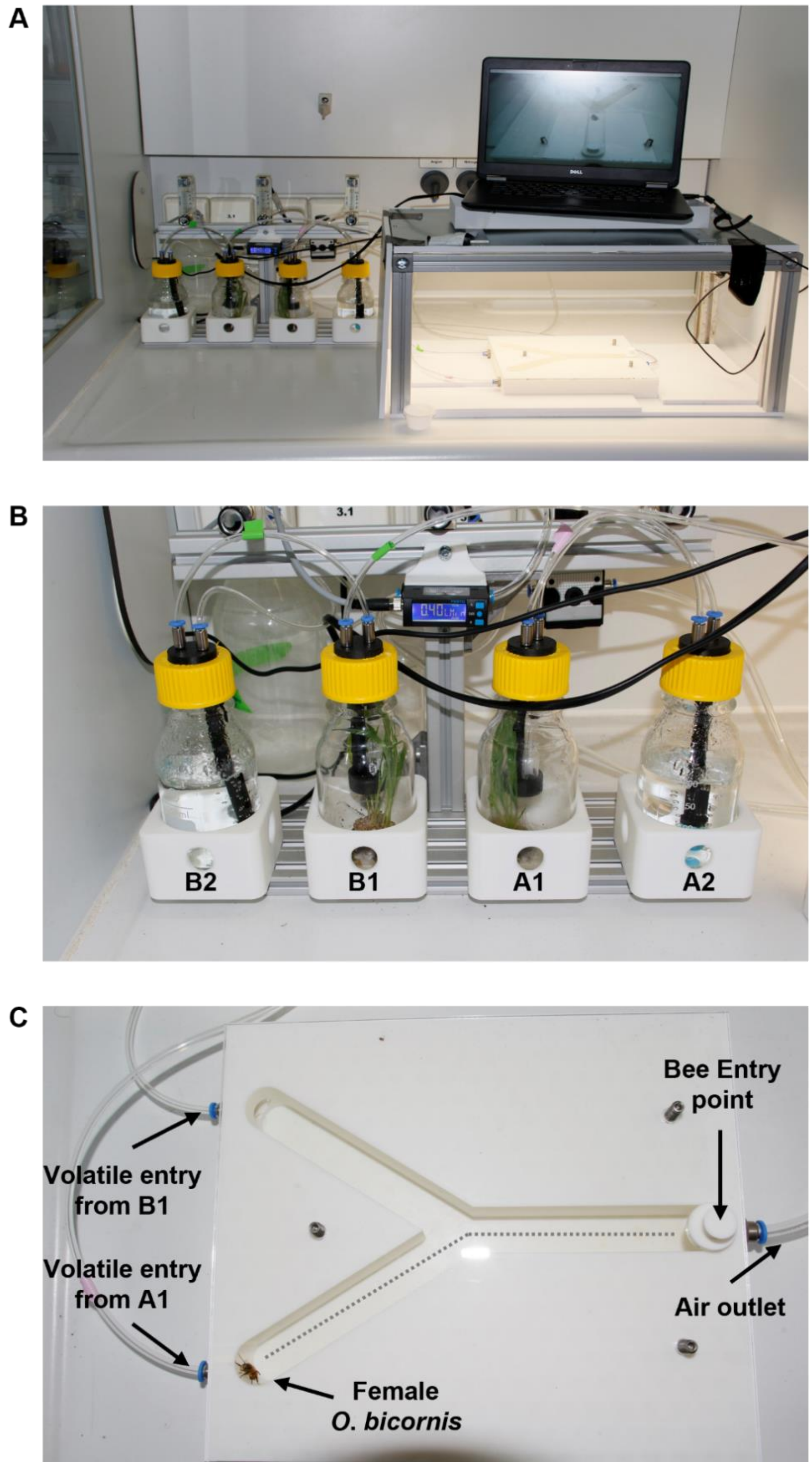

Supplementary Fig. 22. Y-tube dual-choice olfactometric assay setup.

(**A**) Dual-choice Y-tube assay setup. (**B**) Charcoal-filtered compressed air was first bubbled through distilled water (Bottles A2 or B2) and then passed over freshly sprouted *Triticum aestivum*

and a glass vial containing a solution of synthetic (*S*)-**2**-derived monoterpenoids in CH_2Cl_2 (Bottle A1) or neat CH_2Cl_2 (Bottle B1) which then flowed into the Y-tube apparatus. Note: Volatile-rich air entering sidearms of the Y-tube olfactometer from Bottles A1 and B1 were switched between each experiment. (**C**) 3D-printed Y-tube apparatus showing entry ports for test bee and volatile-rich air from Bottles A1 and B1.

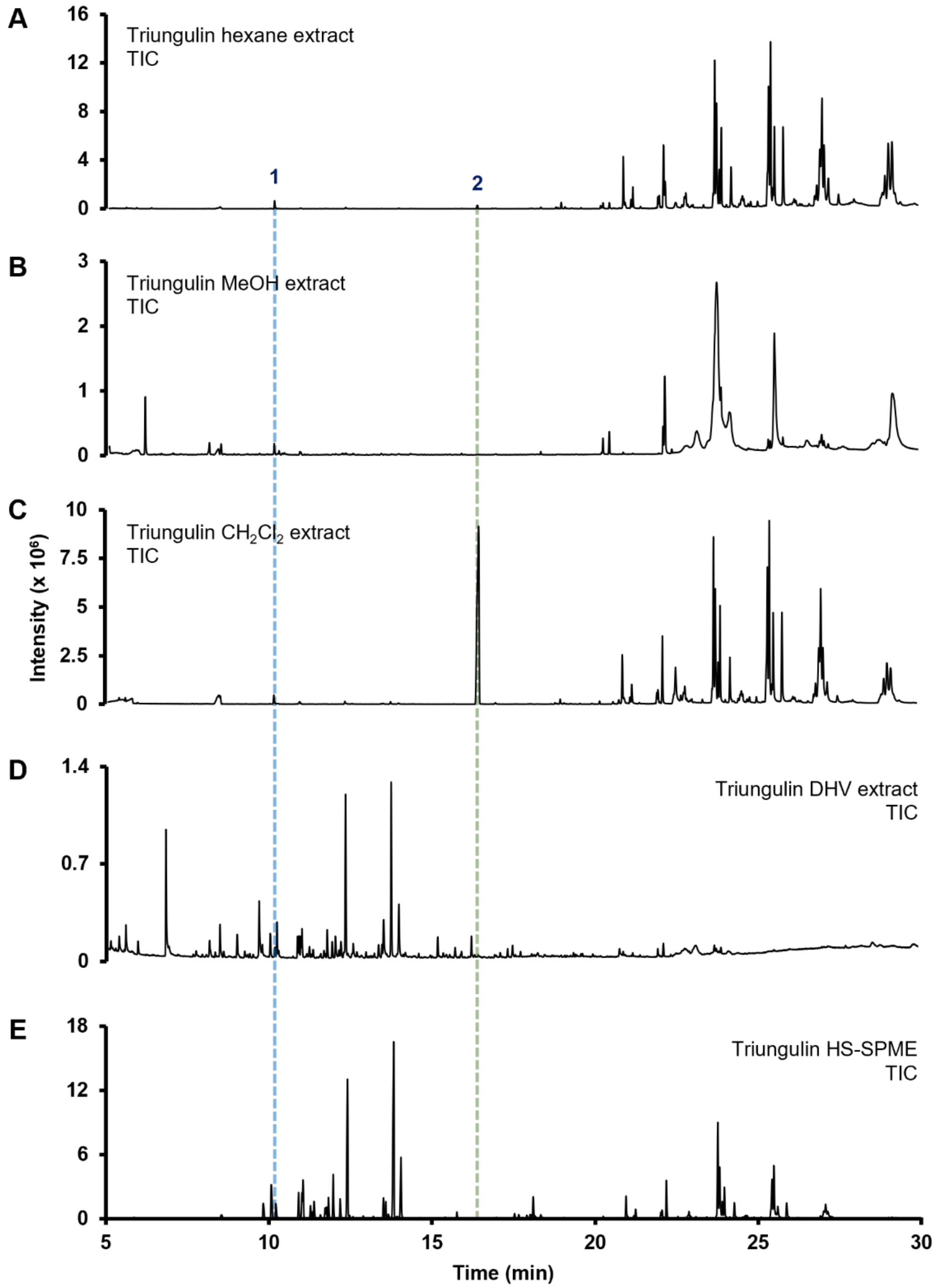

Supplementary Fig. 23. Comparison of triungulin volatile extraction methods.

Comparing GC-MS TIC data obtained for whole-body triungula extracts, using (**A**) hexane, (**B**) MeOH, and (**C**) CH_2Cl_2, with those obtained for (**D**) DHV collection and (**E**) HS-SPME sampling, show that solvent-based extraction methods fail to recover the complete monoterpenoid profile.

Peak identities: *1*) linalool, *2*) cantharidin*. Separation method: A. * tentatively identified using the National Institute of Standards and Technology (NIST) MS-Library v. 3.0 (2023).

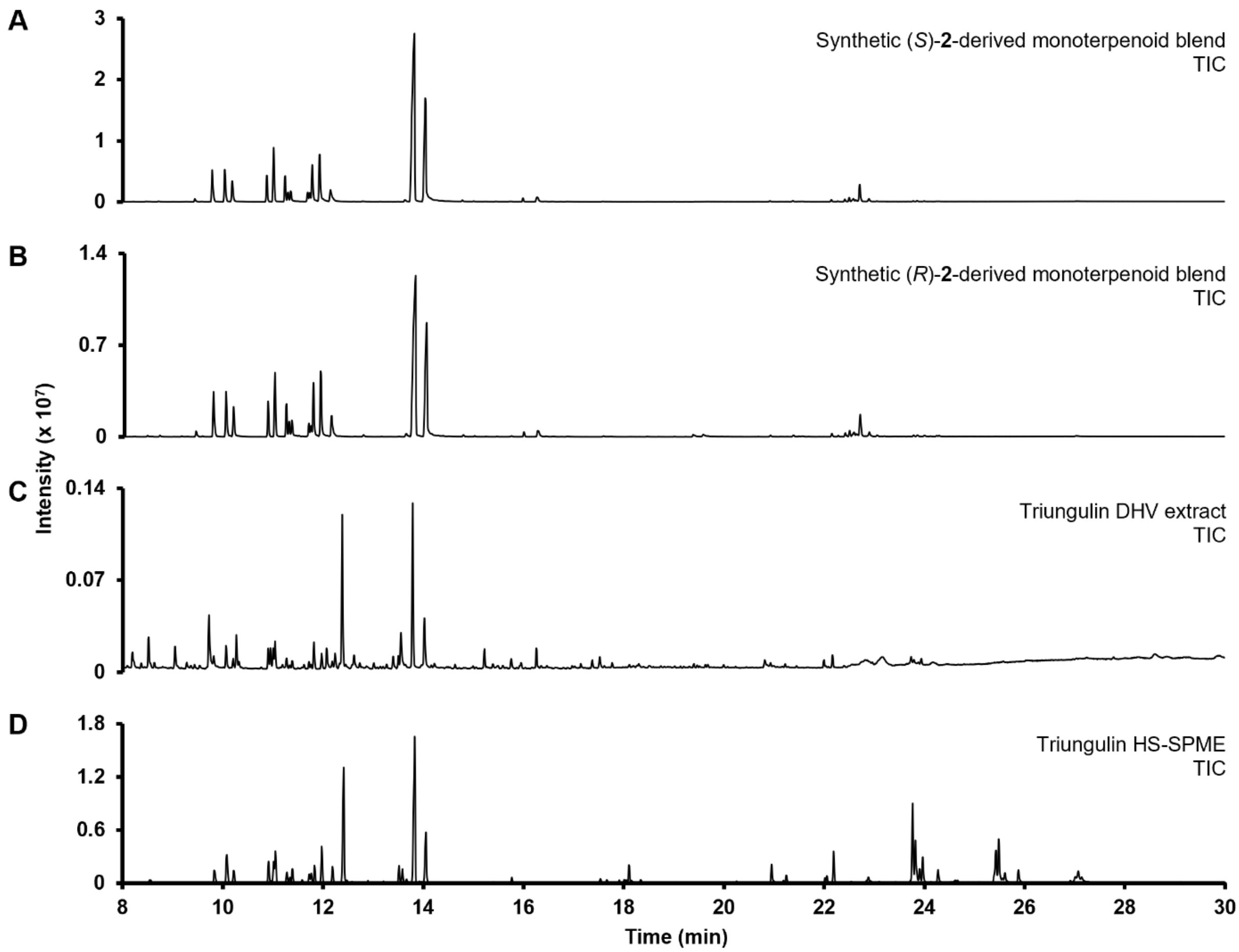

Supplementary Fig. 24. Comparison of synthetic linalool (2)-derived monoterpenoid blends with the naturally-occurring triungulin volatile profile.

Comparison of GC-MS data obtained for (**A**) (*S*)-**2**- and (**B**) (*R*)-**2**-derived synthetic monoterpenoid blends lacking long-chain hydrocarbons present to varying degrees in triungulin volatile profiles obtained using (**C**) DHV collection and (**D**) HS-SPME (t_r 20.95–29.22 min). Separation method: A.

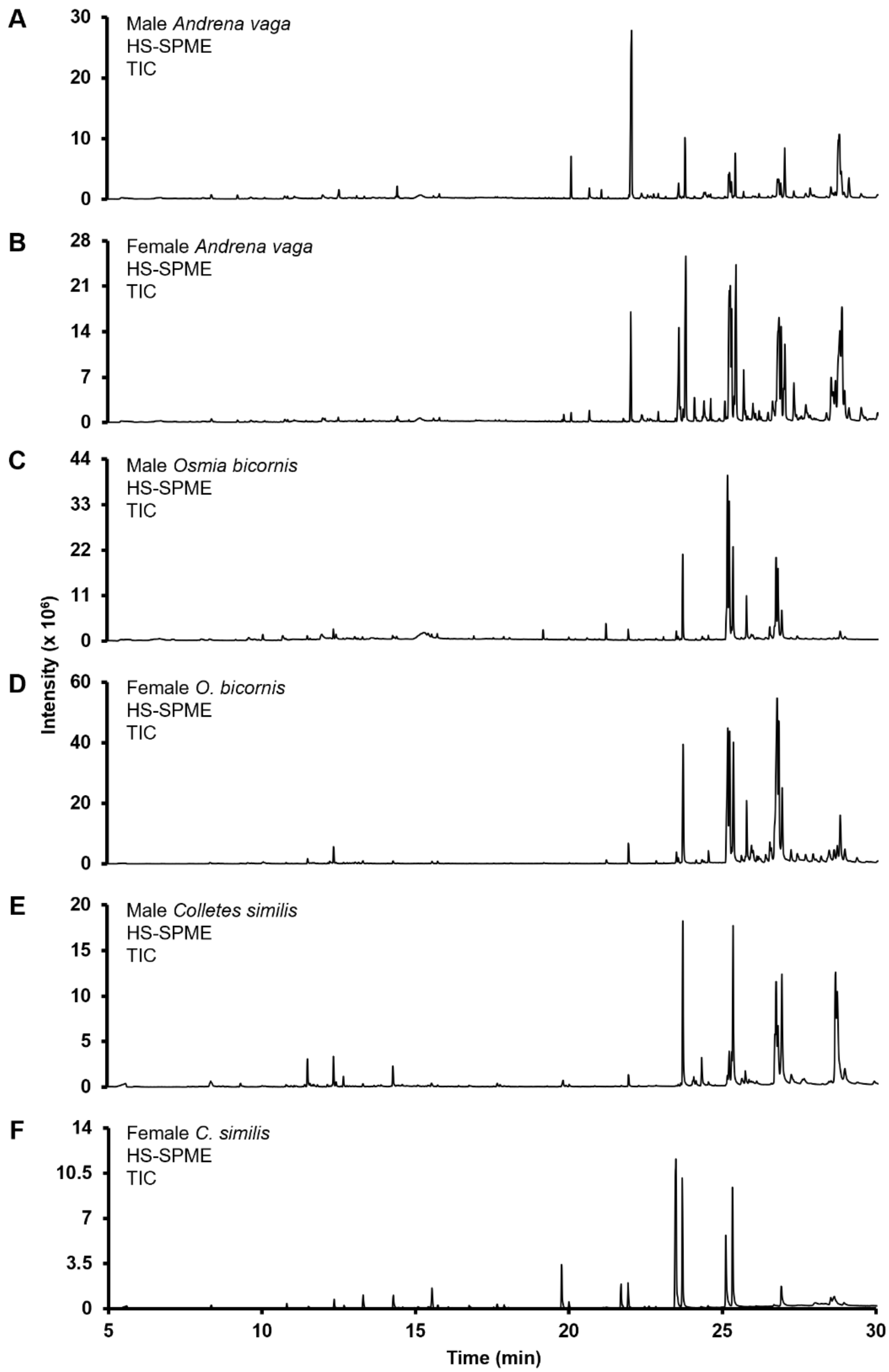

Supplementary Fig. 25. Volatile profiles of male and female *A. vaga*, *O. bicornis*, and *C. similis*.

HS-SPME GC-MS analysis of (**A**) male *A. vaga*, (**B**) female *A. vaga*, (**C**) male *O. bicornis*, (**D**) female *O. bicornis*, (**E**) male *C. similis*, and (**F**) female *C. similis* failed to detect any monoterpenoids volatiles. Separation method: A.

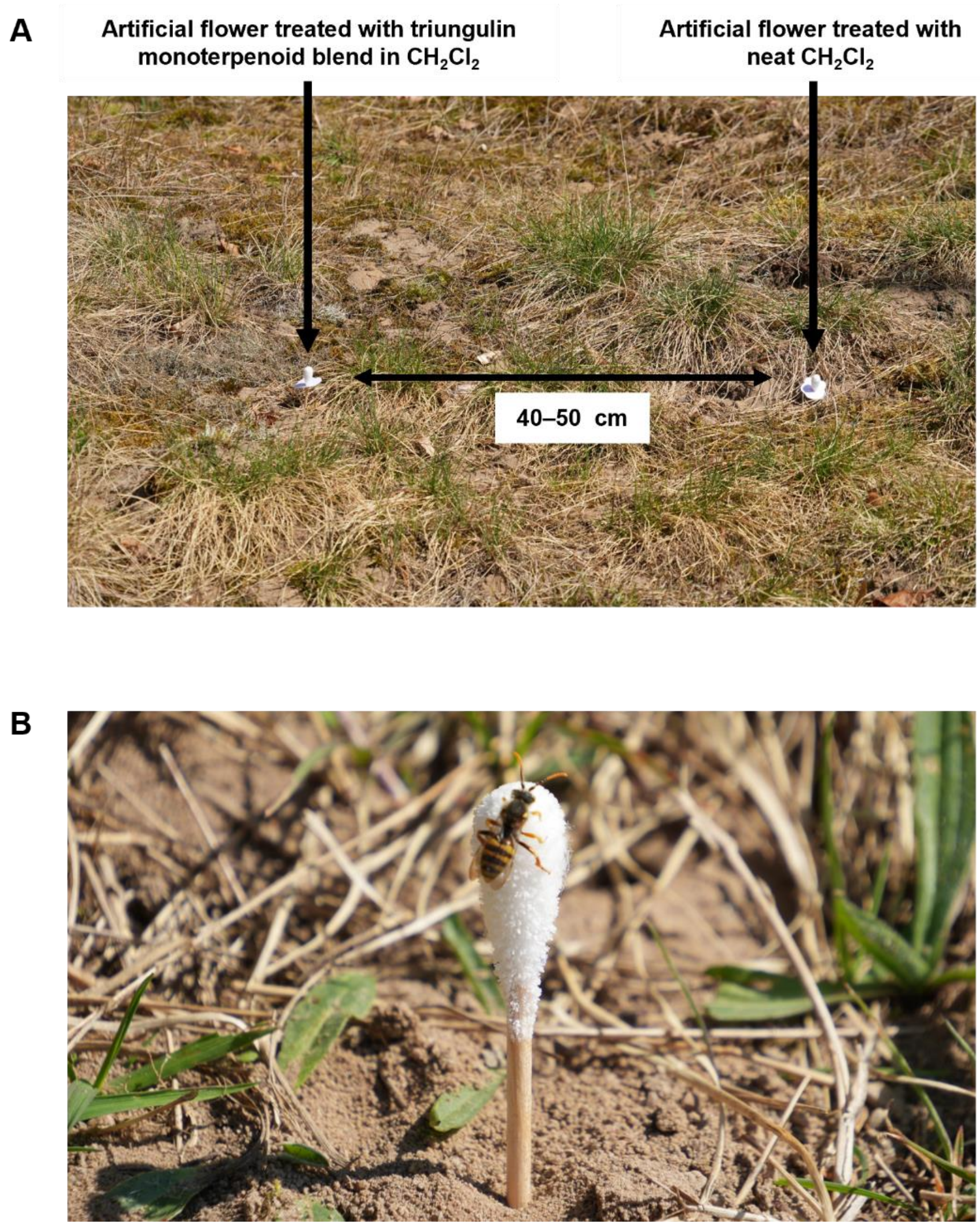

Supplementary Fig. 26. Observational field attraction assay assessing triungulin-derived monoterpenoids and long-chain hydrocarbon extracts.

(**A**) Field assay setup. (**B**) In addition to the attraction of wild *A*. *vaga*, artificial flowers treated with DHV extracts containing monoterpenoids were also attractive to a kleptoparasitic cuckoo bee species of the genus *Nomada*.

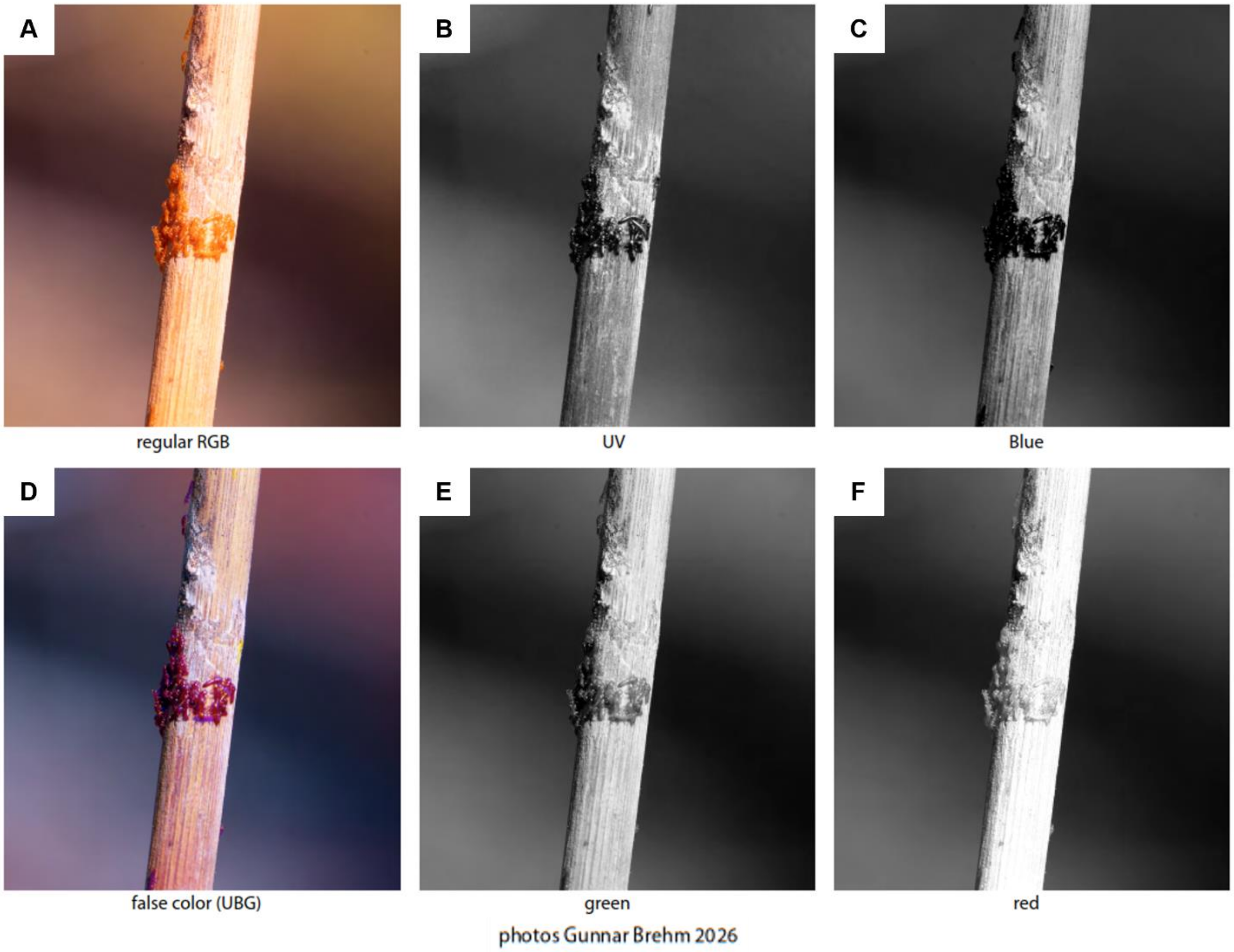

Supplementary Fig. 27. UV photography of *M. proscarabaeus* larval aggregation.

Photographic images of a single aggregation of *M. proscarabaeus* triungula on a wooden stick in multiple color spaces, including (**A**) regular red/green/blue (RGB), (**B**) ultraviolet (UV), (**C**) blue, (**D**) false color (ultraviolet/blue/green [UBG]), (**E**) green, and (**F**) red.

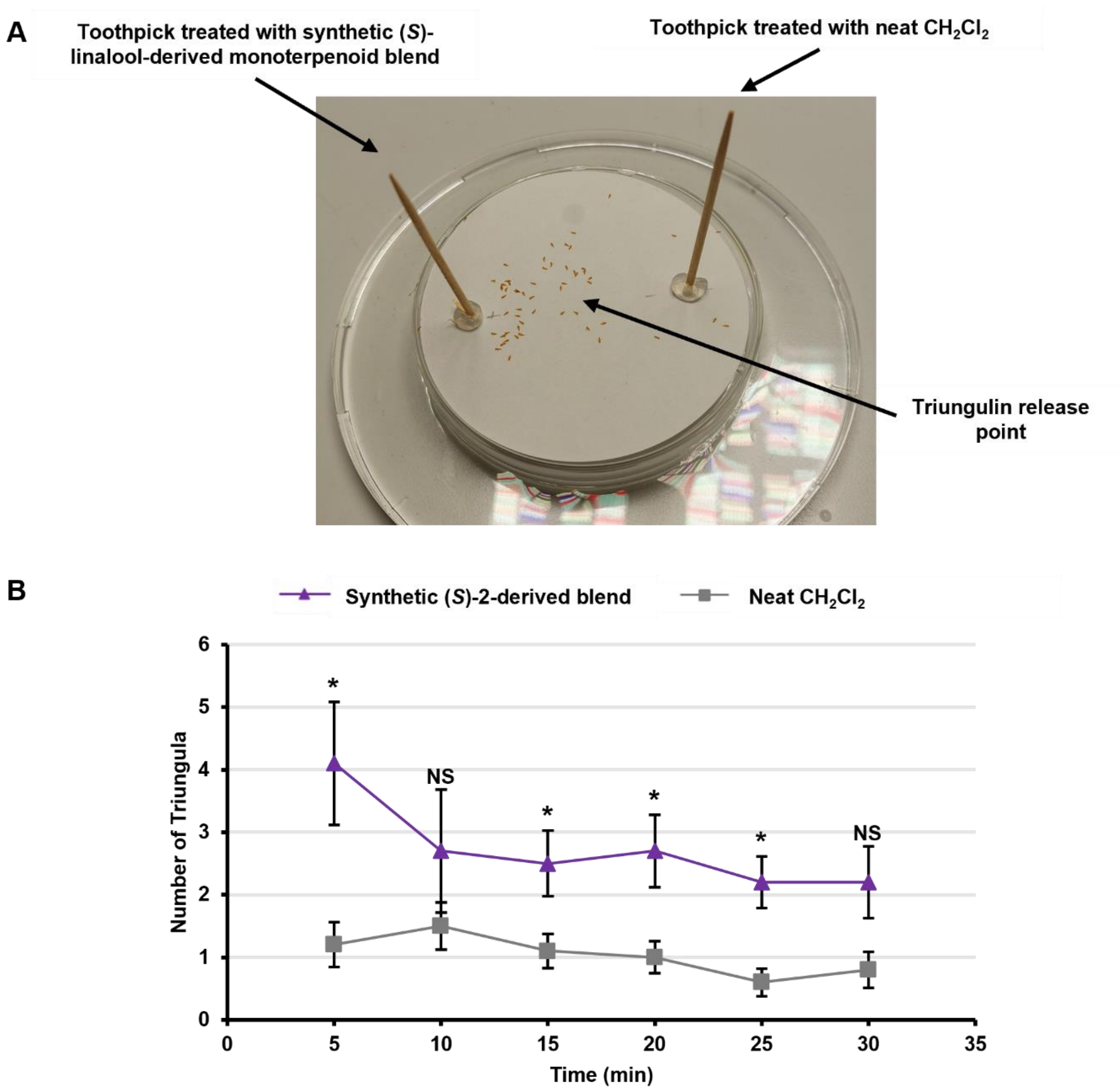

Supplementary Fig. 28. Olfactometric assays assessing triungulin preference for (*S*)-linalool-derived volatiles.

(**A**) An in-house setup was assembled to assess triungulin preference for a synthetic blend of (*S*)-linalool-derived monoterpenoids. (**B**) Line graph showing average number of triungula on a toothpick treated with a (*S*)-linalool-derived blend in CH_2Cl_2 versus a toothpick treated with neat CH_2Cl_2 after 5, 10, 15, 20, 25, and 30 min (error bars at each timepoint denote standard error of the mean [SEM]; n = 10). Paired t-test: paired two sample for means: *$p < 0.05$, NS = not significant. For further data, see **Supplementary Table 1**.

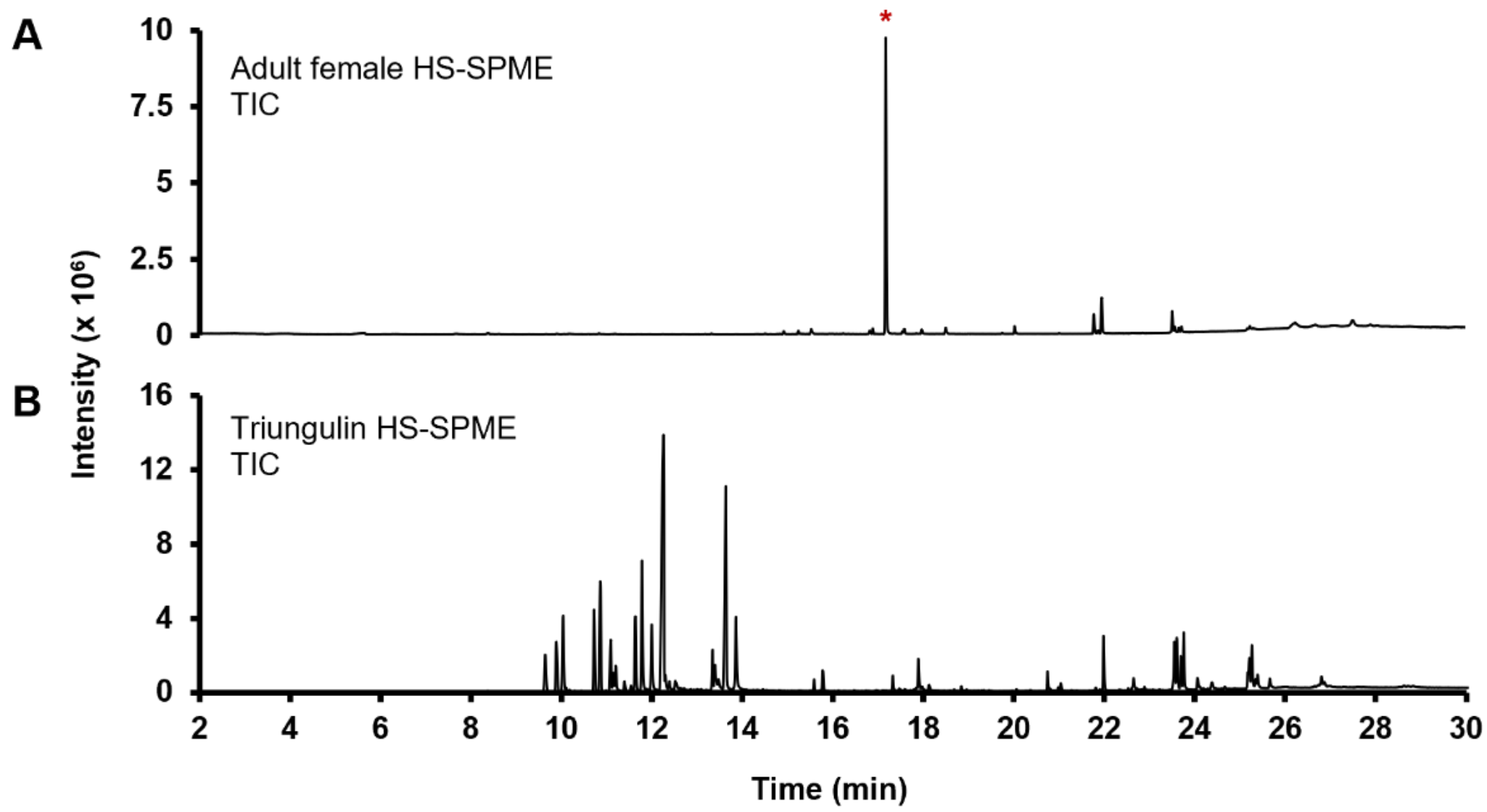

Supplementary Fig. 29. Comparison of volatile profiles for adult female *M. proscarabaeus* and triungula show that adult females do not emit monoterpenoid volatiles.

HS-SPME GC-MS data obtained for (**A**) adult female *M. proscarabaeus* and (**B**) triungula. Separation method: A. *Unidentified sesquiterpenoid volatile.

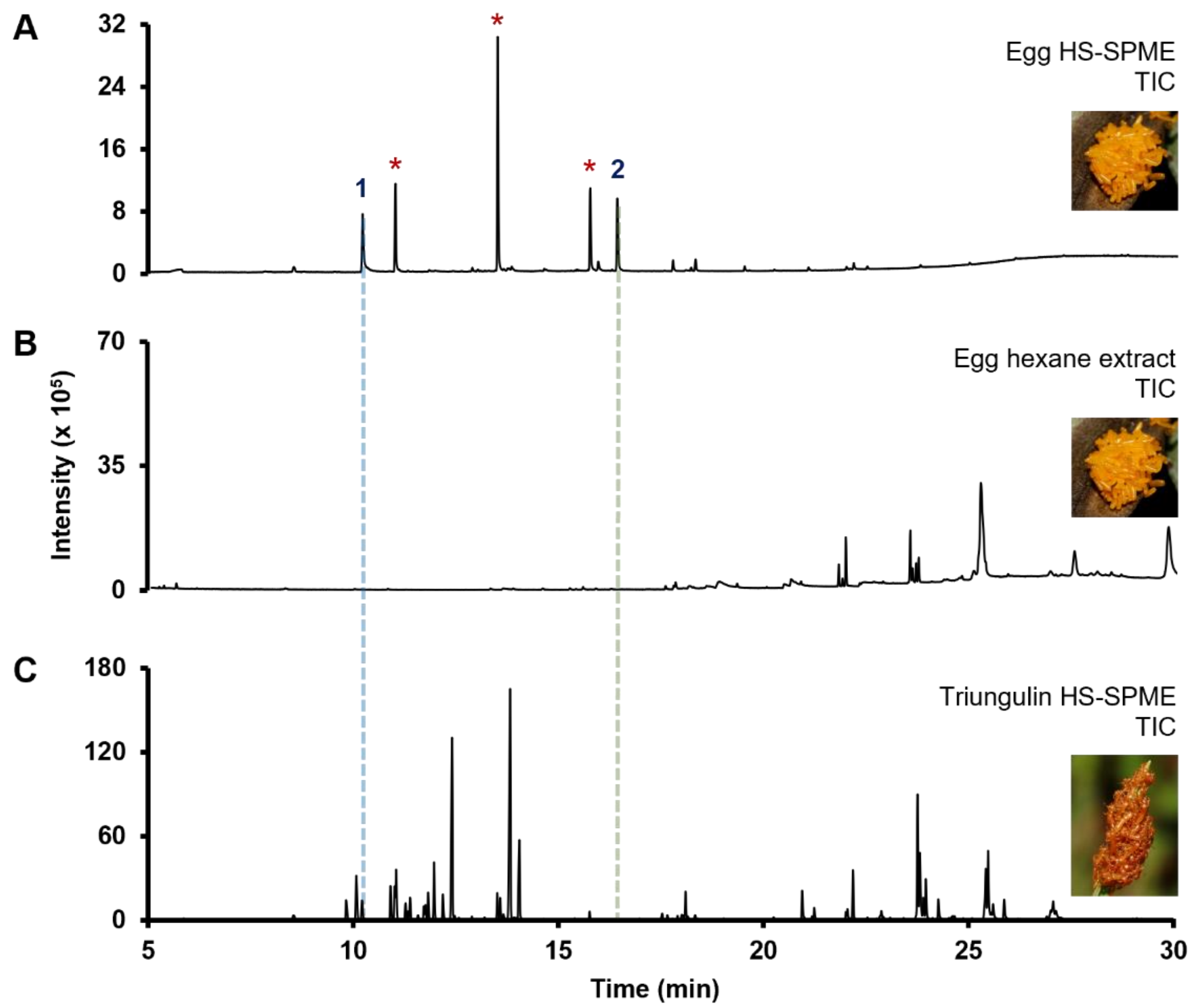

Supplementary Fig. 30. *M. proscarabaeus* triungula emit monoterpenoid bouquet following eclosion.

Comparison of GC-MS data obtained following (**A**) HS-SPME or (**B**) hexane extraction of eggs with (**C**) the volatile profile of triungula indicate that the only monoterpenoid emitted by eggs is linalool. Peak identity: *1*) linalool and *2*) cantharidin. Separation method: A. *Background.

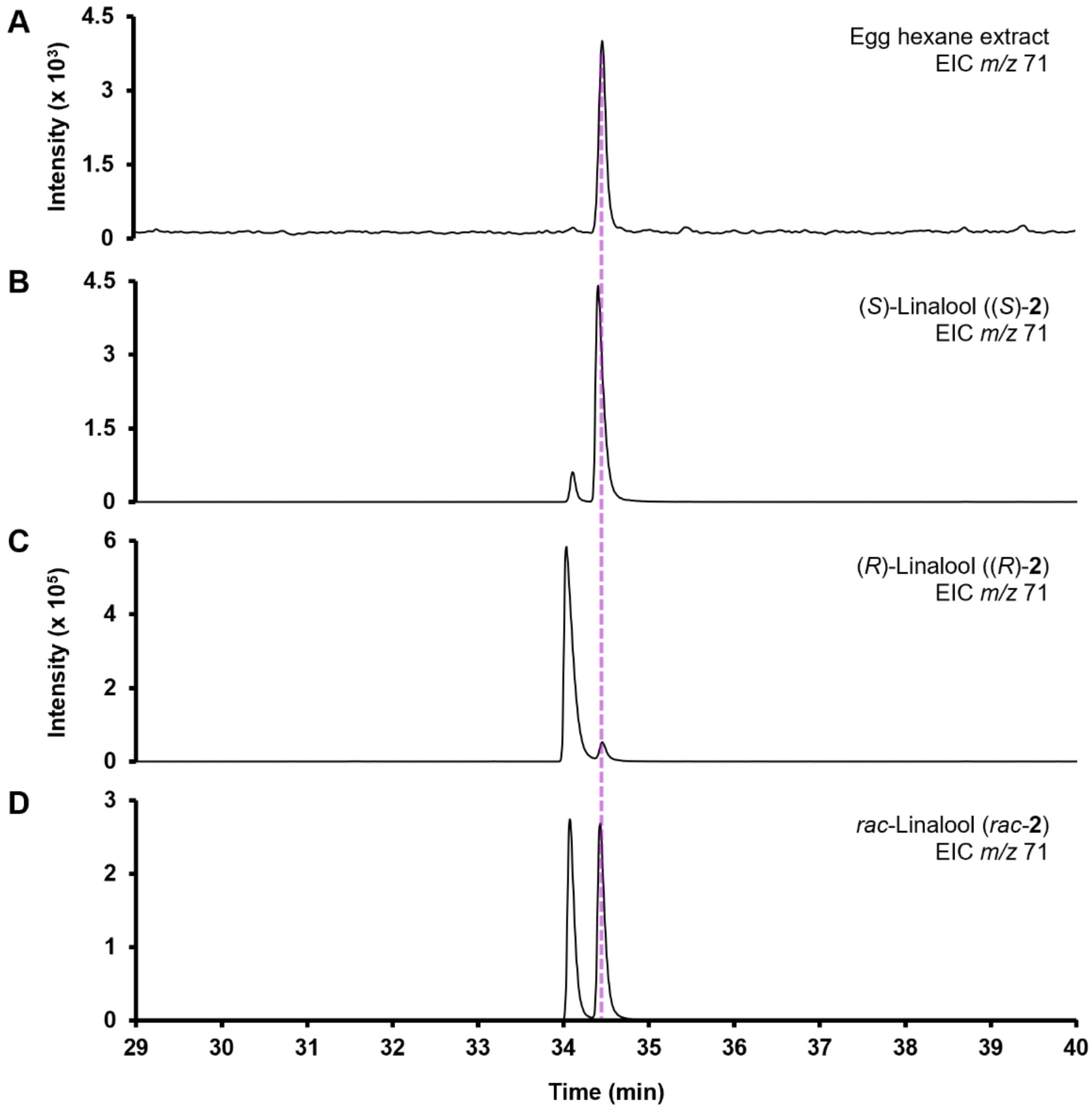

Supplementary Fig. 31. *M. proscarabaeus* eggs emit (*S*)-linalool ((*S*)-2).

Comparison of GC-MS data obtained for (**A**) a hexane extract from eggs, (**B**) (*S*)-**2**, (**C**) (*R*)-**2**, and (**D**) *rac*-**2**, showed that eggs emit (*S*)-**2** exclusively. Separation method: B.

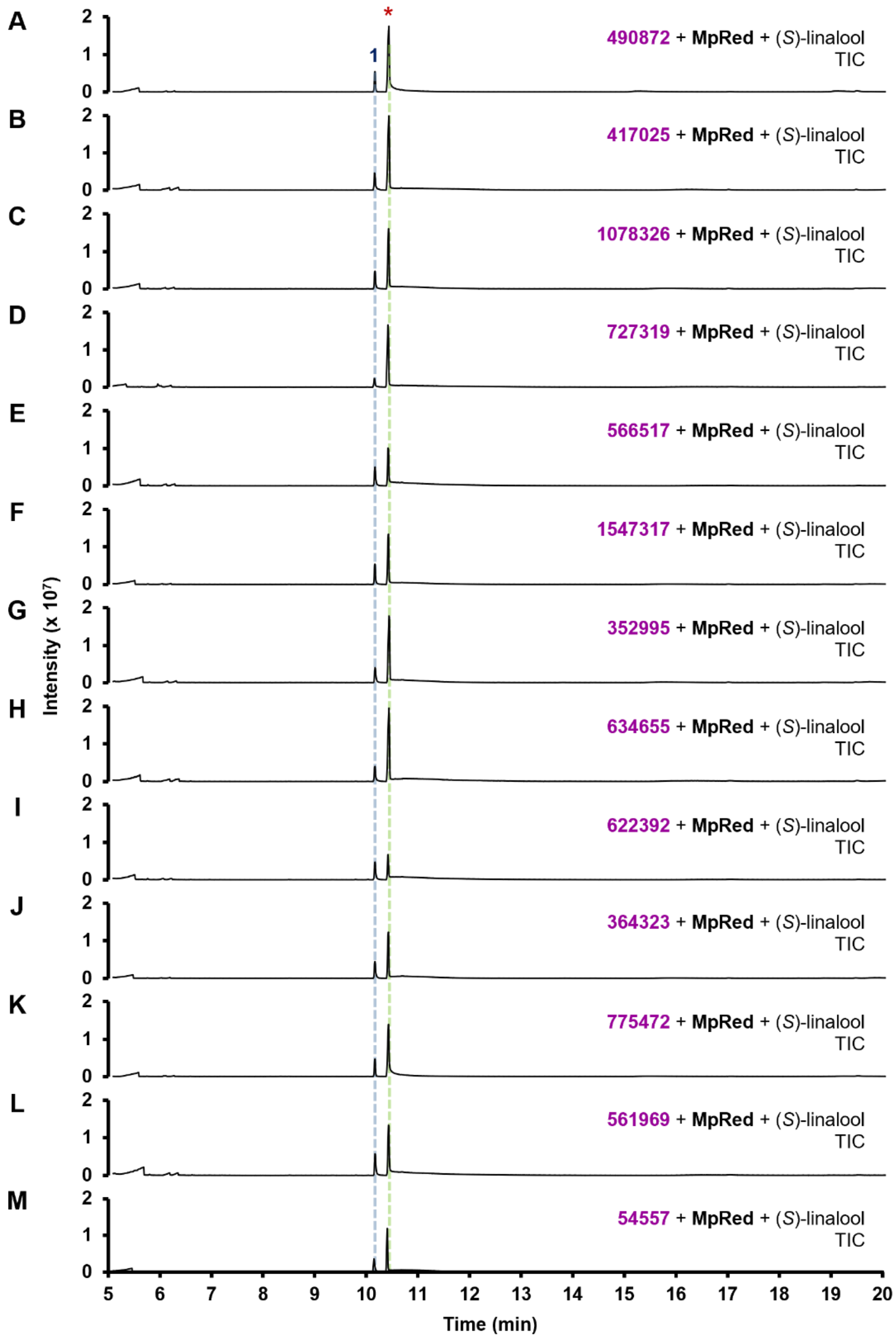

Supplementary Fig. 32. GC-MS data for *in vivo* assays with triungulin P450 gene candidates co-expressed with *MpRed* in *S. cerevisiae* in the presence of (*S*)-linalool.

Comparison of GC-MS data for P450 candidates (**A**) 490872, (**B**) 417025, (**C**) 1078326, (**D**) 727319, (**E**) 566517, (**F**) 1547317, (**G**) 352995, (**H**) 634655, (**I**) 622392, (**J**) 364323, (**K**) 775472, (**L**) 561969, and (**M**) 54557, revealed no detectable biocatalytic activity for the formation of

linalool-6,7-epoxide ((3*S*)-**5**) or (*S*)-8-hydroxylinalool ((*S*)-**8**) in the presence of (*S*)-linalool ((*S*)-**2**). Peak identity: *1*) (*S*)-linalool. Separation method: A. *Background.

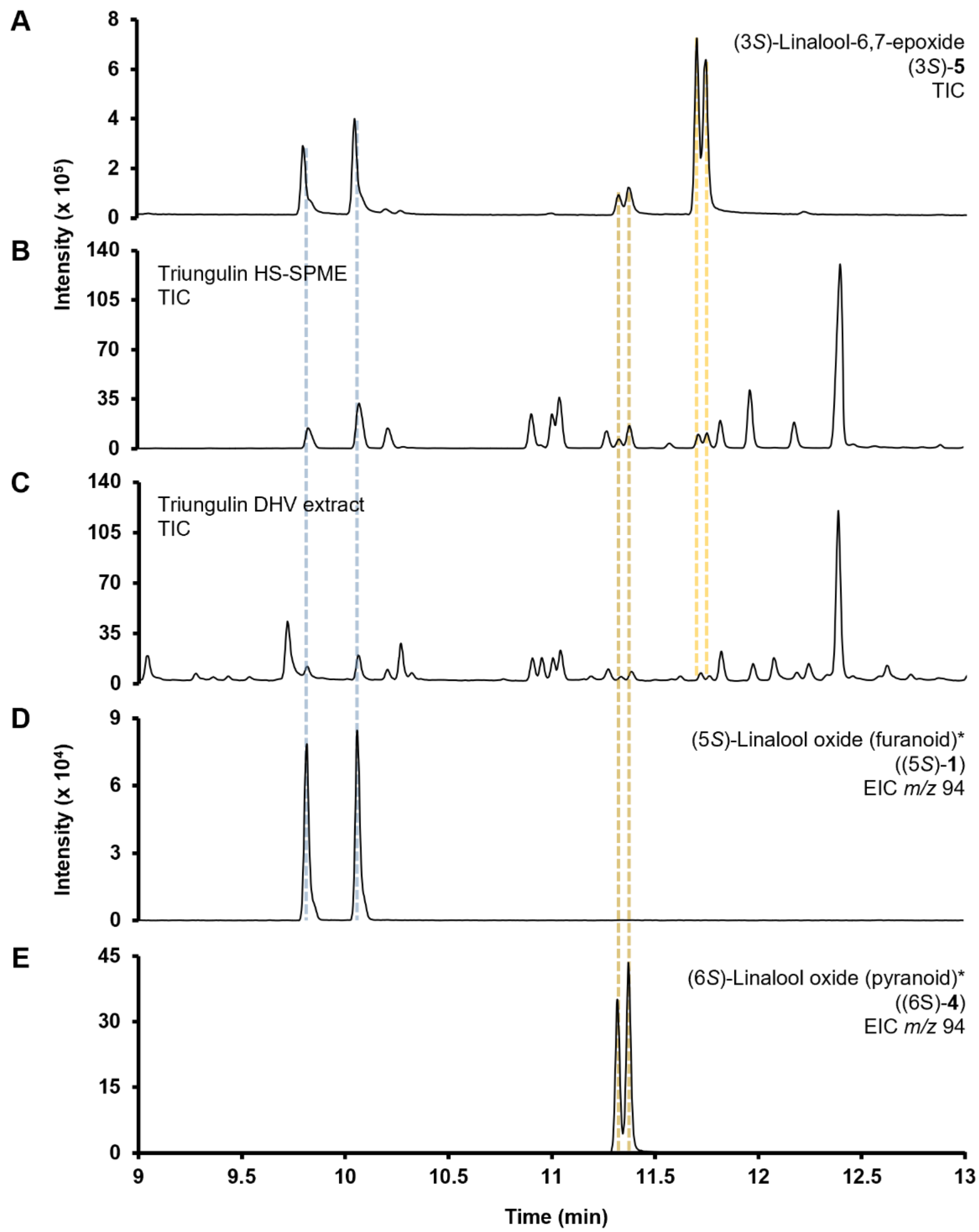

Supplementary Fig. 33. GC-MS data showing spontaneous cyclization of (3*S*)-linalool-6,7-epoxide ((3*S*)-5) to linalool oxides (2*S*)-1 and (6*S*)-4 during analysis.

Comparison of GC-MS data for (**A**) freshly prepared linalool-6,7-epoxide ((3*S*)-**5**), (**B**) triungulin volatiles collected by HS-SPME and (**C**) DHV collection, (**D**) (5*S*)-linalool oxide (furanoid)* ((5*S*)-**1**), and (**E**) (6*S*)-linalool oxide (pyranoid)* ((6*S*)-**4**). *Mixture of stereoisomers. Separation method: A.

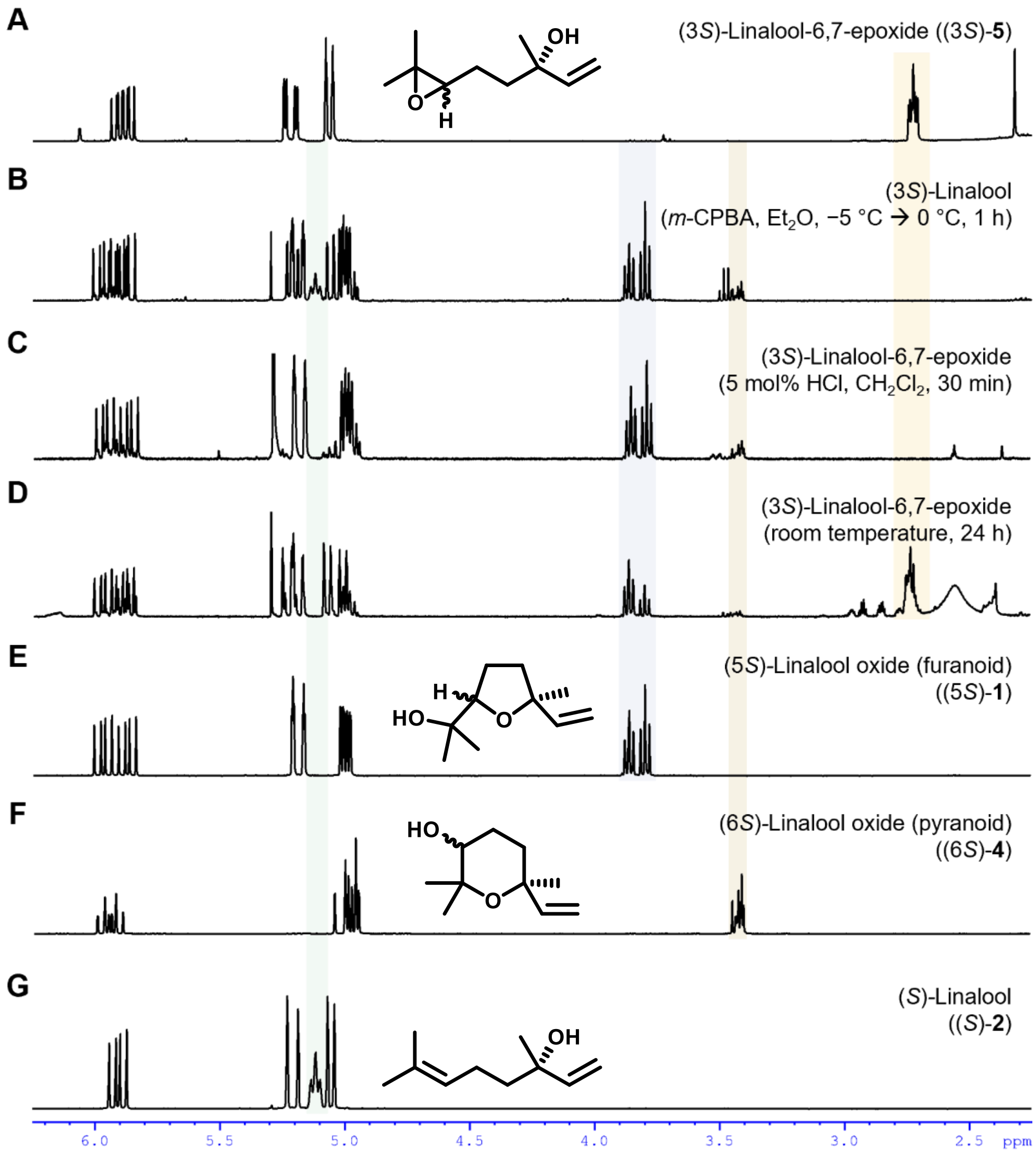

Supplementary Fig. 34. ^{1}H NMR data showing spontaneous cyclization of (3*S*)-linalool-6,7-epoxide ((3*S*)-5) to linalool oxides (2*S*)-1 and (6*S*)-4 under various conditions.

Comparison of ^{1}H NMR ($CDCl_3$, 400 MHz) spectral data for (**A**) freshly prepared (3*S*)-linalool-6,7-epoxide ((3*S*)-**5**), (**B**) the crude reaction mixture following attempted epoxidation of (*S*)-linalool ((*S*)-**2**) with *m*-chloroperoxybenzoic acid (1 eq. *m*-CPBA, CH_2Cl_2, Ar, −5 °C → 0 °C, 2 h), (**C**) the crude residue arising from Brønsted acid-catalyzed intramolecular cyclization of epoxide (3*S*)-**5** (5 mol% HCl, CH_2Cl_2, Ar, rt, 30 min), (**D**) epoxide (3*S*)-**5** stored at room temperature for 24 h, (**E**) (5*S*)-linalool oxide (furanoid)* ((5*S*)-**1**), (F) (6*S*)-linalool oxide (pyranoid)* ((6*S*)-**4**), and (G) (*S*)-linalool ((*S*)-**2**).

Supplementary Table 1. Y-tube dual-choice assay data for female *O. bicornis* between wheatgrass (control) and triungula on wheatgrass (treatment).

Replicate	Choice	Time (min:s)
1	T	1:26
2	T	0:09
3	T	1:14
4	T	0:58
5	T	0:29
6	T	1:06
7	T	1:04
8	T	0:25
9	C	0:48
10	T	1:07
11	T	0:10
12	T	1:47
13	T	0:26
14	T	1:13
15	C	1:10
16	T	1:26
17	T	0:58
18	T	1:11

Average choice time = 0:58 min:s; T = treatment; C = control.

Supplementary Table 2. Y-tube dual-choice assay data for male *O. bicornis* between wheatgrass (control) and triungula on wheatgrass (treatment).

Replicate	Choice	Time (min:s)
1	T	2:22
2	T	1:35
3	T	0:59
4	T	1:05
5	C	1:04
6	T	1:09
7	T	2:57
8	T	2:11
9	T	0:08
10	T	1:02
11	T	1:14
12	T	0:37
13	C	1:03
14	T	0:34
15	T	1:41
16	T	1:13

Average choice time = 1:18 min:s; T = treatment; C = control.

Supplementary Table 3. Y-tube dual-choice assay data for female *O. bicornis* between neat CH_2Cl_2 with wheatgrass (control) and triungulin DHV collection extract in CH_2Cl with wheatgrass (treatment).

Replicate	Choice	Time (min:s)
1	T	0:25
2	T	0:46
3	T	1:02
4	T	0:32
5	C	0:32
6	T	0:19
7	T	0:24
8	T	1:20
9	C	2:53
10	T	0:17
11	T	2:34
12	T	0:40
13	T	0:11
14	C	0:48
15	T	0:37
16	T	0:36
17	T	0:13
18	C	0:16
19	T	0:35
20	T	0:56

Average choice time = 0:49 min:s; T = treatment; C = control.

Supplementary Table 4. Y-tube dual-choice assay data for male *O. bicornis* between neat CH_2Cl_2 with wheatgrass (control) and triungulin DHV collection extract in CH_2Cl with wheatgrass (treatment).

Replicate	Choice	Time (min:s)
1	T	1:48
2	T	0:54
3	T	3:03
4	T	0:57
5	C	0:28
6	T	1:36
7	T	2:54
8	T	2:09
9	T	1:58
10	T	1:06
11	T	1:37
12	T	2:33
13	C	1:08
14	C	0:38
15	T	3:09
16	T	1:00
17	T	2:47
18	T	2:39

Average choice time = 1:48 min:s; T = treatment; C = control.

Supplementary Table 5. Y-tube dual-choice assay data for female *O. bicornis* between neat CH_2Cl_2 with wheatgrass (control) and a blend of synthetic (*S*)-linalool-derived monoterpenoids in CH_2Cl with wheatgrass (treatment).

Replicate	Choice	Time (min:s)
1	T	1:53
2	T	0:11
3	T	2:01
4	C	1:29
5	T	0:09
6	T	0:10
7	T	2:34
8	C	0:57
9	C	1:08
10	T	0:12
11	T	0:21
12	T	1:11
13	T	0:59
14	T	1:27
15	T	1:14
16	C	2:48
17	T	0:24

Average choice time = 1:07 min:s; T = treatment; C = control.

Supplementary Table 6. Y-tube dual-choice assay data for male *O. bicornis* between neat CH_2Cl_2 with wheatgrass (control) and a blend of synthetic (*S*)-linalool-derived monoterpenoids in CH_2Cl with wheatgrass (treatment).

Replicate	Choice	Time (min:s)
1	T	0:13
2	T	1:31
3	C	1:10
4	T	0:38
5	T	0:41
6	T	0:19
7	C	1:06
8	T	0:35
9	T	0:10
10	T	1:10
11	T	0:46
12	T	0:38
13	T	1:47
14	T	1:15
15	T	1:10
16	T	0:44
17	C	0:41
18	T	1:09

Average choice time = 0:52 min:s; T = treatment; C = control.

Supplementary Table 7. Y-tube dual-choice assay data for female *O. bicornis* between neat CH_2Cl_2 with wheatgrass (control) and a blend of synthetic (*R*)-linalool-derived monoterpenoids in CH_2Cl with wheatgrass (treatment).

Replicate	Choice	Time (min:s)
1	T	1:18
2	C	4:02
3	C	2:09
4	C	1:25
5	C	1:52
6	T	1:10
7	T	1:08
8	C	0:52
9	C	1:09
10	C	1:16
11	C	1:06
12	T	0:34
13	T	0:24
14	C	1:06
15	T	0:16
16	C	0:40

Average choice time = 1:16 min:s; T = treatment; C = control.

Supplementary Table 8. Y-tube dual-choice assay data for male *O. bicornis* between neat CH_2Cl_2 with wheatgrass (control) and a blend of synthetic (*R*)-linalool-derived monoterpenoids in CH_2Cl with wheatgrass (treatment).

Replicate	Choice	Time (min:s)
1	T	1:23
2	T	1:44
3	T	1:25
4	T	0:20
5	T	1:43
6	T	0:59
7	T	2:55
8	C	1:16
9	T	1:42
10	T	1:09
11	T	4:02
12	T	0:38
13	T	0:54
14	T	0:57
15	T	1:02
16	C	0:36

Average choice time = 1:25 min:s; *n* = 1, no choice; T = treatment; C = control.

Supplementary Table 9. Y-tube dual-choice assay data for female *C. similis* between neat CH_2Cl_2 with wheatgrass (control) and a blend of synthetic (*S*)-linalool-derived monoterpenoids in CH_2Cl with wheatgrass (treatment).

Replicate	Choice	Time (min:s)
1	T	1:19
2	T	1:11
3	T	1:17
4	T	1:08
5	T	0:58
6	C	1:31
7	T	1:46
8	T	0:58
9	T	0:48
10	T	1:07
11	C	1:10
12	T	2:03

Average choice time = 1:16 min:s; *n* = 1, no choice; T = treatment; C = control.

Supplementary Table 10. Y-tube dual-choice assay data for female *A. mellifera* between neat CH_2Cl_2 with wheatgrass (control) and a blend of synthetic (*S*)-linalool-derived monoterpenoids in CH_2Cl with wheatgrass (treatment).

Replicate	Choice	Time (min:s)
1	C	1:13
2	T	0:19
3	T	1:22
4	C	0:17
5	T	2:16
6	C	3:39
7	T	0:13
8	T	0:14
9	T	0:40
10	C	2:08
11	T	0:58
12	T	0:52
13	T	1:52
14	T	2:40
15	T	3:56
16	T	3:47
17	T	0:25
18	T	3:07
19	T	3:16
20	T	1:01
21	C	2:16
22	T	1:59

Average choice time = 1:45 min:s; T = treatment; C = control.

Supplementary Table 11. Y-tube dual-choice assay data for female *B. terrestris* between neat CH_2Cl_2 with wheatgrass (control) and a blend of synthetic (*S*)-linalool-derived monoterpenoids in CH_2Cl with wheatgrass (treatment).

Replicate	Choice	Time (min:s)
1	T	0:26
2	T	1:41
3	C	0:24
4	T	1:27
5	T	2:12
6	T	4:34
7	C	0:49
8	C	1:04
9	T	0:21
10	T	0:38
11	T	0:07
12	T	0:20
13	T	0:08
14	T	0:42
15	C	1:49
16	T	2:24
17	T	0:46
18	T	0:44
19	T	3:03
20	T	1:04

Average choice time = 1:45 min:s; T = treatment; C = control.

Supplementary Table 12. Y-tube dual-choice assay data for female *O. bicornis* between neat CH_2Cl_2 with wheatgrass (control) and synthetic (5*S*)-linalool oxide (furanoid) in CH_2Cl (1 mg/mL) with wheatgrass (treatment).

Replicate	Choice	Time (min:s)
1	T	0:34
2	C	0:45
3	T	0:35
4	C	0:56
5	T	1:27
6	C	2:20
7	T	0:33
8	T	0:18
9	C	0:09
10	T	0:44
11	T	0:38
12	C	0:46
13	C	0:15
14	C	0:09
15	T	0:49
16	T	1:47

Average choice time = 0.47 min:s; T = treatment; C = control.

Supplementary Table 13. Y-tube dual-choice assay data for male *O. bicornis* between neat CH_2Cl_2 with wheatgrass (control) and synthetic (5*S*)-linalool oxide (furanoid) in CH_2Cl (1 mg/mL) with wheatgrass (treatment).

Replicate	Choice	Time (min:s)
1	T	0:12
2	T	0:10
3	T	0:54
4	T	1:28
5	T	1:47
6	T	2:39
7	T	3:43
8	T	2:24
9	T	1:17
10	T	3:15
11	C	0:52
12	C	0:36
13	C	0:25
14	T	2:18
15	T	2:11
16	T	1:48

Average choice time = 1:37 min:s; T = treatment; C = control.

Supplementary Table 14. Y-tube dual-choice assay data for female *O. bicornis* between neat CH_2Cl_2 with wheatgrass (control) and synthetic (*S*)-linalool in CH_2Cl (1 mg/mL) with wheatgrass (treatment).

Replicate	Choice	Time (min:s)
1	T	0:07
2	T	1:46
3	T	0:52
4	C	2:00
5	C	1:52
6	T	1:05
7	C	1:31
8	C	2:14
9	T	0:43
10	T	0:18
11	T	1:58
12	T	0:14
13	C	2:21
14	T	1:32
15	T	0:48
16	T	0:42
17	T	0:33
18	T	1:44
19	T	1:31

Average choice time = 1:15 min:s; T = treatment; C = control.

Supplementary Table 15. Y-tube dual-choice assay data for male *O. bicornis* between neat CH_2Cl_2 with wheatgrass (control) and synthetic (*S*)-linalool in CH_2Cl (1 mg/mL) with wheatgrass (treatment).

Replicate	Choice	Time (min:s)
1	T	0:41
2	C	1:00
3	T	0:19
4	T	1:07
5	T	0:47
6	T	0:59
7	T	0:13
8	T	1:01
9	C	1:07
10	T	1:17
11	T	1:36
12	T	1:13
13	C	2:11
14	T	1:16
15	C	0:40
16	C	0:19
17	T	0:29
18	T	0:43
19	C	3:29

Average choice time = 1:04 min:s; *n* = 1, no choice; T = treatment; C = control.

Supplementary Table 16. Y-tube dual-choice assay data for female *O. bicornis* between neat CH_2Cl_2 with wheatgrass (control) and synthetic (5*S*)-lilac aldehyde in CH_2Cl (1 mg/mL) with wheatgrass (treatment).

Replicate	Choice	Time (min:s)
1	T	1:29
2	T	0:34
3	T	1:01
4	C	0:47
5	T	0:41
6	T	0:51
7	C	0:27
8	C	0:57
9	T	1:36
10	T	0:10
11	T	1:50
12	T	1:15
13	T	1:23
14	C	2:07
15	T	2:15
16	T	0:09
17	T	0:17
18	T	0:59

Average choice time = 1:02 min:s; T = treatment; C = control.

Supplementary Table 17. Y-tube dual-choice assay data for male *O. bicornis* between neat CH_2Cl_2 with wheatgrass (control) and synthetic (5*S*)-lilac aldehyde in CH_2Cl (1 mg/mL) with wheatgrass (treatment).

Replicate	Choice	Time (min:s)
1	T	2:33
2	C	0:40
3	T	1:26
4	T	1:04
5	T	2:05
6	T	3:42
7	T	0:34
8	T	1:08
9	T	1:19
10	T	0:51
11	T	1:14
12	C	0:36
13	T	1:40
14	T	0:43
15	T	2:13
16	C	1:14
17	T	1:38
18	T	0:12

Average choice time = 1:22 min:s; T = treatment; C = control.

Supplementary Table 18. Y-tube dual-choice assay data for female *O. bicornis* between neat CH_2Cl_2 with wheatgrass (control) and synthetic (6*S*)-linalool oxide (pyranoid) in CH_2Cl (1 mg/mL) with wheatgrass (treatment).

Replicate	Choice	Time (min:s)
1	C	0:22
2	T	1:02
3	T	0:51
4	T	0:40
5	T	2:09
6	C	0:54
7	T	0:55
8	C	1:30
9	T	1:00
10	T	1:20
11	T	0:43
12	T	0:50
13	T	0:40
14	T	1:27
15	T	0:09
16	T	0:21

Average choice time = 0:55 min:s; T = treatment; C = control.

Supplementary Table 19. Y-tube dual-choice assay data for male *O. bicornis* between neat CH_2Cl_2 with wheatgrass (control) and synthetic (6*S*)-linalool oxide (pyranoid) in CH_2Cl (1 mg/mL) with wheatgrass (treatment).

Replicate	Choice	Time (min:s)
1	T	4:41
2	C	1:24
3	T	3:22
4	T	0:36
5	T	0:43
6	T	1:20
7	C	0:42
8	T	3:24
9	T	0:14
10	T	2:01
11	C	1:55
12	T	3:16
13	T	2:34
14	T	2:05
15	T	1:34
16	T	1:10

Average choice time = 1:56 min:s; T = treatment; C = control.

Supplementary Table 20. Y-tube dual-choice assay data for female *O. bicornis* between neat CH_2Cl_2 with wheatgrass (control) and synthetic (3*S*)-linalool-6,7-epoxide in CH_2Cl (1 mg/mL) with wheatgrass (treatment).

Replicate	Choice	Time (min:s)
1	T	4:58
2	T	2:08
3	C	1:46
4	T	0:29
5	T	0:36
6	T	1:11
7	C	2:31
8	C	0:55
9	T	3:15
10	T	1:12
11	T	0:22
12	T	4:54
13	C	2:55
14	T	1:01
15	T	1:25
16	T	0:33

Average choice time = 1:53 min:s; T = treatment; C = control.

Supplementary Table 21. Y-tube dual-choice assay data for male *O. bicornis* between neat CH_2Cl_2 with wheatgrass (control) and synthetic (3*S*)-linalool-6,7-epoxide in CH_2Cl (1 mg/mL) with wheatgrass (treatment).

Replicate	Choice	Time (min:s)
1	T	2:44
2	T	1:04
3	C	0:27
4	T	0:14
5	T	1:08
6	C	1:11
7	T	1:58
8	T	3:59
9	T	1:39
10	T	2:14
11	T	0:23
12	C	0:26
13	C	2:56
14	T	4:02
15	T	4:32
16	C	3:40

Average choice time = 2:02 min:s; *n* = 2, no choice; T = treatment; C = control.

Supplementary Table 22. Y-tube dual-choice assay data for female *O. bicornis* between neat CH_2Cl_2 with wheatgrass (control) and synthetic (5*S*)-lilac alcohol in CH_2Cl (1 mg/mL) with wheatgrass (treatment).

Replicate	Choice	Time (min:s)
1	T	0:43
2	T	1:09
3	T	0:44
4	T	0:49
5	T	1:35
6	T	0:34
7	T	0:36
8	T	0:28
9	T	1:06
10	T	0:21
11	T	0:20
12	T	0:31
13	T	0:59
14	T	0:32
15	C	0:24
16	T	0:52
17	T	0:09
18	T	0:26

Average choice time = 1:41 min:s; T = treatment; C = control.

Supplementary Table 23. Y-tube dual-choice assay data for male *O. bicornis* between neat CH_2Cl_2 with wheatgrass (control) and synthetic (5*S*)-lilac alcohol in CH_2Cl (1 mg/mL) with wheatgrass (treatment).

Replicate	Choice	Time (min:s)
1	T	0:44
2	T	1:30
3	C	0:27
4	T	1:06
5	C	0:57
6	T	0:23
7	T	0:37
8	T	0:33
9	T	1:32
10	T	1:17
11	C	0:17
12	C	2:03
13	T	0:52
14	C	0:43
15	T	1:35
16	T	0:31
17	T	0:36
18	T	2:20

Average choice time = 1:00 min:s; T = treatment; C = control.

Supplementary Table 24. Y-tube dual-choice assay data for female *O. bicornis* between neat CH_2Cl_2 with wheatgrass (control) and synthetic (*S*)-8-oxolinalool in CH_2Cl (1 mg/mL) with wheatgrass (treatment).

Replicate	Choice	Time (min:s)
1	T	0:38
2	T	1:26
3	T	0:29
4	T	0:08
5	T	1:07
6	T	0:09
7	T	1:33
8	T	1:46
9	T	1:36
10	T	0:17
11	T	0:29
12	T	1:21
13	T	0:17
14	T	1:45
15	C	0:57
16	T	1:56

Average choice time = 1:24 min:s; T = treatment; C = control.

Supplementary Table 25. Y-tube dual-choice assay data for male *O. bicornis* between neat CH_2Cl_2 with wheatgrass (control) and synthetic (*S*)-8-oxolinalool in CH_2Cl (1 mg/mL) with wheatgrass (treatment).

Replicate	Choice	Time (min:s)
1	T	1:34
2	T	2:34
3	T	0:44
4	T	0:52
5	T	1:03
6	T	1:44
7	T	0:28
8	T	1:13
9	T	1:46
10	T	1:31
11	T	0:36
12	T	2:07
13	C	0:58
14	T	3:08
15	T	0:08
16	T	2:04

Average choice time = 0:59 min:s; T = treatment; C = control.

Supplementary Table 26. Y-tube dual-choice assay data for female *O. bicornis* between neat CH_2Cl_2 with wheatgrass (control) and synthetic (*S*)-8-hydroxylinalool in CH_2Cl (1 mg/mL) with wheatgrass (treatment).

Replicate	Choice	Time (min:s)
1	T	0:54
2	T	1:51
3	C	0:26
4	T	0:56
5	C	1:23
6	C	0:30
7	T	0:51
8	T	0:37
9	T	1:46
10	C	0:15
11	T	0:57
12	C	0:33
13	T	0:26
14	C	0:39
15	C	0:27
16	T	0:15
17	T	0:50
18	C	0:55
19	T	1:01

Average choice time = 1:02 min:s; T = treatment; C = control.

Supplementary Table 27. Y-tube dual-choice assay data for male *O. bicornis* between neat CH_2Cl_2 with wheatgrass (control) and synthetic (*S*)-8-hydroxylinalool in CH_2Cl (1 mg/mL) with wheatgrass (treatment).

Replicate	Choice	Time (min:s)
1	T	0:37
2	T	0:09
3	T	1:08
4	T	2:30
5	T	0:34
6	T	1:40
7	T	0:18
8	T	0:47
9	T	0:32
10	T	0:29
11	T	0:42
12	T	1:21
13	T	1:45
14	T	2:30
15	T	0:22
16	T	1:18

Average choice time = 0:49 min:s; T = treatment; C = control.

Supplementary Table 28. Y-tube dual-choice assay data for female *O. bicornis* between two identical wheatgrass plants (controls 1 and 2).

Replicate	Choice	Time (min:s)
1	C2	0:36
2	C2	0:45
3	C2	1:37
4	C1	0:26
5	C2	0:48
6	C2	0:21
7	C1	2:19
8	C2	0:34
9	C1	1:41
10	C1	0:14
11	C2	0:32
12	C1	1:24
13	C1	0:55
14	C1	0:10
15	C2	1:31
16	C2	0:24
17	C1	0:08
18	C1	0:33

Average choice time = 1:51 min:s; C1 = control 1; C2 = control 2.

Supplementary Table 29. Y-tube dual-choice assay data for male *O. bicornis* between two identical wheatgrass plants (controls 1 and 2).

Replicate	Choice	Time (min:s)
1	C1	2:46
2	C2	3:06
3	C2	0:50
4	C2	2:23
5	C1	1:59
6	C2	2:40
7	C2	2:17
8	C2	1:04
9	C1	2:39
10	C1	2:34
11	C1	1:30
12	C2	2:04
13	C2	1:26
14	C1	1:53
15	C2	1:20
16	C2	0:16
17	C1	0:59
18	C1	1:47

Average choice time = 0:49 min:s; C1 = control 1; C2 = control 2.

Supplementary Table 30. Triungulin olfactometric choice assay results.

	Time (min)											
	5*		10NS		15*		20*		25*		30NS	
	Treat	Con	Treat	Con	Treat	Con	Treat	Con	Treat	Con	Treat	Con
**Entry**	.	.	.	.	.	.	.	.	.	.	.	.
1	0	3	0	3	0	2	0	2	0	1	0	2
2	0	0	0	0	3	0	4	0	4	0	4	0
3	5	0	2	0	2	0	2	0	2	0	2	0
4	1	0	2	0	1	1	1	1	1	0	1	2
5	6	3	4	2	3	2	3	1	3	1	3	0
6	10	1	2	2	2	0	2	1	2	0	1	0
7	6	1	5	1	6	2	5	2	4	1	6	1
8	4	1	5	3	2	1	2	0	2	0	1	0
9	5	1	5	2	2	1	2	1	1	1	1	1
10	4	2	2	2	4	2	6	2	3	2	3	2

Paired t-test: paired two sample for means: *$p < 0.05$, NS = not significant. Treat. = treatment ((*S*)-linalool-derived monoterpenoid blend in CH_2Cl_2); Con. = Control (neat CH_2Cl_2).

Supplementary Table 31. Primers used for coding sequence amplification from triungulin cDNA.

Gene	Direction	Note	Sequence
MpRed	Forward	*Sal*I	ACCGTCGACATGGACGAAACAGAAGCG
	Reverse	*Nhe*I	GTCGCTAGCTTAACTCCAAACATCAGCTGA
CYP347BT1	Forward	*Not*I	CACTAAAGGGCGGCCGCAATGTTAATAGTTTTGGTTAATGTCTG
	Reverse	*Sac*I	GAATTGTTAATTAAGAGCTCCTAAACTGCGTTGATTCTTAGAG
CYP345BZ1	Forward	*Not*I	CACTAAAGGGCGGCCGCAATGATTTTGATAAGTCTAGTTCTAGTATT
	Reverse	*Sac*I	GAATTGTTAATTAAGAGCTCTTATAGTTCCCTTTCTTCAAAATTAATC

Supplementary Table 32. Sequences amplified from triungulin cDNA for this study.

Gene	Sequence
MpRed	ATGGACGAAACAGAAGCGAAAATTGGTGCCCAACAAATACCTGAAACTGTTGAAAG<br>TTTATTTACTACATTAGATTATATATTACTTGCCCTTTTAATTGGTGGTGTAAGTTATT<br>GGTATTTTAATCGGCAGAAAAAGAAAGAAGTAACAACTACGCGATCCTATACTATAC<br>AGCCAACTTCTATGACATTACAAGCAACAACAGAGAGTTCATTTATAAAAAAATTAAA<br>AGCATCAGGACGTTCATTGGTTGTATTTTATGGTAGTCAAACTGGTACTGGTGAAGA<br>ATTTGCTGGACGTTTAGCTAAAGAAGGTTTACGTTATGGTATGAAAGGGATGATAGC<br>TGATCCAGAAGAATGCGATATGGAGGAATTAGTGAATTTAAAAGCAATACCAAATTC<br>ATTAGCTGTATTTTGTATGGCAACATATGGTGAAGGTGACCCAACTGATAATGCAAT<br>GGAATTTTATGAATGGTTACAAAATGGTGATGCTGATTTAACTGGCTTAAATTATTCG<br>GTATTCGGCTTAGGTAATAAAACGTATGAACATTACAATGAAGTCGCAATCTACATA<br>GATAAACGTTTAGAAGATTTAGGAGCAACTCGAGTGTTTGATTTAGGACTTGGCGAT<br>GATGATGCTAATATTGAAGACGATTTCATCACTTGGAAAGATAAGTTCTGGCCAGCA<br>GTTTGTGAATTCTTCGGTATCGAGTCAACTGGTGAAGATATCAGTATACGTCAATAT<br>CGCTTACAAGAATTCACTGATGAATTACCAGAACGTCTTTATACAGGTGAAATGGCA<br>CGATTGCATTCACTTAAAAATCAAAGACCACCTTATGATGCAAAGAATCCATTTTTGG<br>CGAAAATTTTAGTAAATCGTGAATTACATAAAAATGGTGATCGTTCATGTATGCATAT<br>CGAATTTGATATTAGCGGTTCAAAGATGCGTTATGATTCTGGCGATCATTTAGCCGT<br>TTATCCAATTAATAATCATGAATTGGTTGAGAAAATTGGCAAATATACTGGTCAAGAT<br>TTAGATACTGTGTTCACTTTAGTAAATACTGATGAAGAATCAAGTAAAAAACATCCAT<br>TCCCCTGTCCAACATCTTATCGTACAGCTTTAACACATTATCTTGATATCACAATGAA<br>TCCAAGAACGCACGTTTTAAAAGAATTATCGGAATATTGTAGTGATCCAAATGAAAA<br>AGAAAAACTAAAATTAATGGCCAGTATATGTCCAGAAGGTAAAGCATTATATCAACA<br>ATGGATAAACGATGATAATCGTAATATTGCACAAATATTAGAAGATATGCCGTCGTG<br>TCGGCCAGCTTTAGATCATTTATGTGAATTATTACCACGTTTACAACCACGTTATTAT<br>TCAATATCATCATCTGCAAAACTATATCCAAATACTGTACATATAACAGCTGTTGTTG<br>TCGAATATAAAACACCAACAGGACGTATTAATAAAGGTGTTGCAACAACATGGTTAG<br>CAACTAAACAACCTAAACAAAATCAATTAGATTTACCAACTGCACCAATATTTATACG<br>TAAATCACAATTTCGTTTACCAACAAAAACACAAACACCAATTATTATGATTGGTCCT<br>GGTACTGGTTTGGCACCGTTCCGTGGTTTTATACAAGAACGCAATCTAGCTAATGAT<br>GAAGGTAAAACTGTTGGCGAAACAGTTTTATATTTCGGTTGTAGAAAACGTACGGAA<br>GATTTCTTATATGAAGACGAATTAGTGAAATATGAAAAGGATGGTATCATTAAATTAC<br>ATATTGCTTTCAGTCGTGATCAACCACAAAAAGTATATGTTACACATTTAGTTGAACA<br>AAATGCTGATGAAATTTGGAGGATTATCGGTGAAAATAATGGACATCTCTATATTTG<br>CGGTGATGCTAAATCGATGGCATCAGATGTACGTAATATTGTTTTGAAAATATTTAAA

	GAAAAAGGTCAAATGACGGAAGAACAAGCTTTAGCGTATTTGAAGAAGATGGAAAC ACAAAAACGTCTATCAGCTGATGTTTGGAGTTAA
CYP347BT1	ATGATTTTGATAAGTCTAGTTCTAGTATTGTTAATTACAGTTTATTTTCTGTTAAAACG TAAATATGAATATTGGAGAAAACGTGGAATACCTGGCCCTCAACCACGTTTCATTAC TGGTAATGTTGGATCAATGCTATTAATGAAACAATCATTAGCTGATATTATTTCAAAA TTATACAACGAATATCACAATGTTTCTTTAGTGGGAATGTTTAAAGGCCTATCGCCAA CATTACTTGTTCGTGATCCTGATCTAATTAAAGATATATTAATCAAAGATTTTAGTTAT TTCCACGATAATGATTTAGACGTAAGTGAATCTGTTGATCCATTACTTGGTCGAAAT CCATTTGTATTAAAAGGTGAGAAATGGAAAATTGTACGTACACTACTAACACCTTGC TTCACCAGTGGAAAGATGAAATCTATTCATCCTTTATTAGAAGATATCAGTCAAAAAC TTGTAAAATATATTAAACGACAATTAGATGGGAATCGACGGAATGGTCTTGAACTTA AAGAATTATGTAAAAGTTATACATTAGAAACAGTAGCTAGTTGTGCATTTGGTTTAGA AGGGAAATGTTTCGAAGATGAAAATTCTGAATTTCGACAAATTGCAAACAAATTTTTT GTACCAGGTGGATGGACAAATTTTATTATGATTTTATCAAGTATAATACCATCATTAC CTACATTATTTCGTGTAAAAGTGGTACTGAAAGAAGTTGAAGATCGTCTCATTGCAT TAACAAGTGAAACAATAAAATATCGTAAAGAAAACAACATTGAACGAAATGATTTTAT TCAAATTGTTAGTAAATTAAAAGAAACATCAAAAAATTATGAATTTACTGATGTTGAT GTATGTGCCCAAGCAGCGTCTTTTTTCGTTGATGGTTTTGAAACGACAGCAGTTACA ATGAGTTTCCTTCTATTTCACTTAGCTGATAATCCACATATTCAAGAAAAGCTTCGTG AAGAGATTGATGAACAATTCCGACAAAATAATGGAAAATTAACTTATGATAGTATTCA ACAAATGGCATATCTTGATGCTGCACTTAATGAATCGCAGCGAATTCATCCAGCTGC ACAAATATTGTTGAAACGTTGCACACGAAATTATAAATATATTCCGAAAGCGAATGAT GGATTTGATAAACCACTTGAAATCGAGGCGGGAACTAGCCTGATAATATCAATAAG GAATTTACACAATGATCCCAAATACTTCAATGATCCAAGTGTATACAATCCGGATAG ATTTTTGGGTAAAAACAAAGAAAACTTGAATAAAAATGTATTCTTTCCATTTGGAGAT GGCCAACGGGTTTGTTTAGGTCAAAGATTTGCTCTTCTTCAAATAAAAATAGGAATT GCTTATATTATACGTAATTTCAAACTGTCTGTGAATAGCAAAACACAGAAACCTATAA AGCTTGATCCTCTCTATCTGATGCCAGCACCTATTGGGGGATTTTGGATTAATTTTG AAGAAAGGGAACTATAA
CYP345BZ1	ATGTTAATAGTTTTGGTTAATGTCTGTATTGTGTTGTCACTTATTTATTATCTTTTGAA GAGGAATAATAACTATTGGAAATCACGAGGTGTTCCTCAAGATAAGCCATTTCTATT GTTTGGAAGTTTCTACAATGTAGTTGCAGGAAAACAAAGTATACTTCAACGCATCAC TGAAATCTACCAAAATTACAGAGATTTACCATATTTTGGTTTTTATACATTCACAAAAC CAGTATTAATGATAAACAATCCTGATATTATCAAACGTATATTAGTGAAAGATTTTGA TCAATTTGCTGATCGTAATGTTCATACAAATGAAAAAAGTGATCCCATTGGACATCAT

TCAATGTTTACCACACGCGGTAAGACATGGCGTAGTCTACGTACGAAAATATCACC
GATTTTCACATCCGGCAAGATGAAGATGATATTGCCATTAATGATGGAATGTGGTGA
AAATATGTATCGAATTTTGAAAAATTCGGAACGCTCAATAGTTGATGTGCAAGATTTA
ATAAAACGATATTCAGTTGATGTCATATCATCATGTGCTTTTGGTATAAATGCAAATA
GTTTAAATAGTCAAAATGGTGGAATATTAGGTGCTGCTGAAAAATTAATGGATCAAA
ATTCATTCAAACGCAGTTTTTCATTGTTTTGCTTCTTTTTCGTACCGAAATTGGCTAA
TATTCTACATTTGAAAATGTTCGATATGACGGCTGCCAATTTTCTAAGAAATATCTTC
ACAACAACATTAGATGAAAGGGAGAAATTGAATATTCAACGGACTGATTTGATTGAT
TTATTGAATCAATTGAAGAAAACTCAATCGATTTCTGATGAATATAAATTTGATGATG
ATAAATTAGCAGCACAAGCTATGACTTTTTTCAGTGCTGGTAATGAAACAACATCAA
CAACTATTGCTTCTACTTTACATGAATTAGCTTTAGATAAAGCTGTACAAAGTCGATT
AAGAGATGAAATTCGGGAATCTTATGAAACGAATAAAGGCTTCACTTATGAAGGAAT
CCAAGAAATGAAATACCTAGATATGGTCTTTAATGAAACCCTCAGACGGTACCCAGT
GACAATATTTCTAACACGAGAAGCTATTGACGATTATGTTATTGAGGAGACTGGATT
TAAAATTGAAAAAGGTACATCGATCATGATACCGGTGTGTGGTTTACATTTCGATCC
TGAATATTTCCCAAATCCACAAAAATTCGATCCGGAACGTTATAGTGATGAAAACAA
AATTAATATTAAACCGTATACGTATATGCCTTTCGGAGAAGGTCCACGTAACTGTATT
GGTCGACGATTTGGTTTATTATCAGCAAAAATTGCACTTGTTCATCTACTGAAAGAC
TTTGAATATGATGTGGCTGAAGACACACCAGTTCCATTAAAACTCAAACCTAAAGCT
ATTTTGTTACAACACGATGGTGGAATTCCTCTAAGAATCAACGCAGTTTAG

Supplementary Table 33. Comparison of absolute gene expression values (RPKM) for larvae and adult females.

Gene ID	Gene annotation	Absolute Expression (RPKM)	
		Larvae	Adult
490872	P450	965.0	16.7
417025	P450	575.8	0.9
1078326	P450	154.9	11.0
727319	P450-like	99.9	0.0
983480	P450 (***CYP347BT1***)	76.4	2.7
566517	P450	76.1	4.3
1547317	P450	57.0	7.9
352995	P450	51.5	0.6
634655	P450	50.4	3.6
622392	P450-like	46.2	6.3
364323	P450-like	42.8	5.2
775472	P450-like	20.1	0.5
325667	P450 (***CYP345BZ1***)	18.8	0.1
561969	P450	18.2	0.1
54557	P450-like	16.8	0.1
240516	P450 reductase (***MpRed***)	15.6	0.6

References

58. Dorta, R.L., Rodríguez, M.S., Salazar, J., Suárez, E. 1,3-Transposition of primary allylic alcohols: Synthesis of optically active secondary and tertiary allylic alcohols. *Tetrahedron Lett*. **38**, 4675–4678 (1997).
59. Liu, Z., Lan, J., Li, Y., Xing, Y., Cen, W. Total partial synthesis of (3*S*, 6*S*)-(+)-3,7-dimethyl-6-hydroxy-3-acetoxyocta-1,7-diene and (3*S*, 6*S*)-(–)-3,7-dimethylocta-1,7-diene-3,6-diol from geraniol. *J. Chem. Res. Synop*. **1999**, 324–325.
60. Riclea, R., Dickschat, J.S. The absolute configuration of the pyrrolosesquiterpenoid glaciapyrrol A. *Chem. Eur. J*.**17**, 11930–11934 (2011).
61. Yamazaki, S. An effective procedure for the synthesis of acid-sensitive epoxides: Use of 1-methylimidazole as the additive on methyltrioxorhenium-catalyzed epoxidation of alkenes with hydrogen peroxide. *Org. Biomol. Chem.***8**, 2377–2385 (2010).
62. Wan, K.K., Litz, J.P., Vosburg, D.A. Two-step, stereoselective synthesis of linalyl oxides by asymmetric allylic *O*-alkylation. *Tetrahedron Asymmetry*. **21**, 2425–2428 (2010).
63. Y. Zhu, *et al*. Enantiomeric and quantitative analysis of volatile terpenoids in different teas (*Camellia sinensis*). *J. Chromatogr. A.* **1490**, 177–190 (2017).
64. Vidari, G., Di Rosa, A., Zanoni, G., Bicchi, C. Enantioselective synthesis of each stereoisomer of the pyranoid linalool oxides: the linalool route. *Tetrahedron Asymmetry*. **10**, 3547–3557 (1999).
65. Chakraborti, A.K., Gulhane, R. Perchloric acid adsorbed on silica gel as a new, highly efficient, and versatile catalyst for acetylation of phenols, thiols, alcohols, and amines. *Chem. Commun*. **2003**, 1896–1897.
66. Bonnländer, B., Baderschneider, B., Messerer, M., Winterhalter, P. Isolation of two novel terpenoid glucose esters from Riesling wine. *J. Agric. Food Chem.* ***46***, 1474–1478 (1998).
67. Sköld, M., Börje, A., Harambasic, E., Karlberg, A-T. Contact allergens formed on air exposure of linalool. Identification and quantification of primary and secondary oxidation products and the effect on skin sensitization. *Chem. Res. Toxicol.***17**, 1697–1705 (2004).
68. Wakayama, S., Namba, S., Hosoi, K., Ohno, M. The synthesis and the absolute configurations of lilac alcohols, new naturally occurring odorous ingredients of Lilac flower. *Bull. Chem. Soc. Jpn.* **46**, 3183–3187 (2006).
69. Kreck, M., Mosandl, A. Synthesis, structure elucidation, and olfactometric analysis of lilac aldehyde and lilac alcohol stereoisomers. *J. Agric. Food Chem.* **51**, 2722–2726 (2003).
